# Fam40b is required for lineage commitment of murine embryonic stem cells

**DOI:** 10.1038/cddis.2014.273

**Published:** 2014-07-10

**Authors:** V Wagh, M X Doss, D Sabour, R Niemann, K Meganathan, S Jagtap, J A Gaspar, M A Ardestani, S Papadopoulos, M Gajewski, J Winkler, J Hescheler, A Sachinidis

**Affiliations:** 1 Center of Physiology and Pathophysiology, Institute of Neurophysiology, University of Cologne, Robert-Koch-Str. 39, Cologne 50931, Germany; 2 Center of Physiology and Pathophysiology, Institute of Vegetative Physiology, University of Cologne, Robert-Koch-Str. 39, Cologne 50931, Germany; 3 Institute for Genetics, University of Cologne, Cologne, Germany

## Abstract

FAM40B (STRIP2) is a member of the striatin-interacting phosphatase and kinase (STRIPAK) complex that is involved in the regulation of various processes such as cell proliferation and differentiation. Its role for differentiation processes in embryonic stem cells (ESCs) is till now completely unknown. Short hairpin RNA (shRNA)-mediated silencing of Fam40b expression in ESCs and differentiating embryoid bodies (EBs) led to perturbed differentiation to embryonic germ layers and their derivatives including a complete abrogation of cardiomyogenesis. Pluripotency factors such as Nanog, Oct4 and Sox2 as well as epigenetic factors such as histone acetyltransferase type B (HAT1) and DNA (cytosine-5)-methyltransferase 3-*β* (Dnmt3b) were highly upregulated in Fam40b knockdown EBs as compared with control and scrambled EBs. To examine the relevance of Fam40b for development *in vivo*, Fam40b was knocked down in developing zebrafish. Morpholino-mediated knockdown of Fam40b led to severe abnormalities of the cardiovascular system, including an impaired expression of ventricular myosin heavy chain (*vmhc*) and of cardiac myosin light chain 2 (*cmlc2*) in the heart. We identified the gene product of Fam40b in ESCs as a perinuclear and nucleolar protein with a molecular weight of 96 kDa. We conclude that the expression of Fam40b is essential for the lineage commitment of murine embryonic stem cells (mESCs) into differentiated somatic cells via mechanisms involving pluripotency and epigenetic networks.

The analysis of mammalian transcriptomes, including humans and mice, led to the identification of thousands of novel transcripts of (as yet) unknown function (TUFs).^1, 2, 3, 4, 5, 6^ Although it has recently been confirmed that TUFs, including noncoding RNAs, can participate in the regulation of biological and cellular processes, the functional role of most TUFs remains to be elucidated.^1, 2, 3, 4, 5, 6^ The expression of many TUFs during the development is often transient and it has only recently been recognized that TUFs are indeed not just transcriptional noise but that many of them play a critical role during development.^7, 8, 9^ Recently, TUFs with distinct transcriptional kinetics during osteogenic and adipogenic differentiation of human mesenchymal stem cells (hMSCs) have been identified. These include a long nuclear noncoding RNA, a micro-RNA host gene and a novel small protein gene. All three were transcriptionally regulated by Wnt and protein kinase A (PKA) signaling pathways that are the key pathways for hMSC differentiation.^1^

Recently, increasing attention has been directed toward identifying and understanding the function and intracellular signaling pathways of the STRIPAK complex in regulating biological processes of multiple organisms.^10, 11, 12^ There is growing evidence that the TUF Fam40b (synonyms: striatin interacting protein 2 (STRIP2) or D330017J20Rik) is a member of the striatin-interacting phosphatase and kinase (STRIPAK) complex that is involved in the regulation of cell growth, proliferation, cell migration and adhesion, neural and vascular development as well as cardiac function.^10, 11, 12^ Fam40a (STRIP1) and Fam40b directly interact with the striatin family members proteins, thereby regulating the STRIPAK complex activity. Striatin, a key core of the STRIPAK complex, is highly expressed in the central and peripheral nervous systems and in heart muscle, testis and lymphocytes (reviewed in Hwang and Pallas^10^). Interestingly, Fam40b is also upregulated in the mouse heart, brain, adipose tissues, eye, testis and seminal vesicles (http://www.ncbi.nlm.nih.gov/geo/tools/profileGraph.cgi?ID=GDS3142:1433847_at). Similar results have been obtained in human tissues, showing a high expression level in brain, heart and skeletal muscle (http://www.ncbi.nlm.nih.gov/geo/tools/profileGraph.cgi?ID=GDS423:43524_at). Therefore, Striatin and Fam40B might play an important role for several biological processes such as differentiation, cell growth, development and differentiation. Embryonic stem cells (ESCs) offer an excellent model to identify TUFs because developmental processes *in vivo* can be recapitulated *in vitro* to a certain extent in a time- and cost-effective manner while minimizing animal experiments.^13^ Here we demonstrated that Fam40b encodes for a protein with a molecular weight of 96 kDa that is mainly localized in cytoplasm, predominantly in the perinuclear region, and in nucleoli of undifferentiated mESCs and is essential for their early and late lineage commitment.

## Results

### Constitutive knockdown of Fam40b in differentiating ESCs

**Figure 1:**
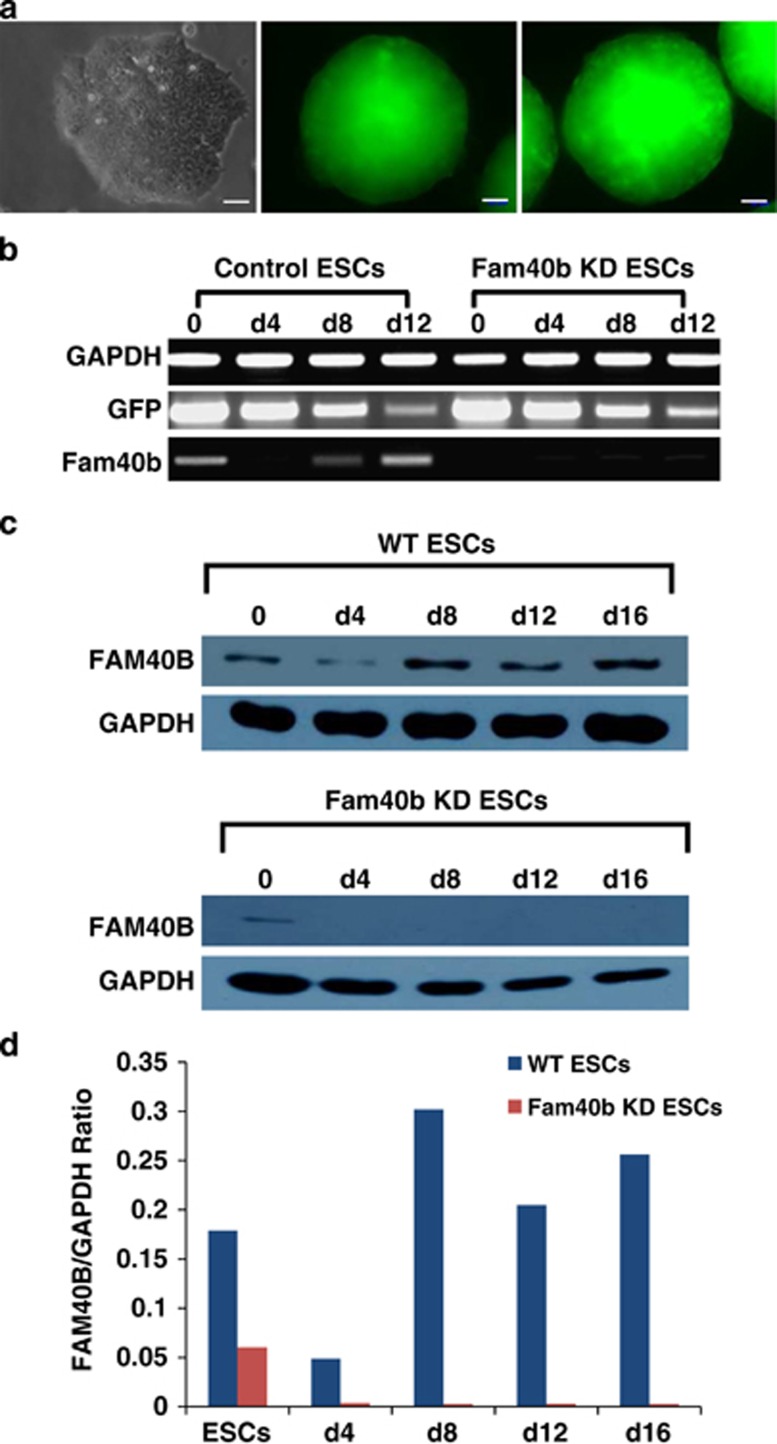
Generation and characterization of ESCs in which Fam40b was constitutively knocked down (KD) by transfection with pGFP-V-RS, expressing shRNA directed against the Fam40b RNA, the GFP and the puromycin resistance cassette. (**a**) Fluorescence microscopy of control 12-day EBs derived from ESCs transfected with the pGFP-V-RS vector without shRNA (control) and EBs derived from ESCs transfected with the pGFP-V-RS shRNA expressing vector containing 29-mers shRNA (Fam40b KD EBs) (scale bar: 50 *μ*m). (**b**) Semiquantitative RT-PCR analysis of the expression of GFP and Fam40b in undifferentiated control and KD Fam40bESCs as well as in 4-, 8- and 12-day control and KD EBs generated by the hanging drop protocol. (**c**) Expression of the FAM40B protein during differentiation of WT ESCs (upper panel) and during differentiation of Fam40B KD ESCs. Detection has been performed with FAM40b primary antibody (sc-162799, Santa Cruz Biotechnology; 1 : 500 dilution) and with the secondary donkey anti-Goat IgG antibody (1 : 10 000 dilution). GAPDH has been detected using the anti-GAPDH antibody (1 : 25 000 dilution). (**d**) Densitometric analysis of the FAM40B and the corresponding GAPDH bands densities has been performed by using the ImageJ 1.47v software (National Institutes of Health, Bethesda, MD, USA) and the ratio of the FAM40B/GAPDH band densities has been blotted for the different time points of differentiation

Fam40b was knocked down in ESCs by short hairpin RNA (shRNA) using the pGFP-V-RS shRNA vector. Expression of truncated green fluorescence protein (tGFP) and a puromycin resistance cassette allow for monitor transfection and selection of transfected transgenic cells (Figure 1a). We generated a set of ESCs clones by transfecting the shRNA construct (TR508344A, Origene Technologies, Rockville, MD, USA) by electroporation and selection of stable clones. Only clones with strong GFP expression were selected for the study. The TR508344A plasmid containing the shRNA sequence 5′-GCAAGACACTAAGGAATGGCTGGAGTTGG-3′ targeting Fam40b mRNA nucleotide positions between 365 and 393 were further used for differentiation of ESCs to embryoid bodies (EBs). Application of the hanging drop protocol resulted in spontaneous differentiation of ESCs toward different somatic cells including cardiomyocytes.^14, 15, 16^ Fam40b knockdown ESCs (KD ESCs) maintained their pluripotent nature and showed no differences in morphology when compared with the ESCs transfected with the control vector (without the shRNA oligonucleotide) or wild-type (WT) ESCs. EBs generated from KD ESCs will be referred to as KD EBs. Control EBs were generated from ESCs stably transfected with the empty pGFP-V-RS vector. As shown in Figures 1b and c, expression of the tGFP continued until day 12 of differentiation. The expression level of Fam40b was reduced in 4-day control EBs and increased in 8- and 12-day EBs, whereas no significant expression was observed in undifferentiated Fam40b KD ESCs and the Fam40b KD EBs. To determine whether knockdown of *Fam40b* results in a reduced protein level, we checked the FAM40b expression levels using a Fam40b antibody (sc-162799; Santa Cruz Biotechnology, Inc., Dallas, TX, USA) by western blot. As indicated in Figure 1c, the gene expression level during differentiation correlated well with the protein expression level.

### Analysis of the differentially expressed genes

**Figure 2:**
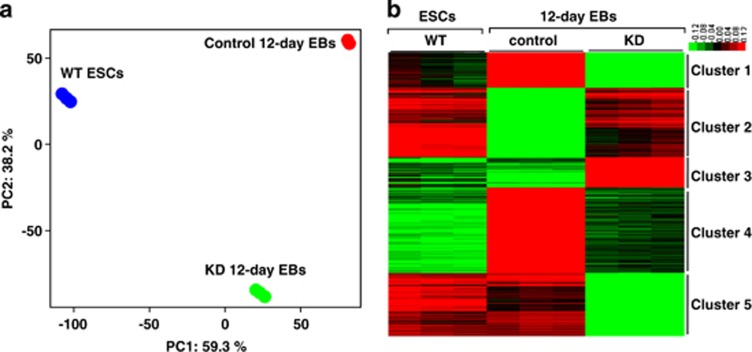
Transcriptome analysis of wild-type ESCs, control 12-day EBs and Fam40B KD 12-day EBs. (**a**) Principal component analysis of genome-wide gene expression. Each sphere represents an individual sample from a color-coded triplicate sample. (**b**) Visualization of *k*-means clustering of 5574 differentially expressed probe sets with Euclidean distance measurement and *k*=5 group clusters. Replicates are displayed in the vertical axis and genes in the horizontal axis. Log2 transformed signal intensities are depicted in color code. The heatmap indicates high expression levels in red, intermediate expression level in dark gray and low expression levels in green

To determine whether Fam40b levels affect the expression of genes participating in differentiation/developmental processes, the transcriptomes of both control and 12-day KD EBs were profiled and compared with the transcriptome of undifferentiated WT ESCs. Significance of variances of the expression levels were observed by principal component analysis (PCA). The percentage of variance at principal component (PC) 1 shows the highest variance in transcriptome variability among the biological samples and PC2 the second highest, respectively. As shown in Figure 2a, there are relatively large differences of the transcriptomes of all three cell populations. Interestingly, the transcriptome of the KD 12-day EBs was closer to undifferentiated WT ESCs in PC1 (corresponding to 60% of the variance) than the control 12-day EBs. Supplementary Table S1 shows the differentially expressed transcripts (6054 transcripts) between the control 12-day *versus* 12-day Fam40B KD EBs (2133 transcripts at least twofold upregulated and 3921 transcripts twofold downregulated, *P*<0.05). As indicated, several genes including cardiomyocyte-specific genes were remarkably downregulated in the KD EBs (e.g., *Tnnt2 and* *α*-*myosin heavy chain* *(Myh6)* more than 300-fold downregulated). Specific transcriptome clusters of the different cell populations were identified using *k*-means clustering algorithm over the transcripts significantly deregulated among different cell types at least twofold (Figure 2b).

**Figure 3:**
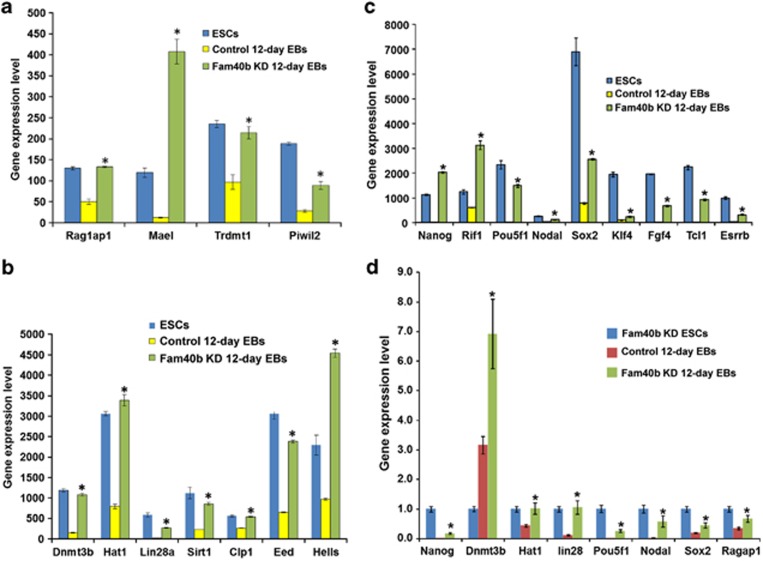
Expression levels of the genes belonging to the ‘GO:0040029∼regulation of gene expression, epigenetic' (**a** and **b**) and the expression of GO:0019827∼stem cell maintenance (**c**) pluripotent marker genes that are highly upregulated in the Fam40b KD 12-day EBs compared with the control 12-day EBs. (**d**) Gene expression of representative genes from (**a**–**c**) determined by qPCR analysis. The gene expression data of triplicates for each experimental condition are expressed as mean±S.D. (*P<0.05 for KD Fam40b 12-day *versus* control 12-day EBs)

The main representative developmental/differentiation-associated (BP) GOs and KEGG pathways identified by the annotation enrichment analysis for all the five clusters are shown in Table 1. Cluster 1 includes mainly genes that are weakly expressed in KD 12-day EBs as compared with the control 12-day EBs and ESCs and are clearly associated with developmental/differentiation processes such as heart, vascular and central nervous system development (Table 1). Moreover, we have identified several genes in clusters 2 and 3 with a high expression level in the KD 12-day EBs (red color); with a moderate expression level still in the WT ESCs but clearly with a low expression level in the control 12-day EBs (green color). GO analysis of the strongly upregulated genes in clusters 2 and 3 resulted in the identification of ncRNA metabolic processes GOs (see Table 1). Notably, cluster 3 transcripts demonstrated higher expression levels in KD 12-day EBs than in WT ESCs. Interestingly, cluster 2 includes several pluripotency-associated genes (*Nanog, Rif1, Esrrb, Pou5f1, Nodal, Sox2, Piwil2, Klf4, Fgf4, Tcl1*) with a high expression level, as expected in WT ESCs (red color) but also in KD 12-day EBs (Figure 2, cluster 2, Table 1, GO:0019827∼stem cell maintenance). As expected, the expression level of the pluripotency-associated genes should be low in 12-day EBs because of progressive differentiation after 12 days of differentiation. A similar expression pattern was observed for genes regulating gene expression via epigenetic mechanisms (Figure 2, cluster 2, Table 1, GO:0040029∼regulation of gene expression, epigenetic). Figure 3 shows the expression level of the epigenetic (Figures 3a and b) and pluripotency genes (Figure 3c) in all three cell populations. Moreover, we validated the gene array expression data with quantitative PCR (qPCR) methodology and examined the expression of representative genes from all three interesting GOs (Figure 3d). Cluster 4 has been identified as the largest cluster containing transcripts that show high expression levels in control 12-day EBs as compared with the WT ESCs and KD 12-day EBs (Table 1). As expected, because of the progressive differentiation, the control ESCs are capable of differentiating into various germ layer derivatives such as cardiac and neuronal cells. Besides mesodermal transcripts, ectodermal and to a lesser extent endodermal transcripts as well as transcripts participating in the KEGG signaling pathways such as the Wnt receptor and the transforming growth factor-*β* (TGF-*β*) receptor signaling pathway were expressed lower in the KD 12-day EBs as compared with the control 12-day EBs (Table 1). Cluster 5 GOs and KEGG signaling pathways include transcripts associated with developmental processes that were exclusively downregulated in the KD 12-day EBs.

### Transcriptomic analysis of Fam40b KD *versus* Scr and WT 12-day EBs

**Figure 4:**
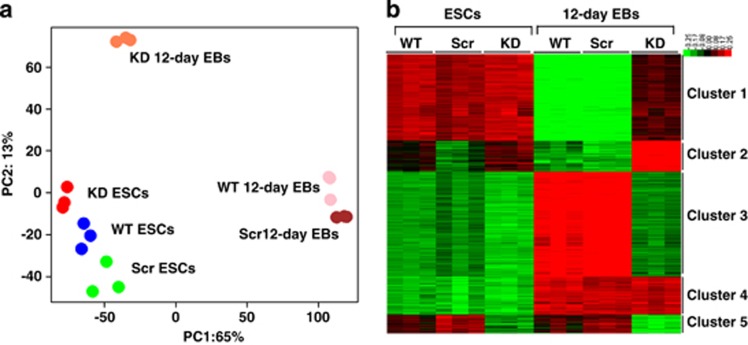
Global transcriptome analysis of WT ESCs, Scr ESCs, KD ESCs, WT 12-day, Scr 12-day and KD 12-day EBs. (**a**) Principal component analysis of genome-wide gene expression. Each sphere represents individual sample from a color-coded triplicate sample. (**b**) Visualization of *k*-means clustering of 5574 differentially expressed probe sets with Euclidean distance measurement and *k*=5 group clusters. Replicates are displayed in the vertical axis and genes in the horizontal axis. Log2 transformed signal intensities are depicted in color code. The heatmap indicates high expression levels in red, intermediate expression level in dark gray and low expression levels in green

**Figure 5:**
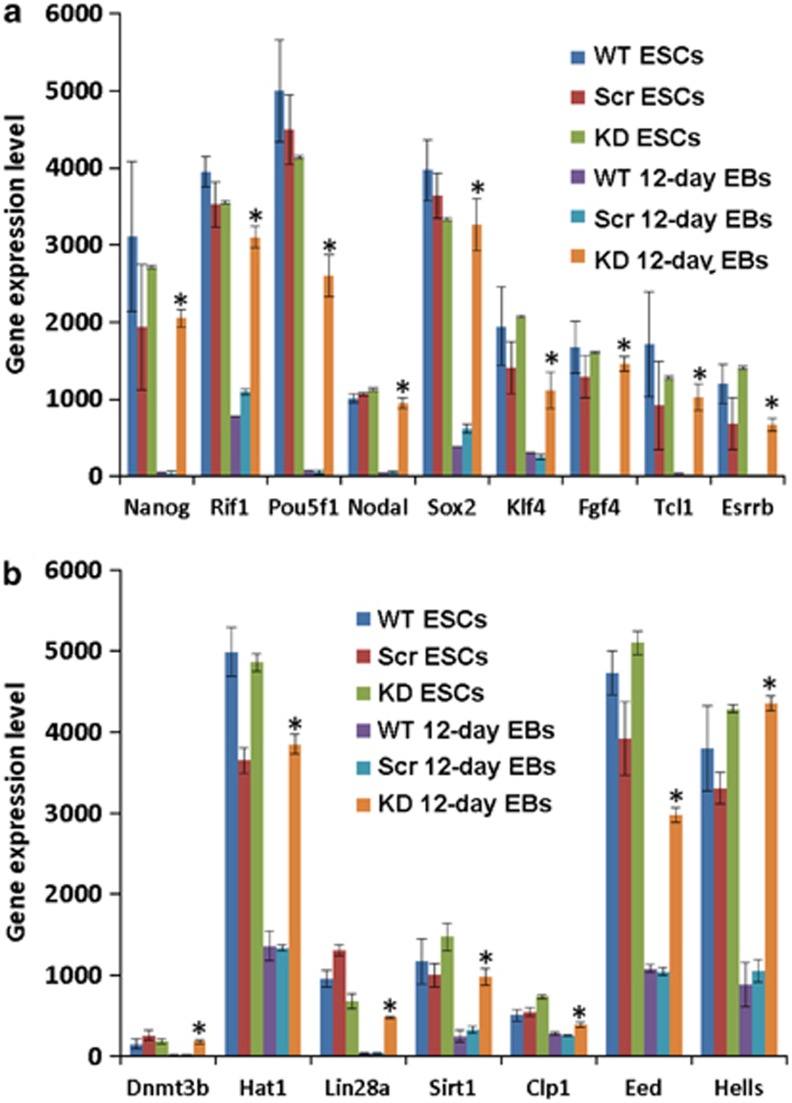
Expression levels of the genes belonging to the GO:0019827∼stem cell maintenance (**a**) pluripotency marker genes and ‘GO:0040029∼regulation of gene expression, epigenetic' (**b**) that are highly upregulated in the Fam40b KD 12-day compared with the Scr 12-day EBs. The gene expression data of triplicates for each experimental condition are expressed as mean±S.D. (*P<0.05 for KD Fam40b 12-day *versus* Scr 12-day EBs)

To give stronger evidence for the novel function of the Fam40b as a regulator of differentiation processes, we also generated scrambled ESCs (will now be referred to as Scr ESCs) applying the pGFP-V-RS shRNA approach and using the TR30013 containing the 29-mer scrambled shRNA oligonucleotide. The transcriptomes of WT ESCs, Scr ESCs as well as the transcriptomes of the Scr and KD 12-day EBs were then profiled and compared. In general, findings obtained by the comparison between control and KD 12-day EBs were confirmed by the comparison between Scr and WT *versus* Fam40b KD at 12-day post differentiation EBs. As shown in Figure 4a, there are relatively large differences between Scr 12-day and the KD 12-day EBs. Again, the transcriptome of the KD EBs was closer to undifferentiated WT, Scr and KD ESCs (all grouped together) than to the WT and Scr 12-day EBs (both grouped together). Compared with the transcriptome of the undifferentiated ESCs (WT, Scr and KD ESCs), the transcriptome of the KD 12-day EBs differed only in PC2 direction with a variance of <10%, whereas differences in PC2 were marginal. The *k*-means clustering shows the five clusters containing genes with a similar expression pattern (Figure 4b). Transcripts of the different five clusters and their representative annotation enrichment analysis are shown in Table 2. Cluster 1 transcripts indicated a high expression level in the WT, Scr and KD ESCs and in KD 12-day EBs but with a low expression level in the WT and Scr 12-day EBs. Again, genes associated with ncRNA processing, stem cell maintenance and epigenetic genes were identified to be highly upregulated not only in the WT, Scr and KD ESCs, but also in KD 12-day EBs as compared with the WT and Scr 12-day EBs (Table 2). Figure 5 shows the high expression level of pluripotent genes (Figure 5a) and epigenetic genes regulating gene expression (Figure 5b) in the different cell populations. In cluster 2, primary metabolic processes associated with lipid metabolisms and some GOs involved in developmental processes (e.g., vessel development) were identified to be upregulated in the KD 12-day EBs as compared with the WT, Scr and 12-day EBs. Cluster 3 has been identified as the largest cluster containing transcripts that show low expression levels in WT, Scr and KD ESCs and also in KD 12-day EBs as compared with WT and Scr 12-day EBs. As expected, progressive differentiation of the WT and Scr ESCs was accompanied by highly enriched developmental GOs and KEGG pathways in the WTs and Scr 12-day EBs as compared with the KD 12-day EBs (Table 2). Cluster 4 genes show upregulation of genes in all 12-day EBs populations in comparison with the undifferentiated ESCs populations. These genes belong to developmental processes and signaling pathways and are apparently not affected by knockdown of Fam40b. The expression of genes in cluster 5 are slightly decreased in the KD 12-day EBs as compared with WT and Scr 12-day EBs. These genes mainly belong to developmental processes such as heart development and blood vessel development.

### Cellular detection of Fam40b in undifferentiated ESCs

**Figure 6:**
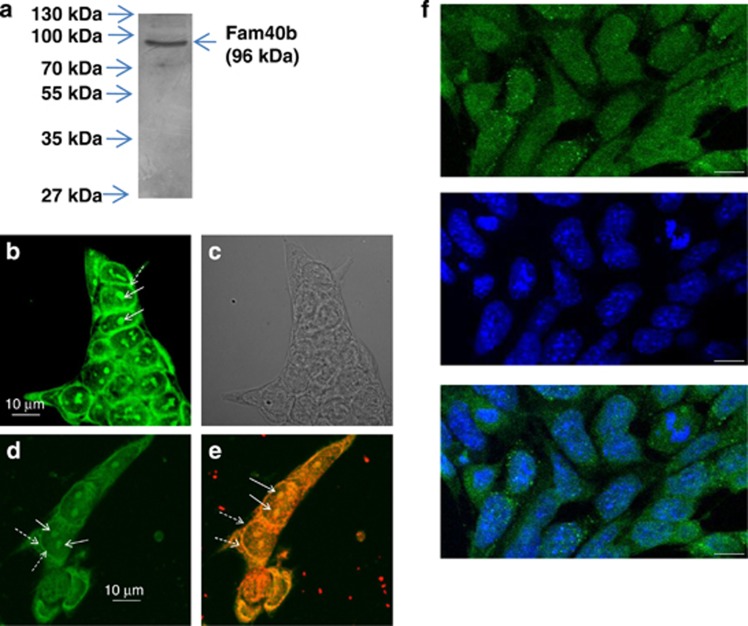
Molecular weight and cellular localization of FAM40B protein. (**a**) Protein lysates were prepared from undifferentiated ESCs. After separation of 40 *μ*g protein by SDS polyacrylamide (10%) gel electrophoresis (SDS-PAGE), western blotting of the proteins was done on nitrocellulose membrane. Chemiluminescence detection of FAM40B has been performed using the Fam40b-433–450 polyclonal antibodies and anti-Mouse IgG alkaline phosphatase-conjugated secondary antibodies. (**b** and **d**) Localization of FAM40B in ESCs. ESCs were transfected with the HaloTag Flexi Vector containing the Fam40b cDNA using TurboFect. After 48 h, Fam40b was detected using the HaloTag Oregon Green ligand in the nucleoli by confocal microscopy. Normal arrows show Fam40b in the nucleoli and dashed arrows the perinuclear Fam40b. (**c**) The transparent light microscopy of (**b**). (**e**) After fixing of the ESCs (in **d**), Fam40b has also been detected by immunohistochemistry using primary Anti HaloTag pAb (1 : 500 dilution) and anti-mouse IgG Alexa Fluor 594 secondary antibodies. (**f**) Immunostaining of Fam40b in WT ESCs using primary anti-Fam40b antibodies (sc-162799; 1 : 200) and donkey anti goat IgG-FITC secondary antibody (sc-2024, 1 : 200) as secondary antibody (upper scan, green pseudocolor). Cells were co-stained with the nuclear marker Hoechst 33342 (scan in the middle, blue). The overlay of nuclear and Fam40b staining (**f**, bottom) reveals that the presence of Fam40b is not restricted to the nucleus but also extends to perinuclear or even cytoplasmic domains of the ESCs (scale bar: 10 *μ*m)

According to the Cell-PLoc 2.0 bioinformatics tool for predicting subcellular localization of proteins in different organisms,^17^ Fam40b encodes a protein of 844 amino acids (96 kDa) with predicted cytosolic location. To verify this prediction model, polyclonal antibodies were raised against Fam40b-433–450 (KVRQKDIEHFLEMSRNKF). In western blots of cellular extracts isolated from undifferentiated control ESCs, this antiserum recognized a protein with a molecular weight of 96 kDa (Figure 6a). To determine the localization of the protein we generated a fusion protein consisting of the HaloTag protein and Fam40b. After transient transfection of undifferentiated ESCs with the Fam40b-HaloTag construct, the fusion protein was detected using the Oregon Green ligand (Promega, Madison, WI, USA). Moreover, the protein was also detected by immunocytochemistry using HaloTag-specific antibodies. As indicated in Figures 6b, d and e, FAM40B was detected in the perinuclear region and interestingly also in the nucleoli. The transparent light microscopy of Figure 6b is shown in Figure 6c. In an effort to determine the localization of the FAM40B in the ESCs, we applied immunostaining using the recently available primary Fam40b antibodies (Santa Cruz Biotechnology). As indicated in Figure 6f (lower panel), the presence of Fam40b is not restricted only to the nucleus but also extends to the perinuclear and cytoplasmic domains of the ESCs.

### Fam40b KD ESCs fail to differentiate into beating cardiomyocytes

Perturbation of cardiomyocyte differentiation processes of ESCs can be monitored by examining the increasing beating activity of EBs during differentiation of ESCs to cardiomyocytes. Therefore, to determine whether Fam40b is essential for differentiation of ESCs toward different cell types, we differentiated cells in ‘hanging drops' using KD ESCs in comparison with the Scr ESCs and WT ESCs. On days 8, 10, 12 and 16 of differentiation, the number of beating EBs was counted from 50 EBs performed (=100%). No cardiomyocyte beating activity was observed after differentiation of the KD ESCs (Supplementary Figure S1). Representatively, the beating activity of the control and KD 12-day EBs is shown in the video recordings in the Supplementary Movies M1 and M2, respectively. The control and Scr ESCs were able to differentiate into functional beating cardiomyocytes, in contrast to KD ESCs. These data show that Fam40b knockdown can cause a complete loss of differentiation potential toward functional cardiomyocytes.

### Fam40b knockdown in zebrafish causes heart defects

**Figure 7:**
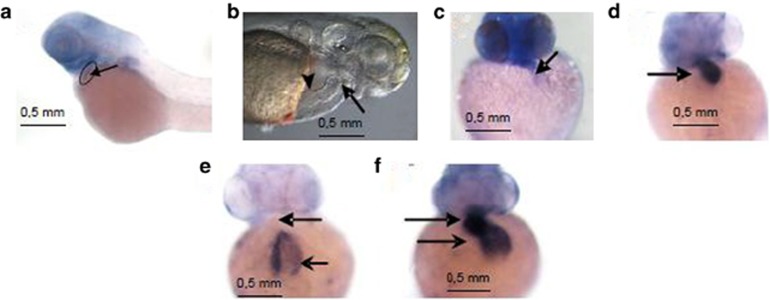
The *in situ hybridization* of Fam40b in the zebrafish heart as well as the expression of *vmhc* and of cardiac *cmlc2* in control and Fam40b knockdown animals. (**a**) Digoxigenin-labeled RNA probes were prepared using RNA labeling kit and stained using BM purple. (**b**) The position of the atrium (arrowhead) and the ventricle (arrow) that was not beating after knockdown of Fam40b is shown. (**c**) Expression of *vmhc* in Fam40b knockdown animals was significantly impaired as compared with control animals (**d**) (arrows show the ventricles). Expression of *cmlc2* in Fam40b knockdown animals was almost absent in ventricles and impaired in the atria (**e**) as compared with control (**f**) (upper arrows indicate ventricles and lower arrows indicate atria; one representative experiment out of five independent). Whole mount embryos were imaged on a Leica stereomicroscope fitted with a Zeiss Axiocam color camera

To validate the *in vitro* studies suggesting an important role of Fam40 for differentiation/developmental processes including cardiomyogenesis under *in vivo* conditions, we knocked down Fam40b in zebrafish. Two morpholino oligonucleotides (MOs) directed against Fam40b and one control MO were injected into one- to two-cell-stage embryos of the *TG(fli:EGFP)* zebrafish line.^18^ To monitor a potential perturbation in the expression of cardiomyocyte-specific markers, we looked at the expression levels of cardiac *vmhc* and of cardiac *cmlc2* in 2-day MO1 Fam40b knockdown embryos. As shown, the Fam40b morphants exhibit impaired expression of *vmhc* and *cmlc2* as compared with control animals (Figure 7). Knockdown of Fam40b resulted in heart defects observable 48 h post injection. The most prominent effects were ventricle stagnancy, enlarged atrium and vascular defects. Ventricular stagnancy was observed in 54% (42 out of 78 embryos) of morpholino I (MO1)-treated embryos and in 51% (57 out of 111) of morpholino II (MO2)-treated embryos (Figure 7b). In addition, vascular defects were observed in 23% and 35% of the MO1 and MO2 morphants, respectively. In contrast, none of the control MO-injected embryos displayed any of these defects.

## Discussion

Until now, the function of the novel transcript Fam40b was unknown. However, more recently there is increasing evidence that Fam40b is a part of the STRIPAK complex and therefore may be involved in the regulation of cell differentiation and cardiac function.^10, 11, 12^ To elucidate the role of Fam40b for cell differentiation, we silenced Fam40b in ESCs and investigated its role for the differentiation of ESCs. We were able to demonstrate that Fam40b encodes for a protein with a molecular weight of 96 kDa that is also located perinuclearly in cytosol and in the nucleoli of the ESCs. Knockdown of the Fam40b compromises the differentiation of mouse ESCs including cardiomyocytes. Key transcription factors required for maintaining pluripotency of stem cells include Pou5f1, Nanog and Sox2 (for review see Niwa^19^). These molecules are vital for maintaining the identity of the ICM during mouse preimplantation development.^20, 21, 22, 23^ In additional to transcriptional regulation, epigenetic modifications, miRNAs and the cell-to-cell communications also participate in the maintenance of pluripotency and differentiation.

We identified several additional genes such as *Pou5f1* (also known as *Oct4*) and *Nanog* participating into the pluripotency of ESCs as well as genes participating in the epigenetic regulation of the gene expression such as *Hat1*, *Lin28a* and *Dnmt3b* that are upregulated in the undifferentiated ESCs and in Fam40b KD 12-day EBs but downregulated in control 12-day EBs. In general, epigenetic regulation of gene expression occurs mainly via DNA methylation and/or histone deacetylation, both of which induced suppression of the gene expression. Gene silencing via hypermethylation of mainly CpG dinucleotides involves enzymatic methylation by DNA methyltransferases of cytosine residues. Approximately 60 to 70% of gene promoter regions overlap with CpG islands. Gene silencing by hypermethylation of the CpG islands plays an important role in the switch between the pluripotent and differentiation state of ESCs.^24^ There are two types of DNA methyltransferases: DNMT1 is a ‘maintenance' DNA methyltransferase and is responsible for methylating cytosines at hemimethylated CpG sites. DNMT3A and DNMT3B are ‘*de novo*' DNA methyltransferases and methylate completely unmethylated loci. During early embryonic development, Dnmt3a and Dnmt3b are expressed at high levels and apparently define the normal embryonic methylation pattern.^25, 26^ In ESCs, DNMT3A and DNMT3B methylate *Pou5f1* and *Nanog* promoters during differentiation. It has been established that the DNA methylation *in vivo* and in ESCs is indeed critical for early developmental processes, but is required for differentiation rather than for maintenance of the pluripotent state.^24, 27, 28, 29^ *HAT1* is significantly upregulated in Fam40b KD 12-day EBs, suggesting a possible role of Fam40b in the *Hat1*-associated epigenetic modulations. Recently, there is increasing evidence that Hat1 plays an important epigenetic role in the chromatin assembly of both nonmammalian and mammalian cells (for review see Parthun^30^). Epigenetic mechanisms including chromatin rearrangements by DNA methylation and histone modifications are implicated in mammalian development and are characteristic of differentiation of ESCs toward somatic cells.^31^ *Hat1* is overexpressed in adult stem cells such as hematopoietic stem cells (HSCs)^32^ and neural stem cells.^33^ Interestingly, more recently, LIN28 has been identified as a pluropotency factor in mature nucleolus in mouse preimplantation embryos and also in ESCs where it colocalizes with nucleophosmin 1.^34^

Our findings suggest that Fam40b during development suppresses the expression of genes associated with RNA metabolic processes, as is indicated by our observation of an upregulation of RNA metabolic processes, including ncRNA processing, upon knockdown of Fam40. Although the role of ncRNA for the regulation of various biological processes remains unclear, more recently, some ncRNAs have attracted a great deal of attention because they seem to regulate gene expression via epigenetic regulation thereby, regulating embryogenesis (for review see Pauli *et al.*^35^). The ncRNAs include the so-called ‘housekeeping' ncRNAs (ribosomal RNA, transfer RNA, small nuclear RNA and small nucleolar RNA), regulatory ncRNAs such as microRNA (miRNAs), endogenous siRNAs (endo-siRNAs) PiWI-interacting RNAs (piRNAs), long noncoding RNA (lncRNA) and several poorly characterized ncRNAs originating from regulatory elements.^35^

These results suggest that Fam40b, which has also been identified in the nucleoli of ESCs, might be involved in the regulation of ribosomal RNA (rRNA) expression and in the expression of other ncRNAs, thereby regulating the differentiation processes of ESCs. In the nucleolus, different processes for ribosomal biogenesis occur such as transcription of rDNA to rRNA and maturation of rRNAs that further assemble with ribosomal proteins. Thereafter, intact ribosomes can be released from the nucleoli to the cytosol to initiate the translation process (for review see Hernandez-Verdun *et al.*^36^). Therefore, the size and the organization of the nucleoli, which defines the number, and the maturation of the ribosomes are associated with cellular processes such as proliferation and differentiation. For instance, the size of the nucleolus represents a diagnostic marker for the proliferative potential of cancer cells.^36, 37^ We therefore suggest that Fam40b may interact with the ribosomal machinery, thereby participating in the control of the ESC differentiation status. Apparently, because of the absence of Fam40b, a perturbation of the ESC differentiation processes occurred including differentiation to cardiomyocytes.

More recently, it has been demonstrated that Fam40b is located in the cytosol and is involved in the regulation of cell morphology of HeLa and PC3 prostate cancer cells.^11^ FAM40B-depleted HeLa or FAM40B-depleted PC3 cells appeared to detach from each other or to reduce migration, respectively.^10, 11, 12^ FAM40B depletion in PC3 cancer cells increased elongation of the cells, suggesting a potential role in the regulation of cytoskeletal organization and cell morphology.^10, 11, 12^ It has lately been recognized that the cytoskeletal organization is different in undifferentiated ESCs. It has been demonstrated that the pluripotency of ESCs is associated with disorganized perinuclear actin cytoskeleton, whereas progressive differentiation of ESCs is associated with an organized actin cytoskeleton.^38, 39^ Another regulator of the cytoskeleton involved in differentiation processes is Sirt2. Recently, it has been shown that knockdown of Sirt2 by the shRNA approach resulted in promotion of mesodermal and endodermal cells while inhibiting ectodermal cells.^39^ In summary, we may conclude that the novel gene Fam40b is essential for early and late lineage commitment of mESCs via mechanisms involving pluripotency and epigenetic networks.

## Materials and Methods

### Culturing and differentiation assays of ESCs

The loss-of-function experiments were performed with murine CGR8 ESC (European Collection of Cell Cultures (ECACC) No. 95011018). The cells were maintained on (0.2%) gelatinized tissue-culture dishes in feeder-free conditions in a standard ES culture medium consisting of Glasgow's minimum essential medium (GMEM, Invitrogen GmbH, Karlsruhe, Germany) supplemented with 10% fetal bovine serum (FBS, GIBCO, Grand Island, NY, USA), 2 mmol/l L-glutamine, 100 units/ml leukemia inhibitory factor (LIF-1, Calbiochem, San Diego, CA, USA) and 50 *μ*mol/l *β*-mercaptoethanol (Invitrogen GmbH) as described previously.^40^ The cells were passaged on alternate days and maintained confluence between 60 and 70%. ESC differentiation was inducted by the conventional ‘hanging drop' protocol, as described previously.^40^ Briefly, 20 *μ*l hanging drops were made in a 10 cm low adhesion dishes from trypsin-dissociated ESC suspension (2.5 × 10^4^ cells/ml) prepared in differentiation medium (Iscove's modified Dulbecco's medium (IMDM; Life Technologies, Darmstadt, Germany) supplemented with 20% fetal calf serum, 1% nonessential amino acids, 2 mM L-glutamine and 100 *μ*M *β*-ME. Plates were incubated at 37 °C, 5% CO_2_ in a humidified incubator for 2 days. EBs thus formed were harvested by washing and resuspended in differentiation medium. The EBs were incubated at 37 °C in 5% in an CO_2_ incubator under shaking conditions, with a medium change every alternate days. EBs were monitored for beating areas called cardiomyocyte foci starting from day 7 after differentiation on an inverted phase contrast microscope (Zeiss Axiovert25, Oberkochen, Germany). The numbers of beating EBs were counted and representative videos were captured (Sony DFW-X700, Sony Corporation, Tokyo, Japan).

### Transfection of vectors into undifferentiated ESCs and generation of a constitutive Fam40b KD ESC line

The shRNA expression vector pGFP-V-RS targeting mouse Fam40b (TR508344A plasmid) and the scrabbled plasmid (TR30013) were purchased from OriGene (Rockville, MD, USA). The shFam40b target sequences on its mRNA were 5′-GCAAGACACTAAGGAATGGCTGGAGTTGG-3′ that corresponds to nucleotides 365–393. The scrambled plasmid (TR30013) contains a nonactive scrambled sequence cassette (5′-GCACTACCAGAGCTAACTCAGATAGTACT-3′). pGFP-V-RS control, the shRNA vector (TR508344A plasmid) and the scr shRNA vector (TR30013) were linearized. Using Bio-Rad Gene Pulser (Hercules, CA, USA) electroporator, 25 *μ*g of linearized vector was transfected into 10^6^ ESCs suspended in phosphate-buffered saline (PBS) free of Ca^2+^ and Mg^2+^ salts. The electroporation conditions were 500 *μ*F and 240 V as described previously.^41^ The electroporated cells were cultivated on gelatinized tissue culture flasks for 2 days and eventually selected by treatment with 2 *μ*g/ml puromycin. On day 10 of selection, green fluorescence was monitored under blue excitation light through a fluorescence microscope, after which the resistant clones were picked for further experiments and amplified following standard ESC culture conditions. The clones were passaged at least four times before use in the experiments to attain a stable gene expression profile. The shFAM40b cell line generated with the TR508344A plasmid was used to study gene expression changes during differentiation by forming EBs.

### Microarray analysis

Total RNA was isolated from the Fam40b KD 12-day EBs, control 12-day EBs and WT undifferentiated ESCs using RNeasy mini kit (Qiagen, Hilden, Germany). Then, 100 ng total RNA was used for aRNA amplification with GeneChip 3′ IVT Express Kit (Affymetrix, Santa Clara, CA, USA). After 16 h of biotinylated *in vitro* transcription, amplified RNA (aRNA) was purified and 15 *μ*g of purified aRNA was fragmented with fragmentation buffer. Next, 12.5 *μ*g of fragmented aRNA was hybridized with Mouse Genome 430 2.0 arrays (Affymetrix) for 16 h at 45 °C. Arrays were washed and stained with phycoerythrin with Affymetrix Fluidics Station 450 and scanned using the Affymetrix Gene-Chip Scanner 3000 7G (Affymetrix). The quality control matrices were confirmed with Affymetrix GCOS software. The raw data were background corrected, summarized and normalized using RMA algorithm executed by R bioconductor packages.^42^ A PCA was performed to observe the samples transcriptome variability. Significantly regulated transcripts were determined by empirical Bayes linear model applied using the LIMMA package in R.^43^ The significance of the change was calculated correcting the *P-*value of the *t*-score with false discovery rate using Benjamini–Hochberg method at *P*≤0.05 and the size of change was calculated with a twofold regulation. Further on significantly expressed probe sets, *k*-mean cluster analysis was performed after transcript-wise normalization of signal values to a mean of 0 and S.D. of 1 using Euclidian distance measurement and *k*=5, using Cluster 3.0.^44^

### Gene ontology and pathway analysis

To determine the biological significance of differentially expressed transcripts (DETs), the Database for Annotation, Visualization and Integrated Discovery (DAVID) was used (http://david.abcc.ncifcrf.gov/). DAVID provides biological process, molecular function and cellular component for DET with EASE enrichment score at *P*≤0.01.^45, 46^

### RNA isolation and reverse transcriptase-PCR (RT-PCR)

The total RNA was isolated using an RNeasy kit (Qiagen) with on-column DNase I (Qiagen) digestion according to the manufacturer's instructions. Then, 2 *μ*g of total RNA were reverse-transcribed with SuperScript Vilo (Invitrogen GmbH) with random hexamers according to the manufacturer's recommended protocol. The endogenous mRNA levels for each gene were measured by PCR. The cDNA pool was subjected to semiquantitative PCR amplification (denaturation at 95 °C/2 min, 22–35 cycles of 95 °C/30 s denaturation, 60 °C /30 s annealing 72 °C/60 s of elongation) using DreamTaq Polymerase (Thermo Fisher Scientific, Pittsburgh, PA, USA) keeping 0.4 *μ*M concentration of each primer. The PCR products were resolved in 2% agarose gels with 0.001% ethidium bromide. The primer pairs included in this study are listed in the Supplementary Table S2. Real-time qPCR was carried out on an ABI-7500 Fast PCR system (Applied Biosystems, Foster City, CA, USA) using SYBR Green PCR master mix (MESA FAST mix, Eurogentec, Brussels, Belgium). The following PCR standard condition were used: 95 °C/5 min taq activation, 40 cycles of 95 °C/15 s, 60 °C/30 s and 72 °C/30 s. The samples were run in duplicate, and the experiments were repeated at least three times. The mRNA levels were normalized against endogenous control (*β*-actin) levels and calculated using the cycle threshold (Ct) method. The primers used in real-time PCR analysis are summarized in Supplementary Table S2.

### Western blotting

Equal quantities (40 *μ*g) of protein lysate prepared in sodium dodecyl sulfate (SDS) cell lytic buffer (Thermo Fisher Scientific) were resolved in denaturing (10%) polyacrylamide gels. Separated proteins by SDS polyacrylamide gel electrophoresis (SDS-PAGE) were transferred to Immobilon-P or nitrocellulose membranes at 4 °C for 2 h at 250 mA, membranes were blocked for 1 h in 5% non-fat milk/Tris-buffered saline and Tween-20 (TBST) buffer, incubated with primary antibody overnight, washed three times for 10 min, incubated in secondary antibody for 1 h, washed five times for 10 min and developed using enhanced chemiluminescence (ECL) detection system (Pierce Biotechnology, Inc., Rockford, IL, USA) on a photographic film (Kodak Biomax, Rochester, NY, USA).

Polyclonal antibodies against Fam40b were generated by Thermo Fisher Scientific by immunization of rabbits with the peptide Fam40b-433–450 (KVRQKDIEHFLEMSRNKF). The Fam40b polyclonal antibody (sc-162799) was purchased from Santa Cruz Biotechnology. Anti-*β*-actin (A2228) and anti-GAPDH antibodies were purchased from Sigma (Munich, Germany). Polyclonal anti-Rabbit IgG (SA5-10052) antibodies and anti-Mouse IgG alkaline phosphatase (A3562) were purchased from Thermo Fisher Scientific and Sigma. Donkey anti-Goat IgG secondary antibody (926–32214) was obtained from LI-COR Biosciences (Lincoln, NE, USA).

### Immunohistochemistry

To visualize the localization of Fam40b, the ESCs were seeded at ∼2.5 × 10^4^ cells/cm^2^ on 0.1% gelatin-coated cover slips and cultured at 37 °C and 5% CO_2_ at 24 h before experiments. At a confluence of 70%, the cells were fixed with 4% paraformaldehyde (PFA) in PBS (10 mM sodium phosphate, 2.7 mM KCl, 140 mM NaCl, pH 7.4) for 15 min and then permeabilized with 0.4% Triton X-100 diluted in blocking buffer (5% BSA diluted in PBS) for 15 min at room temperature (RT). After washing the cells three times with PBS, 5 min each, and blocking for 45 min at RT, the cells were incubated with Fam40b antibody (sc-162799; Santa Cruz Biotechnology) diluted 1 : 200 in blocking buffer for 1 h at RT. Excessive antibodies were washed off with PBS and cells were incubated with donkey anti goat IgG-FITC secondary antibody (sc-2024 Santa Cruz Biotechnology) diluted 1 : 200 in blocking buffer for further 1 h, followed by 3 washes with PBS, 5 min each. To visualize the nucleus the cells were incubated with Hoechst 33342 (Life Technologies, Carlsbad, CA, USA) diluted (1 : 1000) in blocking buffer for 1 h. After the final wash, the cells were fixed on microscopic slides with PolyGlass cover slipping medium (Polysciences, Warrington, PA, USA). Images were captured with an inverted fluorescence microscope (Zeiss Axiovert 200) or, for higher subcellular resolution of nuclei and nucleoli, with the Olympus (Hamburg, Germany) FluoView1000 confocal system as described earlier.^47^

### Detection of Fam40b in ESCs via HaloTag

To detect the localization of the Fam40b protein we applied the HaloTag technology (Promega Corporation, Madison, WI, USA) after transient transfection of undifferentiated ESCs with pFN21K HaloTag CMV Flexi vector containing the Fam40b cDNA. The HaloTag reporter protein (∼33 kDa) is an engineered, catalytically inactive derivative of a hydrolase that forms a covalent bond with HaloTag ligands. ESCs (WT, 10^5^ cells) were plated and cultured in gelatinized six-well plates at a confluence of ∼50 to 70%. The cells were then transfected with a mixture of 1 *μ*g vector and 2 *μ*l TurboFect (Thermo Fisher Scientific) in 200 *μ*l DMEM. After 6 h of culturing, culture medium (4 ml) was replaced with fresh culture medium. After 48 h, FAM40B protein was detected using the HaloTag Oregon Green ligand by confocal fluorescence microscopy as described in the manual. In addition, the protein was also detected by immunocytochemistry after fixing the cells with 4% paraformaldehyde/0.2 M sucrose/1 × PBS using primary Anti HaloTag pAb and Alexa Fluor 594 anti-mouse IgG as a secondary antibody (1 : 500 dilution; Life Technologies).

### Morpholino design and microinjection in zebrafish embryo

WT and transgenic (fli1:EGFP, a kind gift from Dr. Gerd-Jörg Rauch, Heidelberg, Germany) zebrafish stocks were maintained at 28.5 °C at 14-h light/10-h dark cycle.^48^ Embryos were obtained from natural spawning and staged as previously described.^49^ Embryos were treated with 0.2 mM 1-phenyl-2-thiourea (PTU, Sigma) after 24 HPF to inhibit pigmentation. Morphological changes were determined under a stereomicroscope (Leica, Heerbrugge, Switzerland). Morpholino antisense oligonucleotides (MOs) were obtained from Gene Tools (Corvallis, OR, USA). To identify putative zebrafish orthologs, BLAST searches were performed using predicted peptide sequences (NCBI Unigene, http://www.ncbi.nlm.nih.gov/unigene) as a query for the Ensembl Zebrafish peptide database. The cDNA sequences of the transcripts of interest were obtained from NetAffx (Affymetrix). Respectively, two MOs were designed against zfFam40b and targeted to inhibit translation.^50^ Control injections to assess off-target effects were performed with a mismatch MO. Morpholino antisense oligonucleotide sequences are as follows: zfFam40b I- 5′-TAGCACATAAACCGACACCGTCCAT-3′ zfFam40b II- 5′ mismatch MO 5′-CCTCTTACCTCAGTTACAATTTATA-3′. The MOs were diluted 0.6 to 1.0 mM in H_2_O, 0.1 M KCl and 0.2% phenol red. Embryos were injected at the one- to two-cell stage using a Femtojet (Eppendorf, Hamburg, Germany) and fixed at appropriate time points. MO-injected embryos were compared with uninjected and mismatch MO (MMO)-injected controls from the same clutch.

### *In situ* hybridization

Whole mount *in situ* hybridization was performed as previously described^51^ using an automated InsituPro system (Abimed, Langenfeld, Germany).^52^ Digoxigenin-labeled RNA probes were prepared using an RNA labeling kit (Roche, Indianapolis, IN, USA) and stained using BM purple (Roche). Whole mount embryos were imaged on a Leica stereomicroscope fitted with a Zeiss Axiocam color camera. For generating *in situ* probes, following PCR primers were used (forward and reverse primers are flanked with T3 and T7 promoter sequences respectively): zfFam40b-Forw-T3–5′-AATTAACCCTCACTAAAGGGAATGATACAGATAC-3′ zfFam40b-Rev-T7–5′-TAATACGACTCACTATAGGGTTGCTCTGGTTCTT-3′ vmhc-Forw-T3–5′-AATTAACCCTCACTAAAGGGAGGAAAGAGCATCC-3′ vmhc-Rev-T7-5′-TAATACGACTCACTATAGGGCCCACATCACGACT-3′ amhc-Forw-T3–5′-AATTAACCCTCACTAAAGGGCGAGATATGAGACT-3′ amhc-Rev-T7-5′-TAATACGACTCACTATAGGGTTTCTTTCAAGCAT-3′ cmlc2-Forw-T3–5′-AATTAACCCTCACTAAAGGGATGGAGTTATCAAC-3′ cmlc2-Rev-T7-5′-TAATACGACTCACTATAGGGCAGCAGTTACAGAC-3′.

### Statistical analysis

If not indicated in the text, analysis was performed by one-way pairwise ANOVA test. The *P-*values of <0.05 are considered as statistically significant.

## Figures and Tables

**Table 1:** Developmental GO BPs as well as KEGG pathways differentially regulated in KD 12-day EBs in comparison with control 12-day EBs and WT ESCs

**Term**	**Transcripts**	***P*****-value**
*Cluster 1: GOs and KEGG pathways including highly upregulated genes in control 12-day and moderate expressed in WT ESCs but very low expressed in KD 12-day EBs*		
GO:0007507∼heart development	20	9.47E−05
GO:0045597∼positive regulation of cell differentiation	16	4.77E−04
GO:0030705∼cytoskeleton-dependent intracellular transport	7	5.37E−04
GO:0001568∼blood vessel development	19	8.25E−04
GO:0001944∼vasculature development	19	0.001092
GO:0001649∼osteoblast differentiation	7	0.003581
GO:0007417∼central nervous system development	23	0.00363
GO:0060348∼bone development	11	0.004406
GO:0007420∼brain development	19	0.005137
GO:0043009∼chordate embryonic development	24	0.008354
GO:0030324∼lung development	10	0.008945
mmu04310:Wnt signaling pathway	17	1.00E−05
mmu04810:Regulation of actin cytoskeleton	19	9.47E−05
mmu04340:Hedgehog signaling pathway	8	0.001149
mmu05414:Dilated cardiomyopathy	10	0.001831
mmu04360:Axon guidance	12	0.002053
		
*Cluster 2: GOs including upregulated genes in WT ESCs and KD 12-day EBs as compared with control 12-day EBs*		
GO:0034660∼ncRNA metabolic process	42	1.16E−13
GO:0034470∼ncRNA processing	34	1.34E−11
GO:0006396∼RNA processing	53	6.21E−08
GO:0019827∼stem cell maintenance	10	9.91E−07
GO:0040029∼regulation of gene expression, epigenetic	14	8.25E−05
		
		
*Cluster 3: GOs including upregulated genes KD 12-day EBs as compared with control 12-day EBs and WT ESCs*		
GO:0006396∼RNA processing	27	8.80E−06
GO:0016070∼RNA metabolic process	33	5.44E−05
GO:0000154∼rRNA modification	4	6.17E−04
GO:0034470∼ncRNA processing	12	0.001053
GO:0043414∼biopolymer methylation	8	0.001189
GO:0009451∼RNA modification	6	0.002623
GO:0034660∼ncRNA metabolic process	12	0.007034
		
*Cluster 4: GOs and KEGG pathways including genes that are upregulated in control 12-day EBs as compared with WT ESCs and KD 12-day EBs*		
GO:0007507∼heart development	54	1.98E−17
GO:0001568∼blood vessel development	56	6.00E−17
GO:0048732∼gland development	38	2.42E−09
GO:0060348∼bone development	28	4.45E−09
GO:0022008∼neurogenesis	71	6.88E−09
GO:0030323∼respiratory tube development	27	7.60E−09
GO:0001822∼kidney development	26	1.06E−08
GO:0051216∼cartilage development	20	3.07E−07
GO:0016055∼Wnt receptor signaling pathway	25	2.15E−06
GO:0048565∼gut development	13	3.38E−06
GO:0035108∼limb morphogenesis	22	1.10E−05
GO:0048666∼neuron development	39	2.18E−05
GO:0008016∼regulation of heart contraction	13	5.47E−05
GO:0007417∼central nervous system development	44	9.31E−05
GO:0007219∼Notch signaling pathway	13	1.22E−04
GO:0030111∼regulation of Wnt receptor signaling pathway	11	1.26E−04
GO:0045664∼regulation of neuron differentiation	18	2.37E−04
GO:0031016∼pancreas development	9	0.001984
GO:0048286∼lung alveolus development	7	0.0020672
GO:0030900∼forebrain development	22	0.0022447
GO:0007498∼mesoderm development	11	0.0038993
GO:0001889∼liver development	9	0.00531
GO:0048567∼ectodermal gut morphogenesis	5	0.0099191
mmu05414:Dilated cardiomyopathy	22	2.44E−07
mmu04350:TGF-*β* signaling pathway	17	1.15E−04
mmu04310:Wnt signaling pathway	21	0.0013417
		
*Cluster 5: GOs and KEGG pathways including genes with low expression level only in KD 12-day EBs as compared with the other cell population*		
GO:0007507∼heart development	24	0.001835
GO:0002076∼osteoblast development	5	0.001952
GO:0043414∼biopolymer methylation	11	0.004197
GO:0006396∼RNA processing	37	0.006022
mmu04910:Insulin signaling pathway	18	3.68E−04
mmu04810:Regulation of actin cytoskeleton	23	8.58E−04
mmu04310:Wnt signaling pathway	18	8.99E−04

GOs include genes that are at least twofold up- or downregulated

**Table 2:** Selected significantly regulated GO-BPs as well as KEGG pathways in KD 12-day EBs *versus* Scr 12-day EBs

**Term**	**Transcripts**	***P*-value**
*Cluster 1: GOs including genes indicated a high expression level in the WT, Scr and KD ESCs and in KD 12-day EBs but with a low expression level in the WT and Scr 12-day EBs*		
GO:0034660∼ncRNA metabolic process	74	1.95E−20
GO:0034470∼ncRNA processing	62	6.29E−19
GO:0006396∼RNA processing	109	2.77E−15
GO:0043414∼biopolymer methylation	25	5.71E−07
GO:0040029∼regulation of gene expression, epigenetic	22	2.35E−05
GO:0019827∼stem cell maintenance	11	6.81E−05
		
*Cluster 2: GOs including genes upregulated in the KD 12-day EBs as compared with the WT, Scr and 12-day EBs*		
GO:0016125∼sterol metabolic process	17	8.87E−08
GO:0006749∼glutathione metabolic process	7	3.66E−04
GO:0001568∼blood vessel development	23	5.78E−04
GO:0006631∼fatty acid metabolic process	19	7.00E−04
mmu00100:Steroid biosynthesis	6	6.18E−04
mmu00052:Galactose metabolism	7	8.99E−04
mmu03320:PPAR signaling pathway	11	0.002151043
mmu00051:Fructose and mannose metabolism	7	0.004894936
		
*Cluster 3: GOs including genes with low expression levels in WT, Scr and KD ESCs and also in KD 12-day EBs as compared with WT and Scr 12-day EBs*		
GO:0007507∼heart development	79	1.70E−18
GO:0001568∼blood vessel development	74	3.02E−13
GO:0060537∼muscle tissue development	48	2.20E−11
GO:0048732∼gland development	59	1.71E−10
GO:0001822∼kidney development	40	1.82E−10
GO:0060348∼bone development	42	3.45E−10
GO:0051216∼cartilage development	32	1.14E−09
GO:0048706∼embryonic skeletal system development	33	1.52E−09
GO:0007420∼brain development	71	2.16E−08
GO:0007417∼central nervous system development	83	9.98E−08
GO:0022008∼neurogenesis	107	8.16E−07
GO:0030324∼lung development	34	1.14E−06
GO:0016055∼Wnt receptor signaling pathway	37	2.45E−06
GO:0017015∼regulation of transforming growth factor-β receptor signaling pathway	14	2.69E−05
		
*Cluster 4: GOs including upregulated in all 12-day EBs populations in comparison with the undifferentiated ESCs populations*		
GO:0001568∼blood vessel development	30	2.17E−05
GO:0001944∼vasculature development	30	3.42E−05
GO:0009966∼regulation of signal transduction	57	1.41E−04
GO:0019220∼regulation of phosphate metabolic process	32	1.76E−04
GO:0045597∼positive regulation of cell differentiation	22	2.54E−04
GO:0030324∼lung development	14	0.00450939
GO:0001822∼kidney development	13	0.0087518
GO:0048666∼neuron development	25	0.01569382
GO:0022008∼neurogenesis	40	0.0181755
		
*Cluster 5: GOs including slightly downregulated genes in the KD 12-day EBs as compared with WT and Scr 12-day EBs*		
GO:0007507∼heart development	18	2.02E−04
GO:0022008∼neurogenesis	30	6.40E−04
mmu04010:MAPK signaling pathway	18	8.45E−04

GOs include genes that are at least twofold up- or downregulated

## Abbreviations

ESC: embryonic stem cell
EB: embryoid body
LIF: leukemia inhibitory factor
TGF-*β*: transforming growth factor-*β*
*α*-MHC: *α*-myosin heavy chain (*Myh6*)
EGFP: enhanced green fluorescent protein
RT-PCR: reverse transcriptase-PCR
shRNA: short hairpin RNA
qPCR: quantitative PCR
FBS: fetal bovine serum
PBS: phosphate-buffered saline
GMEM: Glasgow's minimal essential medium
IMDM: Iscove's modified Dulbecco's medium
ECACC: European Collection of Cell Cultures
aRNA: amplified RNA
DET: differentially expressed transcripts
DAVID: database for annotation, visualization and integrated discovery
TBST: Tris-buffered saline Tween-20
ECL: enhanced chemiluminescence

## Supplementary Material

Supplementary Table S2

Supplementary Movie M1

Supplementary Movie M2
