# Supplementary figures and images for: Fam40b is required for lineage commitment of murine embryonic stem cells
Source: Cell Death Dis. 2014 Jul 10;5(7):e1320–. doi: 10.1038/cddis.2014.273 (PMC4123067; doi:10.1038/cddis.2014.273)

## Slide 1
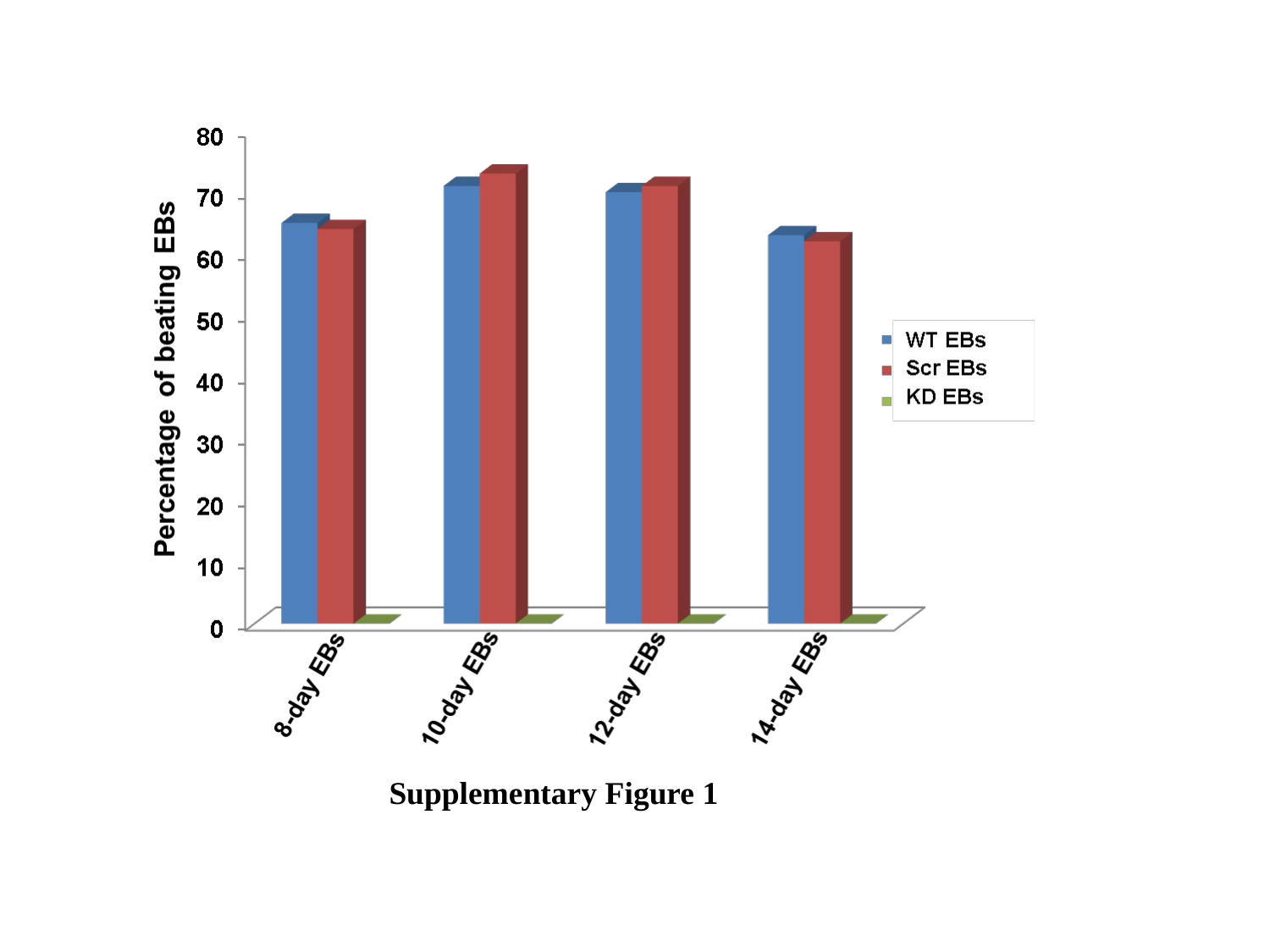

Supplementary Figure 1

Supplement: Supplementary Figure 1 [file cddis2014273x1.ppt]
