# Supplementary material for: Fam40b is required for lineage commitment of murine embryonic stem cells
Source: Cell Death Dis. 2014 Jul 10;5(7):e1320–. doi: 10.1038/cddis.2014.273 (PMC4123067; doi:10.1038/cddis.2014.273)
Supplement: Supplementary Table S1 [file cddis2014273x2.doc]

**Supplementary Table S1**. Statistically validated differentially expressed genes between the Fam40B KD 12-day versus control 12-day old EBs at least 2-fold upregulated or 2-fold downregulated.

| **Probe Set ID** | **Gene Symbol** | **Fold Change (Fam40b KD vs control 12-days EBs )** |
| --- | --- | --- |
| 1450989_at | Tdgf1 | 199 |
| 1418362_at | Zfp42 | 181 |
| 1419418_a_at | Morc1 | 154 |
| 1460226_at | Trap1a | 137 |
| 1429388_at | Nanog | 114 |
| 1417210_at | Eif2s3y | 111 |
| 1415938_at | Spink3 | 97 |
| 1453223_s_at | Dppa2 | 88 |
| 1448949_at | Car4 | 84 |
| 1456242_at | Gm7325 | 83 |
| 1457314_at | L1td1 | 83 |
| 1449534_at | Sycp3 | 81 |
| 1416552_at | Dppa5a | 79 |
| 1460471_at | Ooep | 72 |
| 1417945_at | Pou5f1 | 72 |
| 1427238_at | Fbxo15 | 69 |
| 1438237_at | Gm13138 /// Gm13242 /// LOC100048814 /// Rex2 /// Zfp600 | 63 |
| 1429701_at | Mageb16 | 58 |
| 1456511_x_at | Eras | 58 |
| 1434280_at | --- | 56 |
| 1438820_at | Rnf17 | 56 |
| 1419021_at | Mcf2 | 54 |
| 1429654_at | Dppa2 | 51 |
| 1429366_at | Lrrc34 | 50 |
| 1430735_at | Patl2 | 50 |
| 1443814_x_at | Ctsh | 49 |
| 1426243_at | Cth | 47 |
| 1426438_at | Ddx3y | 47 |
| 1428604_at | 2610305D13Rik | 45 |
| 1436291_a_at | Dpys | 44 |
| 1423281_at | Stmn2 | 41 |
| 1422458_at | Tcl1 | 40 |
| 1428942_at | Mt2 | 39 |
| 1423327_at | Rpl39l | 39 |
| 1418094_s_at | Car4 | 38 |
| 1416023_at | Fabp3 | 38 |
| 1425926_a_at | Otx2 | 38 |
| 1417426_at | Srgn | 36 |
| 1429597_at | Dppa4 | 35 |
| 1427302_at | Enpp3 | 34 |
| 1418365_at | Ctsh | 34 |
| 1423429_at | Rhox5 | 33 |
| 1420086_x_at | Fgf4 | 33 |
| 1420085_at | Fgf4 | 32 |
| 1448499_a_at | Ephx2 | 32 |
| 1449064_at | Tdh | 32 |
| 1436837_at | Mael | 32 |
| 1439065_x_at | Gm13152 | 31 |
| 1417760_at | Nr0b1 | 30 |
| 1417785_at | Pla1a | 30 |
| 1449408_at | Jam2 | 30 |
| 1418756_at | Trh | 29 |
| 1425035_s_at | Dnmt3l | 27 |
| 1455504_a_at | Mkrn1 | 27 |
| 1434739_at | Fmr1nb | 26 |
| 1433966_x_at | Asns | 26 |
| 1456515_s_at | Tcfl5 | 25 |
| 1443892_at | I1C0022H11Rik | 25 |
| 1421113_at | Pga5 | 25 |
| 1450285_at | Gm2098 /// LOC100040390 /// Ube1y1 | 25 |
| 1438868_at | D14Ertd668e | 25 |
| 1434279_at | --- | 24 |
| 1417022_at | Slc7a3 | 24 |
| 1454904_at | Mtm1 | 24 |
| 1460605_at | Crxos1 | 23 |
| 1423424_at | Zic3 | 22 |
| 1422557_s_at | Mt1 | 22 |
| 1457026_at | Liph | 21 |
| 1434278_at | Mtm1 | 20 |
| 1427912_at | Cbr3 | 20 |
| 1426598_at | Uty | 20 |
| 1415857_at | Emb | 19 |
| 1451425_a_at | Mkrn1 | 18 |
| 1429511_at | 4933402E13Rik | 18 |
| 1439260_a_at | Enpp3 | 18 |
| 1448371_at | Mylpf | 18 |
| 1436386_x_at | 1700029I01Rik /// Gm13139 /// Gm13152 /// Gm13212 /// Gm13251 | 18 |
| 1447166_at | --- | 18 |
| 1417346_at | Pycard | 18 |
| 1418994_at | Gtsf1l | 17 |
| 1419018_at | Rhox6 | 17 |
| 1438872_at | Triml1 | 17 |
| 1441885_s_at | --- | 17 |
| 1448562_at | Upp1 | 16 |
| 1417883_at | Gstt2 | 16 |
| 1415856_at | Emb | 16 |
| 1431416_a_at | Jam2 | 16 |
| 1448964_at | S100g | 16 |
| 1427242_at | Ddx4 | 16 |
| 1450871_a_at | Bcat1 | 15 |
| 1438737_at | Zic3 | 15 |
| 1423933_a_at | 1600029D21Rik | 15 |
| 1430233_a_at | Nhedc1 | 15 |
| 1435998_at | Ccnb1ip1 | 15 |
| 1452179_at | Phf17 | 15 |
| 1417837_at | Phlda2 | 15 |
| 1435789_x_at | --- | 14 |
| 1450995_at | Folr1 | 14 |
| 1417079_s_at | Lgals2 | 14 |
| 1448022_at | --- | 14 |
| 1459973_x_at | Dpp4 | 14 |
| 1438883_at | Fgf5 | 14 |
| 1433902_at | Kbtbd8 | 14 |
| 1437752_at | Lin28a | 14 |
| 1451095_at | Asns | 13 |
| 1455961_at | --- | 13 |
| 1452004_at | Calca | 13 |
| 1454114_a_at | Nhedc1 | 13 |
| 1453132_a_at | Gkn2 | 13 |
| 1429483_at | Calcoco2 | 13 |
| 1452270_s_at | Cubn | 13 |
| 1455898_x_at | Slc2a3 | 12 |
| 1416697_at | Dpp4 | 12 |
| 1424265_at | Npl | 12 |
| 1443870_at | Abcc4 | 12 |
| 1447173_at | Lrrc31 | 12 |
| 1452077_at | Ddx3y | 12 |
| 1447934_at | 9630033F20Rik | 12 |
| 1451502_at | Pla2g10 | 12 |
| 1445924_at | Gm13151 /// Gm13235 /// Zfp534 | 12 |
| 1437983_at | Sall1 | 11 |
| 1419758_at | Abcb1a | 11 |
| 1430368_s_at | 1700019D03Rik | 11 |
| 1456140_at | Gm12241 /// LOC100045988 /// Zic5 | 11 |
| 1437870_at | Slco4c1 | 11 |
| 1429001_at | Pir | 11 |
| 1423404_at | Gkn1 | 11 |
| 1417561_at | Apoc1 | 11 |
| 1444390_at | Prdm14 | 11 |
| 1444292_at | LOC100303645 | 11 |
| 1433789_at | Snhg3 | 11 |
| 1419665_a_at | Nupr1 | 11 |
| 1428304_at | Esco2 | 11 |
| 1460181_at | Stmn3 | 11 |
| 1418435_at | Mkrn1 | 10 |
| 1424295_at | Dppa3 | 10 |
| 1425140_at | Lactb2 | 10 |
| 1426753_at | Phf17 | 10 |
| 1431393_at | Six6os1 | 10 |
| 1428459_at | Pramef12 | 10 |
| 1449288_at | Gdf3 | 10 |
| 1456219_at | LOC100045988 /// Zic5 | 10 |
| 1429203_at | 2410076I21Rik | 10 |
| 1419086_at | Fgfbp1 | 10 |
| 1417185_at | Ly6a | 10 |
| 1435783_at | Fam169a | 10 |
| 1422682_s_at | EG436523 /// Gm10334 /// Prss1 /// Prss3 | 10 |
| 1426752_at | Phf17 | 10 |
| 1454215_at | 2410007B07Rik | 9 |
| 1438824_at | Slc20a1 | 9 |
| 1419430_at | Cyp26a1 | 9 |
| 1436287_at | Gm10664 | 9 |
| 1434353_at | Sfmbt2 | 9 |
| 1452180_at | Phf17 | 9 |
| 1452384_at | Enpp3 | 9 |
| 1436419_a_at | 1700097N02Rik | 9 |
| 1417482_at | Tex19.1 | 9 |
| 1421317_x_at | Myb | 9 |
| 1450645_at | Mt4 | 9 |
| 1429891_at | Capsl | 9 |
| 1436619_at | D630045M09Rik | 9 |
| 1416041_at | Sgk1 | 8 |
| 1418091_at | Tcfcp2l1 | 8 |
| 1428640_at | Hsf2bp | 8 |
| 1423925_at | Dhx16 | 8 |
| 1436926_at | Esrrb | 8 |
| 1447997_s_at | Timm8a2 | 8 |
| 1426439_at | Ddx3y | 8 |
| 1454138_a_at | Stk31 | 8 |
| 1449253_at | Smc1b | 8 |
| 1437779_at | Foxh1 | 8 |
| 1450744_at | Ell2 | 8 |
| 1427170_at | Psma8 | 8 |
| 1424524_at | Dram1 | 8 |
| 1437052_s_at | Slc2a3 | 8 |
| 1430780_a_at | Pmm1 | 8 |
| 1417888_at | Trim13 | 8 |
| 1426808_at | Lgals3 | 8 |
| 1424448_at | Trim6 | 8 |
| 1418423_s_at | Gm11397 /// Serpinb9e /// Serpinb9f /// Serpinb9g | 8 |
| 1421882_a_at | Elavl2 | 8 |
| 1416049_at | Gldc | 8 |
| 1418872_at | Abcb1b | 8 |
| 1436227_at | Lefty2 | 8 |
| 1435113_x_at | Stmn3 | 8 |
| 1424167_a_at | Pmm1 | 8 |
| 1436574_at | 1700029I01Rik | 8 |
| 1421307_at | Car13 | 8 |
| 1421883_at | Elavl2 | 8 |
| 1416382_at | Ctsc | 8 |
| 1422058_at | Nodal | 7 |
| 1416605_at | Nhp2 | 7 |
| 1451348_at | Depdc6 | 7 |
| 1450997_at | Stk17b | 7 |
| 1422663_at | Orc1l | 7 |
| 1429932_at | 4930566F21Rik | 7 |
| 1452606_at | Mnd1 | 7 |
| 1427760_s_at | Prl2c2 /// Prl2c3 /// Prl2c4 | 7 |
| 1441317_x_at | Jakmip1 | 7 |
| 1441429_at | Irs4 | 7 |
| 1435695_a_at | Ggct | 7 |
| 1452203_at | Obfc2a | 7 |
| 1451335_at | Plac8 | 7 |
| 1426712_at | Slc6a15 | 7 |
| 1460711_at | Dnajc21 | 7 |
| 1435172_at | Eomes | 7 |
| 1425503_at | Gcnt2 | 7 |
| 1417836_at | Gpx7 | 7 |
| 1417656_at | Mybl2 | 7 |
| 1449052_a_at | Dnmt3b | 7 |
| 1450626_at | Manba | 7 |
| 1441023_at | Eif2s2 | 7 |
| 1438933_x_at | Rasgrp2 | 7 |
| 1439881_at | C030013E06Rik | 7 |
| 1418422_at | Serpinb9g | 7 |
| 1437019_at | 2200001I15Rik | 7 |
| 1447818_x_at | Rhebl1 | 7 |
| 1416776_at | Crym | 7 |
| 1422462_at | Ube2t | 7 |
| 1451139_at | Slc39a4 | 7 |
| 1455425_at | Tet1 | 7 |
| 1453840_at | Pabpc1 | 7 |
| 1443052_at | C330019L16Rik /// Gm2381 | 7 |
| 1456179_at | Gm13212 | 7 |
| 1424683_at | Fam134b | 7 |
| 1439620_at | Car13 | 7 |
| 1449254_at | Spp1 | 6 |
| 1451968_at | Xrcc5 | 6 |
| 1427603_at | Atf7ip2 | 6 |
| 1453219_a_at | Tdrd12 | 6 |
| 1419304_at | T | 6 |
| 1443579_s_at | Depdc6 | 6 |
| 1447704_s_at | Utp23 | 6 |
| 1423627_at | Nqo1 | 6 |
| 1434528_at | Aard | 6 |
| 1449364_at | Aurkc | 6 |
| 1435483_x_at | Slc25a32 | 6 |
| 1441836_x_at | Gtsf1 | 6 |
| 1415802_at | Slc16a1 | 6 |
| 1451791_at | Tfpi | 6 |
| 1433557_at | Cbx7 | 6 |
| 1425536_at | Stx3 | 6 |
| 1441957_x_at | 2410076I21Rik | 6 |
| 1438527_at | Gm12816 /// Gm5879 /// Rpl3 | 6 |
| 1438516_at | Rif1 | 6 |
| 1438190_x_at | Tpbpa | 6 |
| 1419666_x_at | Nupr1 | 6 |
| 1455692_x_at | 1700097N02Rik | 6 |
| 1419149_at | Serpine1 | 6 |
| 1421895_at | Eif2s3x /// LOC100048746 | 6 |
| 1449968_s_at | Acot10 /// Acot9 | 6 |
| 1448107_x_at | Klk1 | 6 |
| 1419542_at | Dazl | 6 |
| 1436585_at | BB182297 | 6 |
| 1415837_at | Klk1 | 6 |
| 1452320_at | Lrp2 | 6 |
| 1446526_at | 9330199F22Rik | 6 |
| 1447021_at | --- | 6 |
| 1460431_at | Gcnt1 | 6 |
| 1459260_at | Gm13212 | 6 |
| 1419929_at | D15Ertd55e | 6 |
| 1437786_at | Naa11 | 6 |
| 1456942_x_at | Gm9112 | 6 |
| 1426990_at | Cubn | 6 |
| 1434853_x_at | Mkrn1 | 6 |
| 1444051_at | 1700019D03Rik | 6 |
| 1418310_a_at | Rlbp1 | 6 |
| 1448752_at | Car2 | 6 |
| 1428706_at | Cenpv | 6 |
| 1455102_at | Larp4 | 6 |
| 1421308_at | Car13 | 6 |
| 1454254_s_at | 1600029D21Rik | 6 |
| 1450943_at | Magohb | 6 |
| 1423861_at | Plekhf2 | 6 |
| 1455593_at | Apob | 6 |
| 1455185_s_at | Phf16 | 6 |
| 1428052_a_at | Zmym1 | 6 |
| 1439362_at | --- | 6 |
| 1451323_at | Zfp7 | 6 |
| 1420873_at | Twf1 | 6 |
| 1442939_at | Rif1 | 6 |
| 1417374_at | Tuba4a | 6 |
| 1438084_at | --- | 6 |
| 1417470_at | Apobec3 | 6 |
| 1417089_a_at | Ckmt1 | 6 |
| 1419106_at | 2210409E12Rik | 6 |
| 1417823_at | Gcat | 5 |
| 1449344_s_at | 2210409E12Rik | 5 |
| 1435154_at | AU018091 | 5 |
| 1452912_at | Dscc1 | 5 |
| 1439150_x_at | Grtp1 | 5 |
| 1449819_at | Dmc1 | 5 |
| 1459275_at | Rnf17 | 5 |
| 1448136_at | Enpp2 | 5 |
| 1423158_at | Gnpnat1 | 5 |
| 1417373_a_at | Tuba4a | 5 |
| 1450505_a_at | Fam134b | 5 |
| 1426806_at | Obfc2a | 5 |
| 1437179_at | Rif1 | 5 |
| 1419759_at | Abcb1a | 5 |
| 1427543_s_at | Gm2098 /// LOC100040390 /// Ube1y1 | 5 |
| 1449540_at | Rhox9 | 5 |
| 1424921_at | Bst2 | 5 |
| 1450194_a_at | Myb | 5 |
| 1427482_a_at | Car8 | 5 |
| 1417300_at | Smpdl3b | 5 |
| 1416242_at | Klhl13 | 5 |
| 1425891_a_at | Grtp1 | 5 |
| 1452324_at | Pvt1 | 5 |
| 1424303_at | Depdc7 | 5 |
| 1455904_at | Gas5 /// Snord47 | 5 |
| 1450752_at | Cyct | 5 |
| 1453069_at | Pik3cb | 5 |
| 1440319_at | Rbm44 | 5 |
| 1416326_at | Crip1 | 5 |
| 1451516_at | Rhebl1 | 5 |
| 1436392_s_at | Tcfap2c | 5 |
| 1443673_x_at | --- | 5 |
| 1416832_at | Slc39a8 | 5 |
| 1429490_at | Rif1 | 5 |
| 1424784_at | Gm13139 | 5 |
| 1434559_at | Stx3 | 5 |
| 1418351_a_at | Dnmt3b | 5 |
| 1418380_at | Terf1 | 5 |
| 1418073_at | Acot9 | 5 |
| 1448890_at | Klf2 | 5 |
| 1423156_at | Gnpnat1 | 5 |
| 1460468_s_at | Dnajc22 | 5 |
| 1448665_at | Dmd | 5 |
| 1431896_at | Six6os1 | 5 |
| 1449434_at | Car3 | 5 |
| 1418734_at | BE136769 | 5 |
| 1432503_a_at | Pdcl2 | 5 |
| 1457582_at | Uty | 5 |
| 1444779_s_at | Zfp59 | 5 |
| 1434296_at | BC049349 | 5 |
| 1441879_x_at | Mkrn1 | 5 |
| 1450016_at | Ccng1 | 5 |
| 1453628_s_at | Lrrc2 | 5 |
| 1433596_at | Dnajc6 | 5 |
| 1435099_at | Utp14a | 5 |
| 1417828_at | Aqp8 | 5 |
| 1424008_a_at | Rbpms2 | 5 |
| 1431417_at | Jam2 | 5 |
| 1449357_at | 2310030G06Rik | 5 |
| 1428660_s_at | LOC100047963 /// Tor3a | 5 |
| 1431332_a_at | Terf1 | 5 |
| 1417541_at | Hells | 5 |
| 1417501_at | Fbxo6 | 5 |
| 1429259_a_at | 1190007I07Rik /// 1810014B01Rik | 5 |
| 1439759_x_at | Sult6b1 | 5 |
| 1431980_a_at | As3mt | 5 |
| 1437015_x_at | Pla2g1b | 5 |
| 1435679_at | Optn | 5 |
| 1416066_at | Cd9 | 5 |
| 1456853_at | BC024063 /// Gm4924 | 5 |
| 1434976_x_at | Eif4ebp1 | 5 |
| 1449342_at | Ptplb | 5 |
| 1425964_x_at | Hspb1 | 5 |
| 1425317_x_at | Stk31 | 5 |
| 1432361_a_at | Cenpp | 5 |
| 1449455_at | Hck | 5 |
| 1453558_at | Efcab10 | 5 |
| 1457281_at | Dnajc21 | 5 |
| 1436194_at | Prelid2 | 5 |
| 1424792_at | Rpp40 | 5 |
| 1452609_at | 1190005I06Rik | 4 |
| 1429564_at | LOC100048247 /// Pcgf5 | 4 |
| 1420342_at | Gdap10 | 4 |
| 1430162_at | 3830417A13Rik | 4 |
| 1428515_at | Zswim7 | 4 |
| 1455342_at | Prune2 | 4 |
| 1434681_at | 4932441K18Rik | 4 |
| 1433914_at | AI747699 | 4 |
| 1427133_s_at | Lrp2 | 4 |
| 1434524_at | Eif2b3 | 4 |
| 1424966_at | Tmem40 | 4 |
| 1417103_at | Ddt | 4 |
| 1420119_s_at | Phf3 | 4 |
| 1451594_s_at | Serpinb6c | 4 |
| 1423452_at | Stk17b | 4 |
| 1452454_at | Sdad1 | 4 |
| 1436799_at | Enox1 | 4 |
| 1428061_at | Hat1 | 4 |
| 1449155_at | Polr3g | 4 |
| 1426572_at | Me2 | 4 |
| 1419722_at | Klk8 | 4 |
| 1453149_at | Slc25a32 | 4 |
| 1427388_at | Lrrc2 | 4 |
| 1416606_s_at | Nhp2 | 4 |
| 1428341_at | Zfp655 | 4 |
| 1425318_a_at | Tmem116 | 4 |
| 1431701_a_at | Pdzk1 | 4 |
| 1419240_at | Tex14 | 4 |
| 1435442_at | Dcaf13 | 4 |
| 1437497_a_at | Hsp90aa1 | 4 |
| 1442630_at | --- | 4 |
| 1441971_at | --- | 4 |
| 1433545_s_at | Acad11 /// Nphp3 | 4 |
| 1451790_a_at | Tfpi | 4 |
| 1429448_s_at | Tet1 | 4 |
| 1448414_at | Rad1 | 4 |
| 1455195_at | Rps24 | 4 |
| 1419968_at | C77370 | 4 |
| 1427044_a_at | Amph | 4 |
| 1448133_at | Nmd3 | 4 |
| 1452017_at | Sox15 | 4 |
| 1449972_s_at | BC018101 /// Zfp97 | 4 |
| 1448609_at | Tst | 4 |
| 1451064_a_at | Psat1 | 4 |
| 1417741_at | Pygl | 4 |
| 1429095_at | Cenpp | 4 |
| 1450428_at | Lhx1 | 4 |
| 1434365_a_at | BC055324 | 4 |
| 1425530_a_at | Stx3 | 4 |
| 1426052_at | Mlh3 | 4 |
| 1452007_at | Vamp7 | 4 |
| 1443256_at | --- | 4 |
| 1438349_at | Zfp229 | 4 |
| 1415830_at | Orc5l | 4 |
| 1444038_at | AU015836 | 4 |
| 1434836_at | Nfatc2ip | 4 |
| 1451144_at | Brix1 | 4 |
| 1417562_at | Eif4ebp1 | 4 |
| 1433408_a_at | Mcm10 | 4 |
| 1437552_at | 2410127L17Rik /// LOC677553 | 4 |
| 1419544_at | Atp6v1c1 | 4 |
| 1437238_x_at | Nmd3 | 4 |
| 1423169_at | Taf7 | 4 |
| 1439753_x_at | Six4 | 4 |
| 1420919_at | Sgk3 | 4 |
| 1426696_at | Lrpap1 | 4 |
| 1448568_a_at | Slc20a1 | 4 |
| 1460467_at | Iqcg | 4 |
| 1417254_at | Spata5 | 4 |
| 1417027_at | Trim2 | 4 |
| 1429154_at | LOC100048307 /// Slc35f2 | 4 |
| 1441279_at | C430002E04Rik | 4 |
| 1441917_s_at | Tmem40 | 4 |
| 1444837_at | --- | 4 |
| 1429830_a_at | Cd59a | 4 |
| 1445660_at | Tmem209 | 4 |
| 1426645_at | Hsp90aa1 | 4 |
| 1416052_at | Prps1 | 4 |
| 1422943_a_at | Hspb1 | 4 |
| 1424211_at | Slc25a33 | 4 |
| 1423120_at | Ide | 4 |
| 1417684_at | Thumpd3 | 4 |
| 1418434_at | Mkrn1 | 4 |
| 1454647_at | Acad11 /// Nphp3 | 4 |
| 1454607_s_at | Psat1 | 4 |
| 1423747_a_at | Pdk1 | 4 |
| 1420774_a_at | 4930583H14Rik | 4 |
| 1421498_a_at | 2010204K13Rik | 4 |
| 1425312_s_at | Cpsf4l | 4 |
| 1457945_at | --- | 4 |
| 1426137_at | Gm13138 /// Rex2 | 4 |
| 1441269_at | --- | 4 |
| 1426151_a_at | Stx3 | 4 |
| 1439173_at | Hook1 | 4 |
| 1432057_a_at | Prdm5 | 4 |
| 1450948_a_at | Mrpl1 | 4 |
| 1418640_at | Sirt1 | 4 |
| 1419537_at | Tcfec | 4 |
| 1451058_at | Mcts2 | 4 |
| 1417190_at | Nampt | 4 |
| 1460323_at | Tars | 4 |
| 1452256_at | 1110002N22Rik | 4 |
| 1419814_s_at | S100a1 | 4 |
| 1456208_at | Gpat2 | 4 |
| 1439972_at | Etnk1 | 4 |
| 1417638_at | Lefty1 | 4 |
| 1429655_at | Nudcd1 | 4 |
| 1426626_at | Gtf2f2 | 4 |
| 1436794_at | Nlrp4f | 4 |
| 1435412_at | 1700007E06Rik | 4 |
| 1424955_at | Haus1 | 4 |
| 1419194_s_at | Gmfg | 4 |
| 1448646_at | Wdr12 | 4 |
| 1429491_s_at | Rif1 | 4 |
| 1452242_at | Cep55 | 4 |
| 1417074_at | Ceacam10 | 4 |
| 1449204_at | Gjb5 | 4 |
| 1457100_at | AW552889 | 4 |
| 1453360_a_at | Tex9 | 4 |
| 1438757_at | Gm4636 | 4 |
| 1451612_at | Mt1 | 4 |
| 1452458_s_at | Ppil5 | 4 |
| 1432454_at | 2410137M14Rik | 4 |
| 1429778_at | Optn | 4 |
| 1423581_at | Nmt2 | 4 |
| 1456862_at | Six4 | 4 |
| 1416138_at | Anxa7 | 4 |
| 1456266_at | --- | 4 |
| 1420616_at | Ash2l | 4 |
| 1427046_at | Grhl2 | 4 |
| 1417392_a_at | Slc7a7 | 4 |
| 1437250_at | Mreg | 4 |
| 1430111_a_at | Bcat1 | 4 |
| 1456143_at | Zmynd8 | 4 |
| 1460576_at | Exoc6 | 4 |
| 1424152_at | Sall4 | 4 |
| 1428629_at | Zfp518 | 4 |
| 1425098_at | Zfp106 | 4 |
| 1418184_at | Cenpm | 4 |
| 1436922_at | Ppil5 | 4 |
| 1450039_at | Usp9x | 4 |
| 1442739_at | BC031441 | 4 |
| 1418069_at | Apoc2 | 4 |
| 1428951_at | Nol8 | 4 |
| 1449119_at | Arih2 | 4 |
| 1444548_at | 1600025M17Rik | 4 |
| 1442571_at | --- | 4 |
| 1455259_a_at | Utp23 | 4 |
| 1415808_at | Tpbpa | 4 |
| 1448653_at | Eed | 4 |
| 1456804_at | Gm6792 | 4 |
| 1417453_at | Cul4b | 4 |
| 1429278_at | Nubpl | 4 |
| 1451602_at | Snx6 | 4 |
| 1427477_at | Tmprss13 | 4 |
| 1437186_at | BC055324 | 4 |
| 1424971_at | Ccdc99 | 4 |
| 1431893_a_at | Pdss1 | 4 |
| 1456251_x_at | Tspo | 4 |
| 1442740_at | Prdm5 | 4 |
| 1427399_a_at | Nxf7 | 4 |
| 1448551_a_at | Trim2 | 4 |
| 1440681_at | Chrna7 | 4 |
| 1428622_at | Depdc6 | 4 |
| 1435211_at | Ttc12 | 4 |
| 1435308_at | Fut9 | 4 |
| 1429961_at | 1700021C14Rik | 4 |
| 1440910_at | C77370 | 4 |
| 1442588_at | Gm5101 | 4 |
| 1428685_at | Syce1 | 4 |
| 1456311_x_at | Esco2 | 4 |
| 1439361_at | --- | 4 |
| 1435766_at | Pphln1 | 4 |
| 1449538_a_at | Gcnt1 | 4 |
| 1438511_a_at | 1190002H23Rik | 4 |
| 1452968_at | Cthrc1 | 4 |
| 1448842_at | Cdo1 | 4 |
| 1438018_at | Hook1 | 4 |
| 1434970_a_at | Mrpl15 | 4 |
| 1424202_at | Seh1l | 4 |
| 1424343_a_at | Eif1a | 4 |
| 1448181_at | Klf15 | 4 |
| 1419706_a_at | Akap12 | 4 |
| 1438948_x_at | Tspo | 4 |
| 1458687_at | Gm9564 | 4 |
| 1455320_at | Nampt | 4 |
| 1456617_a_at | Eif2s2 | 4 |
| 1453299_a_at | LOC100045567 /// Pnp /// Pnp2 | 4 |
| 1419254_at | Mthfd2 | 4 |
| 1418836_at | Qprt | 4 |
| 1417267_s_at | Fkbp11 | 4 |
| 1436763_a_at | Klf9 | 4 |
| 1434435_s_at | Cox17 | 4 |
| 1428599_at | Kndc1 | 4 |
| 1457869_at | Gm10451 | 4 |
| 1435302_at | Taf4b | 4 |
| 1437340_x_at | Gkn1 | 4 |
| 1442071_at | Abce1 | 4 |
| 1449276_at | 1700029P11Rik | 4 |
| 1441827_x_at | Hsp90aa1 | 4 |
| 1419241_a_at | Aire | 4 |
| 1458599_at | --- | 4 |
| 1432188_s_at | Nup43 | 4 |
| 1428950_s_at | Nol8 | 4 |
| 1452224_at | Morc3 | 4 |
| 1417712_at | Eif2s2 | 3 |
| 1423187_at | Gabarapl2 | 3 |
| 1448269_a_at | Klhl13 | 3 |
| 1448736_a_at | Hprt | 3 |
| 1452799_at | Fggy | 3 |
| 1433720_s_at | Chchd10 | 3 |
| 1417078_at | Lgals2 | 3 |
| 1451531_at | Cbwd1 | 3 |
| 1443945_at | C130073F10Rik | 3 |
| 1416076_at | Ccnb1 /// Gm5593 /// Gm8416 | 3 |
| 1417045_at | Bid | 3 |
| 1422668_at | Serpinb9b | 3 |
| 1431648_at | 4930528F23Rik | 3 |
| 1453361_at | Hells | 3 |
| 1422264_s_at | Klf9 | 3 |
| 1423259_at | Id4 /// LOC100045546 | 3 |
| 1418203_at | Pmaip1 | 3 |
| 1456891_at | Dennd2c | 3 |
| 1436351_at | Coq3 | 3 |
| 1428390_at | Wdr43 | 3 |
| 1431786_s_at | 1190003J15Rik | 3 |
| 1431672_at | 9430069I07Rik | 3 |
| 1430314_at | 4933437F05Rik | 3 |
| 1450947_at | 2610528J11Rik | 3 |
| 1443128_at | Ints6 | 3 |
| 1429937_at | Utp23 | 3 |
| 1419585_at | Rp2h | 3 |
| 1416554_at | LOC100048338 /// Pdlim1 | 3 |
| 1424344_s_at | Eif1a | 3 |
| 1423708_a_at | Farsb | 3 |
| 1428142_at | Etv5 | 3 |
| 1449481_at | Slc25a13 | 3 |
| 1417437_at | Xrcc6 | 3 |
| 1424340_at | Lrrc48 | 3 |
| 1417408_at | F3 | 3 |
| 1452974_at | Nol8 | 3 |
| 1424172_at | Hagh | 3 |
| 1425189_a_at | Mrpl15 | 3 |
| 1426741_a_at | Fastkd2 | 3 |
| 1435103_x_at | Farsb | 3 |
| 1423625_a_at | Dnajc19 | 3 |
| 1454817_at | Utp18 | 3 |
| 1448126_at | Fam60a | 3 |
| 1417661_at | Rdm1 | 3 |
| 1435755_at | 1110001A16Rik | 3 |
| 1427878_at | 0610010O12Rik | 3 |
| 1451208_at | Etf1 | 3 |
| 1460242_at | Cd55 | 3 |
| 1417370_at | Tff3 | 3 |
| 1418727_at | Nup155 | 3 |
| 1451409_at | 2210021J22Rik | 3 |
| 1421524_at | Cfc1 | 3 |
| 1423142_a_at | Gtpbp4 | 3 |
| 1434910_at | A830080D01Rik | 3 |
| 1455186_a_at | 1190003J15Rik | 3 |
| 1455621_at | Zfp870 | 3 |
| 1448820_a_at | Eif2s2 /// Gm9892 /// LOC100046208 | 3 |
| 1419289_a_at | Syngr1 | 3 |
| 1422809_at | Rims2 | 3 |
| 1448409_at | Lrmp | 3 |
| 1428083_at | Neat1 | 3 |
| 1429833_at | Ly6g6e | 3 |
| 1418191_at | LOC100048346 /// Usp18 | 3 |
| 1442721_at | --- | 3 |
| 1439819_at | AU015263 | 3 |
| 1420922_at | Usp9x | 3 |
| 1427881_at | Dnttip2 | 3 |
| 1448237_x_at | Ldhb | 3 |
| 1449044_at | Eef1e1 | 3 |
| 1419278_at | Usp48 | 3 |
| 1438360_x_at | Gm5256 /// Gm5529 /// Slc25a5 | 3 |
| 1451320_at | Arhgap8 | 3 |
| 1438545_at | Gm5529 /// Slc25a5 | 3 |
| 1416953_at | Ctgf | 3 |
| 1417737_at | Mrps31 | 3 |
| 1416757_at | Zwilch | 3 |
| 1428543_at | Ppat | 3 |
| 1433465_a_at | AI467606 | 3 |
| 1439010_at | Larp4 | 3 |
| 1420388_at | Prss12 | 3 |
| 1428315_at | Ebna1bp2 | 3 |
| 1443621_at | Xaf1 | 3 |
| 1443924_at | --- | 3 |
| 1438786_a_at | 2610021A01Rik | 3 |
| 1424938_at | Steap1 | 3 |
| 1430364_at | Atf7ip2 | 3 |
| 1417028_a_at | Trim2 | 3 |
| 1442775_at | --- | 3 |
| 1447097_at | Gm3609 | 3 |
| 1451751_at | Ddit4l | 3 |
| 1448713_at | Stat4 | 3 |
| 1426573_at | Me2 | 3 |
| 1452432_at | Tfpi | 3 |
| 1417233_at | Chchd4 | 3 |
| 1418152_at | Hmgn5 | 3 |
| 1432187_at | Nup43 | 3 |
| 1449137_at | Pdha1 | 3 |
| 1420509_at | Srfbp1 | 3 |
| 1439695_a_at | Kif20b | 3 |
| 1448889_at | Slc38a4 | 3 |
| 1459860_x_at | Trim2 | 3 |
| 1418323_at | Fem1b | 3 |
| 1418681_at | Alg13 | 3 |
| 1419257_at | Tcea1 | 3 |
| 1433982_at | Usp28 | 3 |
| 1435374_at | Cdyl2 | 3 |
| 1450873_at | Gtpbp4 | 3 |
| 1452172_at | Fastkd2 | 3 |
| 1437936_at | Ccdc160 | 3 |
| 1448528_at | Pdcd10 | 3 |
| 1418627_at | Gclm | 3 |
| 1425213_at | Fam81a | 3 |
| 1425331_at | Zfp106 | 3 |
| 1427524_a_at | Mphosph8 | 3 |
| 1450665_at | Gabpa | 3 |
| 1416967_at | Sox2 | 3 |
| 1424420_at | Ccpg1 | 3 |
| 1428452_at | 2810025M15Rik | 3 |
| 1421924_at | Slc2a3 | 3 |
| 1423170_at | Taf7 | 3 |
| 1423582_at | Dmrt1 | 3 |
| 1448571_a_at | Gmfb | 3 |
| 1438035_at | Fam82a1 | 3 |
| 1453600_at | Ccdc18 | 3 |
| 1423276_at | Ildr1 | 3 |
| 1417804_at | Rasgrp2 | 3 |
| 1434317_s_at | Tex10 | 3 |
| 1416948_at | Mrpl23 | 3 |
| 1424777_at | Dcaf4 | 3 |
| 1429458_at | 2410127L17Rik /// LOC677553 | 3 |
| 1436990_s_at | Chchd10 | 3 |
| 1451695_a_at | Gpx4 | 3 |
| 1443750_s_at | Rpp40 | 3 |
| 1424171_a_at | Hagh | 3 |
| 1424400_a_at | Aldh1l1 /// LOC100047937 | 3 |
| 1448604_at | Uck2 | 3 |
| 1418986_a_at | Uxt | 3 |
| 1419546_at | Atp6v1c1 | 3 |
| 1442059_at | Fxr1 | 3 |
| 1448192_s_at | Gm5081 /// Prps1 | 3 |
| 1451576_at | Prkdc | 3 |
| 1424235_at | Ormdl2 | 3 |
| 1438922_x_at | Gm5256 /// Gm5529 /// Slc25a5 | 3 |
| 1448844_at | Cyb5b | 3 |
| 1417511_at | Lyar | 3 |
| 1452583_s_at | Galm | 3 |
| 1419943_s_at | Ccnb1 | 3 |
| 1435057_x_at | Polr1e | 3 |
| 1441501_at | Serbp1 | 3 |
| 1455035_s_at | Nop56 | 3 |
| 1422653_at | Cep70 | 3 |
| 1423465_at | Frrs1 /// LOC100046401 | 3 |
| 1449128_at | Ccdc43 | 3 |
| 1449170_at | Piwil2 | 3 |
| 1432556_a_at | Fam183b | 3 |
| 1429137_at | 2810422O20Rik | 3 |
| 1449568_at | Klb | 3 |
| 1451065_a_at | Ddx39 | 3 |
| 1437177_at | Larp4 | 3 |
| 1456225_x_at | Trib3 | 3 |
| 1434422_at | 1700066M21Rik | 3 |
| 1438740_at | Nmt2 | 3 |
| 1455674_at | Wdr76 | 3 |
| 1460353_at | Tmem48 | 3 |
| 1445281_a_at | B230311B06Rik | 3 |
| 1417635_at | Spa17 | 3 |
| 1421881_a_at | Elavl2 | 3 |
| 1419058_at | Polr1e | 3 |
| 1436964_at | D7Ertd715e | 3 |
| 1418003_at | 1190002H23Rik | 3 |
| 1452662_a_at | Eif2s1 | 3 |
| 1416183_a_at | Ldhb | 3 |
| 1451649_a_at | Wdr75 | 3 |
| 1424149_at | Nsmce2 | 3 |
| 1425591_a_at | Chmp2a | 3 |
| 1434971_x_at | Mrpl15 | 3 |
| 1416411_at | Gstm2 | 3 |
| 1423709_s_at | Farsb | 3 |
| 1441045_at | Ddx43 | 3 |
| 1424719_a_at | Mapt | 3 |
| 1452246_at | Ostf1 | 3 |
| 1458347_s_at | Tmprss2 | 3 |
| 1420091_s_at | Morc3 | 3 |
| 1421100_a_at | Dab1 | 3 |
| 1429156_at | 2610036L11Rik | 3 |
| 1452839_at | Dph5 | 3 |
| 1417886_at | 1810009A15Rik | 3 |
| 1418583_at | Hint3 | 3 |
| 1426797_at | Tmem209 | 3 |
| 1447483_s_at | Snhg7 | 3 |
| 1450423_s_at | Rpf2 | 3 |
| 1460722_at | Soat2 | 3 |
| 1423143_at | Gtpbp4 | 3 |
| 1424999_at | 1700022C21Rik | 3 |
| 1434046_at | AA467197 | 3 |
| 1452034_at | Prepl | 3 |
| 1424529_s_at | Cgref1 | 3 |
| 1420023_at | Etf1 | 3 |
| 1449031_at | Cited1 | 3 |
| 1416899_at | Utf1 | 3 |
| 1446085_at | --- | 3 |
| 1427213_at | Pfkfb1 | 3 |
| 1428825_at | Nr6a1 | 3 |
| 1453011_at | Bdh2 | 3 |
| 1432174_a_at | Nhedc1 | 3 |
| 1440461_at | --- | 3 |
| 1449032_at | Prl2a1 | 3 |
| 1432511_s_at | Haus2 | 3 |
| 1442655_at | Dnmt3b | 3 |
| 1417307_at | Dmd | 3 |
| 1422734_a_at | Myb | 3 |
| 1415882_at | Ghitm | 3 |
| 1436609_a_at | Lrpap1 | 3 |
| 1418387_at | Mphosph8 | 3 |
| 1415673_at | Psph | 3 |
| 1436684_a_at | Riok2 | 3 |
| 1460519_a_at | Mettl5 | 3 |
| 1456193_x_at | Gpx4 | 3 |
| 1423643_at | Ddx39 | 3 |
| 1430515_s_at | Aasdhppt | 3 |
| 1452585_at | Mrps28 | 3 |
| 1423280_at | Stmn2 | 3 |
| 1420499_at | Gch1 | 3 |
| 1428566_at | Hspbap1 | 3 |
| 1429171_a_at | Ncapg | 3 |
| 1456590_x_at | Akr1b3 | 3 |
| 1421945_a_at | Rpf2 | 3 |
| 1431352_s_at | Pvt1 | 3 |
| 1435655_at | Rpl12 | 3 |
| 1424176_a_at | Anxa4 | 3 |
| 1434150_a_at | Higd1c /// Mettl7a1 /// Mettl7a2 | 3 |
| 1437479_x_at | Tbx3 | 3 |
| 1449694_s_at | Commd5 | 3 |
| 1454866_s_at | Clic6 | 3 |
| 1419930_at | D15Ertd55e | 3 |
| 1433921_s_at | Dph3 | 3 |
| 1416849_at | Cisd1 | 3 |
| 1448271_a_at | Ddx21 | 3 |
| 1421830_at | Ak3l1 /// LOC100047616 | 3 |
| 1422057_at | Nodal | 3 |
| 1424229_at | Dyrk3 | 3 |
| 1428705_at | 1700007K13Rik | 3 |
| 1419154_at | Tmprss2 | 3 |
| 1423754_at | Ifitm3 | 3 |
| 1451164_a_at | Mrps18b | 3 |
| 1416043_at | Nasp | 3 |
| 1416706_at | Rpe | 3 |
| 1422798_at | Cntnap2 | 3 |
| 1448169_at | Krt18 | 3 |
| 1416915_at | Msh6 | 3 |
| 1418226_at | Orc2l | 3 |
| 1424254_at | Ifitm1 | 3 |
| 1434499_a_at | Ldhb | 3 |
| 1449115_at | Mtf2 | 3 |
| 1438015_at | Dkc1 | 3 |
| 1438319_x_at | Fastkd2 | 3 |
| 1418507_s_at | Socs2 | 3 |
| 1431252_a_at | Zfp655 | 3 |
| 1426510_at | LOC100044896 /// Sccpdh | 3 |
| 1428518_at | Mlf1ip | 3 |
| 1416762_at | S100a10 | 3 |
| 1433656_a_at | Gnl3 | 3 |
| 1451611_at | Pla2g16 | 3 |
| 1420427_a_at | Dhx32 | 3 |
| 1453041_at | Ano9 | 3 |
| 1433794_at | Setx | 3 |
| 1438005_at | Slc25a40 | 3 |
| 1419235_s_at | Helb | 3 |
| 1424201_a_at | Seh1l | 3 |
| 1430238_at | Got1l1 | 3 |
| 1450082_s_at | Etv5 | 3 |
| 1428749_at | Dmxl2 | 3 |
| 1441272_at | Matr3 | 3 |
| 1438844_x_at | Spata5 | 3 |
| 1423946_at | Pdlim2 | 3 |
| 1419798_at | 2610019E17Rik | 3 |
| 1434292_at | Snhg11 | 3 |
| 1416346_at | Timm8a1 | 3 |
| 1448740_at | Rangrf | 3 |
| 1455235_x_at | Ldhb | 3 |
| 1424046_at | Bub1 | 3 |
| 1424300_at | Gemin6 | 3 |
| 1455039_a_at | Sin3b | 3 |
| 1455814_x_at | Ddx39 | 3 |
| 1419545_a_at | Atp6v1c1 | 3 |
| 1426533_at | Nop56 | 3 |
| 1434173_s_at | D19Bwg1357e | 3 |
| 1448849_at | Mrpl40 | 3 |
| 1453001_at | Rnaseh2b | 3 |
| 1418334_at | Dbf4 | 3 |
| 1428289_at | Klf9 | 3 |
| 1449575_a_at | Gstp1 | 3 |
| 1450986_at | Nop58 | 3 |
| 1452215_at | 9130401M01Rik | 3 |
| 1416069_at | Pfkp | 3 |
| 1419458_at | Rgnef | 3 |
| 1421682_a_at | Gm3417 /// Gm3448 /// Tcte3 | 3 |
| 1426266_s_at | Zbtb8os | 3 |
| 1429557_at | Mcm8 | 3 |
| 1438563_s_at | Mrps24 | 3 |
| 1449519_at | Gadd45a | 3 |
| 1417713_at | Eif2s2 | 3 |
| 1424328_s_at | 3200002M19Rik | 3 |
| 1423969_at | Nup37 | 3 |
| 1428775_at | 1110008L16Rik | 3 |
| 1424107_at | Kif18a | 3 |
| 1428826_at | Nr6a1 | 3 |
| 1449708_s_at | Chek1 | 3 |
| 1456412_a_at | Inpp5k | 3 |
| 1425373_a_at | LOC100047604 /// Psmg2 | 3 |
| 1444115_at | --- | 3 |
| 1449106_at | Gpx3 | 3 |
| 1449036_at | Rnf128 | 3 |
| 1428654_at | 1200016B10Rik | 3 |
| 1435245_at | Gls2 | 3 |
| 1439988_at | Mrpl15 | 3 |
| 1452086_at | Trmt5 | 3 |
| 1421061_at | Guca1a | 3 |
| 1436543_at | Gtpbp10 | 3 |
| 1424351_at | Wfdc2 | 3 |
| 1417920_at | Amn | 3 |
| 1431182_at | Hspa8 /// LOC624853 /// LOC641192 | 3 |
| 1459137_at | Pml | 3 |
| 1427025_at | Mtmr7 | 3 |
| 1436568_at | Jam2 | 3 |
| 1449502_at | Dazl | 3 |
| 1449675_at | Ccnb1 /// Gm4870 /// Gm5593 /// Gm8416 /// LOC635091 | 3 |
| 1453133_at | Slc25a31 | 3 |
| 1438816_at | Ahctf1 | 3 |
| 1429343_at | LOC100046433 /// Rbmxl2 | 3 |
| 1427271_at | Zbtb44 | 3 |
| 1416748_a_at | Mre11a | 3 |
| 1424458_at | Kdm4c | 3 |
| 1424522_at | Heatr1 | 3 |
| 1431865_a_at | Zfp819 | 3 |
| 1427094_at | Pole2 | 3 |
| 1433547_s_at | Nudcd1 | 3 |
| 1416030_a_at | Mcm7 | 3 |
| 1424212_at | 9430023L20Rik | 3 |
| 1437180_at | Snrnp48 | 3 |
| 1418318_at | Rnf128 | 3 |
| 1423748_at | Pdk1 | 3 |
| 1451477_at | Gm13139 | 3 |
| 1455991_at | Ccbl2 | 3 |
| 1418514_at | Mtf2 | 3 |
| 1436322_a_at | 2810001A02Rik | 3 |
| 1438076_at | --- | 3 |
| 1434665_at | Aga | 3 |
| 1449328_at | Ly75 | 3 |
| 1452795_at | Fcf1 | 3 |
| 1416794_at | Atl2 | 3 |
| 1423862_at | Plekhf2 | 3 |
| 1451491_at | Bcdin3d | 3 |
| 1417640_at | Cd79b | 3 |
| 1428357_at | 2610019F03Rik | 3 |
| 1418538_at | Kdelr3 | 3 |
| 1420918_at | Sgk3 | 3 |
| 1427427_at | Ryr3 | 3 |
| 1435826_at | Rad18 | 3 |
| 1455337_at | Fgd4 | 3 |
| 1428628_at | 1190002A17Rik | 3 |
| 1448816_at | Ptgis | 3 |
| 1448212_at | Psmg2 | 3 |
| 1438168_x_at | Ddx39 | 3 |
| 1447557_at | Rabif | 3 |
| 1428550_at | Ydjc | 3 |
| 1433479_at | Ubr7 | 3 |
| 1453782_at | Ankrd33b | 3 |
| 1448894_at | Akr1b8 | 3 |
| 1426411_a_at | Strbp | 3 |
| 1434882_at | Mtdh | 3 |
| 1455896_a_at | Kcnk1 | 3 |
| 1429552_at | Wdr16 | 3 |
| 1417276_at | Tulp2 | 3 |
| 1448573_a_at | Ceacam10 | 3 |
| 1435924_at | Tfb1m | 3 |
| 1426225_at | Rbp4 | 3 |
| 1419896_at | Tet1 | 3 |
| 1434851_s_at | Crb3 | 3 |
| 1448896_at | Pigf | 3 |
| 1423848_at | Mphosph6 | 3 |
| 1428326_s_at | Hrsp12 | 3 |
| 1449981_a_at | Nat2 | 3 |
| 1431429_a_at | Arl4a | 3 |
| 1419363_a_at | Mrpl35 | 3 |
| 1423122_at | Avpi1 | 3 |
| 1453599_at | Trim71 | 3 |
| 1460227_at | Timp1 | 3 |
| 1417019_a_at | Cdc6 | 3 |
| 1429593_at | Slc38a2 | 3 |
| 1426931_s_at | D19Bwg1357e | 3 |
| 1431428_a_at | Nosip | 3 |
| 1445368_at | --- | 3 |
| 1452364_at | Suz12 | 3 |
| 1452754_at | Creld2 | 3 |
| 1423534_at | Pdcd2 | 3 |
| 1437133_x_at | Akr1b3 | 3 |
| 1442183_at | --- | 3 |
| 1427953_at | Fanci | 3 |
| 1435782_at | Gm12617 | 3 |
| 1460017_at | Gm5595 | 3 |
| 1434734_at | Rad54b | 3 |
| 1423481_at | Riok2 | 3 |
| 1419495_at | Immp2l | 3 |
| 1449109_at | Socs2 | 3 |
| 1435891_x_at | 2610021A01Rik | 3 |
| 1440195_at | Serbp1 | 3 |
| 1425646_at | BC016495 | 3 |
| 1424749_at | Wdfy1 | 3 |
| 1423289_a_at | 1810029B16Rik | 3 |
| 1452823_at | Gstk1 | 3 |
| 1449231_at | Zfp296 | 3 |
| 1452777_a_at | Nub1 | 3 |
| 1456758_at | 9930017N22Rik | 3 |
| 1424713_at | Calml4 | 3 |
| 1448639_a_at | Spata5 | 3 |
| 1434726_at | Tmem146 | 3 |
| 1429262_at | Rassf6 | 3 |
| 1439094_at | Cltc | 3 |
| 1442593_at | --- | 3 |
| 1416530_a_at | LOC100045567 /// Pnp | 3 |
| 1418242_at | Faf1 | 3 |
| 1418292_at | Asna1 | 3 |
| 1429054_at | Mrpl47 | 3 |
| 1437236_a_at | Zfp110 | 3 |
| 1441788_s_at | Dkc1 | 3 |
| 1453106_a_at | Rnmt | 3 |
| 1419656_at | Slc25a36 | 3 |
| 1434037_s_at | Kat2b | 3 |
| 1449749_s_at | Tfb1m | 3 |
| 1438546_x_at | Gm5529 /// Slc25a5 | 3 |
| 1456673_at | --- | 3 |
| 1416071_at | Ddx18 | 3 |
| 1420497_a_at | Cebpz | 3 |
| 1424058_at | Prrc1 | 3 |
| 1450928_at | LOC100045546 | 3 |
| 1460229_at | Stag3 | 3 |
| 1423800_at | Dars | 3 |
| 1434951_at | Armc8 | 3 |
| 1450889_at | Hltf | 3 |
| 1418248_at | Gla | 3 |
| 1419397_at | Pola1 | 3 |
| 1432466_a_at | Apoe | 3 |
| 1448931_at | F2rl1 | 3 |
| 1418820_s_at | Zcchc10 | 3 |
| 1421054_at | Xpo4 | 3 |
| 1449661_at | Suz12 | 3 |
| 1420433_at | Taf7l | 3 |
| 1424906_at | Pqlc3 | 3 |
| 1450677_at | Chek1 | 3 |
| 1451386_at | Blvrb | 3 |
| 1453072_at | Gpr160 | 3 |
| 1428671_at | 2200002D01Rik | 3 |
| 1444797_at | 8030474K03Rik | 3 |
| 1416070_a_at | Ddx18 | 3 |
| 1418543_s_at | Ccdc43 | 3 |
| 1420719_at | Tex15 | 3 |
| 1448690_at | Kcnk1 | 3 |
| 1433978_at | 4930430F08Rik | 3 |
| 1450156_a_at | Hmmr | 3 |
| 1459888_at | LOC545261 | 3 |
| 1429810_at | Rictor | 3 |
| 1435169_at | A930001N09Rik | 3 |
| 1437935_at | 4930486G11Rik | 3 |
| 1428757_at | Aasdhppt | 3 |
| 1441891_x_at | Elovl7 | 3 |
| 1438558_x_at | Foxq1 | 3 |
| 1437694_at | Zfp809 | 3 |
| 1437612_at | --- | 3 |
| 1455735_at | Ap1s3 | 3 |
| 1423767_at | Dpy30 | 3 |
| 1418572_x_at | Tnfrsf12a | 3 |
| 1438161_s_at | Rfc4 | 3 |
| 1416973_at | Nhp2l1 | 3 |
| 1417454_at | Cul4b | 3 |
| 1418462_at | Exosc9 | 3 |
| 1428226_at | Ngdn | 3 |
| 1433724_at | D15Ertd621e | 3 |
| 1448564_at | Cib1 | 3 |
| 1452831_s_at | Ppat | 3 |
| 1448480_at | Nip7 | 3 |
| 1452637_a_at | Bola1 | 3 |
| 1455196_s_at | AA987161 | 3 |
| 1456642_x_at | S100a10 | 3 |
| 1416715_at | Gjb3 | 3 |
| 1418515_at | Mtf2 | 3 |
| 1428214_at | Tomm7 | 3 |
| 1428288_at | Klf9 | 3 |
| 1429734_at | 4632434I11Rik | 3 |
| 1450496_a_at | Ska1 | 3 |
| 1452605_at | Thnsl1 | 3 |
| 1416229_at | Rfk | 3 |
| 1424759_at | Arrdc4 | 3 |
| 1434835_at | Wapal | 3 |
| 1438071_at | Pms1 | 3 |
| 1445322_x_at | --- | 3 |
| 1448948_at | Rag1ap1 | 3 |
| 1460370_at | Top1mt | 3 |
| 1418710_at | Cd59a | 3 |
| 1431784_a_at | Rpf1 | 3 |
| 1431377_at | Atxn10 | 3 |
| 1418704_at | S100a13 | 3 |
| 1426447_at | Nup35 | 3 |
| 1427882_at | Dnttip2 | 3 |
| 1432227_at | --- | 3 |
| 1418805_at | Sct | 3 |
| 1439207_at | Pnma5 | 3 |
| 1428781_at | Dmkn | 3 |
| 1434949_at | Armc8 | 3 |
| 1460454_at | Glod5 | 3 |
| 1433831_at | Dcaf17 | 3 |
| 1434340_at | Gm6293 /// Uqcr10 | 3 |
| 1444106_at | D330012F22Rik | 3 |
| 1418086_at | Ppp1r14a | 3 |
| 1438921_at | Atr | 3 |
| 1450791_at | Nppb | 3 |
| 1453119_at | Otud1 | 3 |
| 1427770_a_at | Slc2a3 | 3 |
| 1448256_at | Gosr1 | 3 |
| 1436203_a_at | 1110059G02Rik | 3 |
| 1437762_at | Rab39 | 3 |
| 1438036_x_at | Fam82a1 | 3 |
| 1441939_x_at | 2410003I16Rik | 3 |
| 1423947_at | 1110008P14Rik | 3 |
| 1446309_at | --- | 3 |
| 1433466_at | AI467606 | 3 |
| 1421749_at | Lin28 | 3 |
| 1416061_at | Tbc1d15 | 3 |
| 1416368_at | Gsta4 | 3 |
| 1419494_a_at | Tpd52 | 3 |
| 1427197_at | Atr | 3 |
| 1432016_a_at | Idh3a | 3 |
| 1438291_x_at | Rpl37 | 3 |
| 1448163_at | Gnpda1 | 3 |
| 1448330_at | Gstm1 | 3 |
| 1455959_s_at | Gclc | 3 |
| 1426425_at | Sugt1 | 3 |
| 1430341_at | Nudt5 | 3 |
| 1433953_at | Zfp277 | 3 |
| 1436803_a_at | Ndufb9 | 3 |
| 1450735_at | Pno1 | 3 |
| 1416439_at | Dctpp1 | 3 |
| 1424465_at | Ccdc58 | 3 |
| 1425562_s_at | Trnt1 | 3 |
| 1426379_at | Eif4b | 3 |
| 1433543_at | Anln | 3 |
| 1435232_x_at | Mrpl15 | 3 |
| 1417506_at | Gmnn | 3 |
| 1448651_at | Nudt5 | 3 |
| 1451352_s_at | Mta3 | 3 |
| 1456341_a_at | Klf9 | 3 |
| 1433565_at | Prpf38a | 3 |
| 1439648_at | Anln | 3 |
| 1416239_at | Ass1 | 3 |
| 1424629_at | Brca1 | 3 |
| 1424775_at | Oas1a | 3 |
| 1434240_at | 4632434I11Rik | 3 |
| 1417216_at | Pim2 | 3 |
| 1418709_at | Cox7a1 | 3 |
| 1429619_a_at | 8430406I07Rik | 3 |
| 1431055_a_at | Snx10 | 3 |
| 1432500_at | 2410006F04Rik | 3 |
| 1436323_at | 2810001A02Rik | 3 |
| 1448819_at | Eif2s2 | 3 |
| 1426290_at | Dimt1 | 3 |
| 1427032_at | Herc4 | 3 |
| 1434419_s_at | Tardbp | 3 |
| 1418791_at | Sh3gl2 | 3 |
| 1443962_at | Tfdp2 | 3 |
| 1455099_at | Mogat2 | 3 |
| 1429207_at | 5730408K05Rik | 3 |
| 1449107_at | Nudt4 | 3 |
| 1450761_s_at | Rims2 | 3 |
| 1450896_at | Arhgap5 | 3 |
| 1453683_a_at | Cep55 | 3 |
| 1453571_at | Depdc6 | 3 |
| 1455948_x_at | Matn3 | 3 |
| 1460514_s_at | Ascl2 | 3 |
| 1436456_at | Slc38a9 | 3 |
| 1424630_a_at | Brca1 | 3 |
| 1429943_at | Ctbs | 3 |
| 1440177_at | Grik3 | 3 |
| 1453080_at | Apol7a | 3 |
| 1449195_s_at | Cxcl16 | 3 |
| 1429254_at | Aqp11 | 3 |
| 1452995_at | Nudt17 | 3 |
| 1453165_at | Mettl4 | 3 |
| 1417421_at | S100a1 | 3 |
| 1422072_a_at | Gstm6 | 3 |
| 1454885_at | Zfp141 | 3 |
| 1421852_at | Kcnk5 | 3 |
| 1456625_at | Aasdhppt | 3 |
| 1427490_at | Abcb7 | 3 |
| 1425674_a_at | Ssu72 | 3 |
| 1426592_a_at | Fbxo22 | 3 |
| 1441921_x_at | Esrrb | 3 |
| 1454682_at | A430005L14Rik | 3 |
| 1456383_at | Rsl1d1 | 3 |
| 1422476_at | Ifi30 | 3 |
| 1429376_s_at | Anapc10 | 3 |
| 1427820_at | --- | 3 |
| 1438016_at | Dkc1 | 3 |
| 1450722_at | Nup50 | 3 |
| 1450987_a_at | 2310004I24Rik | 3 |
| 1453067_at | Apitd1 | 3 |
| 1424321_at | Rfc4 | 3 |
| 1450864_at | Calm3 | 3 |
| 1424296_at | Gclc | 3 |
| 1419606_a_at | Tnnt1 | 3 |
| 1428570_at | Ccnc | 3 |
| 1452859_at | 1200016B10Rik | 3 |
| 1452972_at | Ttc32 | 3 |
| 1455065_x_at | Gm8615 /// Gnpda1 | 3 |
| 1455338_at | A4galt | 3 |
| 1445597_s_at | Pla2g16 | 3 |
| 1437756_at | Gimap9 | 3 |
| 1448445_at | Acp6 | 3 |
| 1428502_at | Actr6 | 3 |
| 1428634_at | Twistnb | 3 |
| 1436562_at | Ddx58 | 3 |
| 1430623_s_at | Obfc2a | 3 |
| 1437658_a_at | Snhg1 | 3 |
| 1450647_at | Hps3 | 3 |
| 1425767_a_at | Six4 | 3 |
| 1439140_at | Ttll8 | 3 |
| 1452009_at | Ttc39b | 3 |
| 1426593_a_at | Fbxo22 | 3 |
| 1442618_at | --- | 3 |
| 1433815_at | Jakmip1 | 3 |
| 1447823_x_at | Mapk12 | 3 |
| 1449374_at | Pipox | 3 |
| 1428547_at | Nt5e | 3 |
| 1443167_at | --- | 3 |
| 1434545_x_at | Bola2 | 3 |
| 1441845_at | Caps2 | 3 |
| 1425141_at | Lactb2 | 3 |
| 1421277_at | LOC630963 /// Spna1 | 3 |
| 1441982_at | Taf7 | 3 |
| 1435119_at | --- | 3 |
| 1421998_at | Tor3a | 3 |
| 1429693_at | Dab2 | 3 |
| 1417814_at | Pla2g5 | 3 |
| 1439269_x_at | Mcm7 | 3 |
| 1458374_at | C79407 | 3 |
| 1428810_at | 2700097O09Rik | 3 |
| 1430474_a_at | Mtch2 | 3 |
| 1437565_a_at | Gnl2 /// LOC633966 | 3 |
| 1459811_at | Mtf2 | 3 |
| 1448029_at | Tbx3 | 3 |
| 1422430_at | Fignl1 | 2 |
| 1415930_a_at | Map1lc3b | 2 |
| 1430542_a_at | Slc25a5 | 2 |
| 1451044_at | Sip1 | 2 |
| 1454888_at | Pfdn4 | 2 |
| 1431292_a_at | Twf2 | 2 |
| 1438932_at | Rasgrp2 | 2 |
| 1430117_a_at | Zfp64 | 2 |
| 1448977_at | Tcfap2c | 2 |
| 1457304_at | D13Ertd787e | 2 |
| 1417872_at | Fhl1 | 2 |
| 1417343_at | Fxyd6 | 2 |
| 1458679_a_at | Tatdn1 | 2 |
| 1415754_at | Polr2f | 2 |
| 1417657_s_at | Dnajc2 | 2 |
| 1418516_at | Mtf2 | 2 |
| 1424732_s_at | Tmem192 | 2 |
| 1434884_at | Mtdh | 2 |
| 1435698_at | Rictor | 2 |
| 1438163_x_at | Rhbdd2 | 2 |
| 1416312_at | Rars | 2 |
| 1422579_at | Hspe1 | 2 |
| 1434109_at | Sh3bgrl2 | 2 |
| 1448319_at | Akr1b3 | 2 |
| 1451505_at | Chchd5 | 2 |
| 1417096_at | Rrp15 | 2 |
| 1424136_a_at | LOC433064 /// Ppih | 2 |
| 1427339_at | Slc30a2 | 2 |
| 1434543_a_at | Bola2 | 2 |
| 1450710_at | Jarid2 | 2 |
| 1450971_at | Gadd45b | 2 |
| 1438980_x_at | Pm20d1 | 2 |
| 1450026_a_at | B3gnt2 | 2 |
| 1453257_at | Agpat5 | 2 |
| 1424327_at | 3200002M19Rik | 2 |
| 1426459_s_at | AW549877 | 2 |
| 1428869_at | Nolc1 | 2 |
| 1429169_at | Rbm3 | 2 |
| 1447703_x_at | Zfp593 | 2 |
| 1451026_at | Ftsj3 | 2 |
| 1452063_at | Zbtb8a | 2 |
| 1435618_at | Pnma2 | 2 |
| 1418792_at | Sh3gl2 | 2 |
| 1416626_at | Pla2g1b | 2 |
| 1419029_at | Ero1l | 2 |
| 1424500_at | Utp6 | 2 |
| 1456951_at | --- | 2 |
| 1458355_x_at | Wdr92 | 2 |
| 1438223_at | --- | 2 |
| 1432023_a_at | Slc17a9 | 2 |
| 1451527_at | Pcolce2 | 2 |
| 1437093_at | Dnaic1 | 2 |
| 1424393_s_at | Adhfe1 | 2 |
| 1424292_at | Depdc1a | 2 |
| 1450036_at | Sgk3 | 2 |
| 1451882_a_at | Fgf8 | 2 |
| 1432590_at | 4930573O21Rik | 2 |
| 1418565_at | Serbp1 | 2 |
| 1435403_at | 1700007L15Rik | 2 |
| 1445214_at | --- | 2 |
| 1419524_at | Tph1 | 2 |
| 1439274_at | Prune2 | 2 |
| 1441229_at | D230019N24Rik | 2 |
| 1431525_at | 9130002K18Rik | 2 |
| 1457127_at | Defb42 | 2 |
| 1426953_at | Hmgxb4 | 2 |
| 1439464_s_at | Tex10 | 2 |
| 1418388_s_at | Mphosph8 | 2 |
| 1458629_at | --- | 2 |
| 1428090_at | Ptcd3 | 2 |
| 1446464_at | Psme4 | 2 |
| 1416416_x_at | Gstm1 | 2 |
| 1416810_at | Mea1 | 2 |
| 1420808_at | Gm6768 /// Ncoa4 | 2 |
| 1423728_at | Eif3l | 2 |
| 1448033_at | Tatdn1 | 2 |
| 1450387_s_at | Ak3l1 /// LOC100047616 | 2 |
| 1450865_s_at | Mrps24 | 2 |
| 1426749_at | Prmt3 | 2 |
| 1431506_s_at | Ppih | 2 |
| 1456019_at | Cwf19l2 | 2 |
| 1457034_at | D14Abb1e | 2 |
| 1416014_at | Abce1 | 2 |
| 1419352_at | l7Rn6 | 2 |
| 1434030_at | Gtpbp10 | 2 |
| 1416031_s_at | Mcm7 | 2 |
| 1418578_at | Dgka | 2 |
| 1429375_at | Anapc10 | 2 |
| 1434132_at | E430025E21Rik | 2 |
| 1435017_at | Cisd3 | 2 |
| 1448135_at | Atf4 | 2 |
| 1448436_a_at | Irf1 | 2 |
| 1452682_at | Fam122b | 2 |
| 1455673_at | Alkbh2 | 2 |
| 1418919_at | Sgol1 | 2 |
| 1423806_at | Ints4 | 2 |
| 1450425_a_at | 2700062C07Rik | 2 |
| 1452184_at | Ndufb9 | 2 |
| 1452983_at | Cep57 | 2 |
| 1419493_a_at | Tpd52 | 2 |
| 1433957_at | C030048B08Rik | 2 |
| 1420548_a_at | 2310008H09Rik | 2 |
| 1424520_at | 2010305A19Rik | 2 |
| 1448306_at | Nfkbia | 2 |
| 1417394_at | Klf4 | 2 |
| 1419614_at | Pla2g12b | 2 |
| 1422507_at | Cstb | 2 |
| 1451369_at | Commd5 | 2 |
| 1439403_x_at | Rlim | 2 |
| 1460369_at | BC003267 | 2 |
| 1460682_s_at | Ceacam1 /// Ceacam2 | 2 |
| 1453392_at | Ttc39b | 2 |
| 1422041_at | Pilrb1 | 2 |
| 1422704_at | Gyk | 2 |
| 1455087_at | D7Ertd715e | 2 |
| 1424230_at | Exoc6 | 2 |
| 1428153_at | Mrps10 | 2 |
| 1434016_at | Znrf2 | 2 |
| 1447977_x_at | Gm14430 /// Gm14434 /// OTTMUSG00000016609 | 2 |
| 1453468_at | 4930430F08Rik | 2 |
| 1454247_a_at | Gpa33 | 2 |
| 1424902_at | Plxdc1 | 2 |
| 1456619_at | Liph | 2 |
| 1454235_a_at | Ing5 | 2 |
| 1445147_at | Psme4 | 2 |
| 1456661_at | --- | 2 |
| 1420357_s_at | Xlr3a /// Xlr3b /// Xlr3c | 2 |
| 1447975_a_at | LOC545261 | 2 |
| 1458099_at | --- | 2 |
| 1424705_at | Rbmx2 | 2 |
| 1416209_at | Glud1 | 2 |
| 1416345_at | Timm8a1 | 2 |
| 1416793_at | Atl2 | 2 |
| 1417598_a_at | Fxr1 | 2 |
| 1434767_at | C79407 | 2 |
| 1437943_s_at | Mea1 | 2 |
| 1442015_at | 2500002B13Rik | 2 |
| 1418036_at | Prim2 | 2 |
| 1416756_at | Dnajb1 | 2 |
| 1422965_at | Agtrap | 2 |
| 1416512_at | Nubp2 | 2 |
| 1423879_at | D030056L22Rik | 2 |
| 1425815_a_at | Hmmr | 2 |
| 1426001_at | Eomes | 2 |
| 1434370_s_at | Faf1 | 2 |
| 1434732_x_at | Tomm7 | 2 |
| 1439510_at | Sgol1 | 2 |
| 1441942_x_at | Snupn | 2 |
| 1450860_at | Lap3 | 2 |
| 1451211_a_at | Lgtn | 2 |
| 1437237_x_at | Zfp110 | 2 |
| 1437370_at | Sgol2 | 2 |
| 1443232_at | Vax2os2 | 2 |
| 1444178_at | Gm9895 | 2 |
| 1425108_a_at | Smagp | 2 |
| 1448244_at | Lypla1 | 2 |
| 1450204_a_at | Mynn | 2 |
| 1460193_at | St13 | 2 |
| 1423373_at | Rpp30 | 2 |
| 1426313_at | Bre | 2 |
| 1433838_at | Dars2 | 2 |
| 1437519_x_at | Hagh /// LOC100044022 | 2 |
| 1449966_s_at | Cab39l | 2 |
| 1416376_at | Tmem97 | 2 |
| 1428519_at | 2610528E23Rik | 2 |
| 1433514_at | Etnk1 | 2 |
| 1434544_at | Bola2 | 2 |
| 1438239_at | Mid1 | 2 |
| 1448783_at | Slc7a9 | 2 |
| 1449699_s_at | C330027C09Rik | 2 |
| 1452580_a_at | Mrpl21 | 2 |
| 1457078_at | Zfp473 | 2 |
| 1459842_x_at | Nubp2 | 2 |
| 1432177_a_at | Mnat1 | 2 |
| 1442296_at | Wdr75 | 2 |
| 1419761_a_at | Gabpb1 | 2 |
| 1428638_at | Efhc2 | 2 |
| 1452008_at | Ttc39b | 2 |
| 1416596_at | Slc44a4 | 2 |
| 1423524_at | Mastl | 2 |
| 1438310_at | --- | 2 |
| 1447541_s_at | Itgae | 2 |
| 1447831_s_at | Mtmr7 | 2 |
| 1422247_a_at | Uty | 2 |
| 1427327_at | Pilra | 2 |
| 1418227_at | Orc2l | 2 |
| 1452590_a_at | Gm9780 /// Plac9 | 2 |
| 1456324_at | Zfp748 | 2 |
| 1434746_at | Mga | 2 |
| 1447119_at | --- | 2 |
| 1421912_at | Slc23a1 | 2 |
| 1423405_at | Timp4 | 2 |
| 1433486_at | Clcn3 | 2 |
| 1424041_s_at | C1s /// LOC100044326 | 2 |
| 1456296_at | 5830418K08Rik | 2 |
| 1418147_at | Tcfap2c | 2 |
| 1423611_at | Alpl | 2 |
| 1445881_at | 2310035P21Rik | 2 |
| 1418215_at | Mep1b | 2 |
| 1420092_at | Morc3 | 2 |
| 1450474_at | Serpinb9c | 2 |
| 1437286_x_at | 1110020G09Rik | 2 |
| 1451511_at | Hibch | 2 |
| 1417566_at | Abhd5 | 2 |
| 1450292_a_at | Gm15529 /// Hormad1 /// LOC675573 | 2 |
| 1441075_at | Nostrin | 2 |
| 1449269_at | F5 | 2 |
| 1417761_at | Apoa4 | 2 |
| 1437367_at | Bat1a | 2 |
| 1422460_at | Mad2l1 | 2 |
| 1438074_at | 2210010C17Rik | 2 |
| 1424355_a_at | Sin3b | 2 |
| 1449506_a_at | Eef1d | 2 |
| 1428963_at | Rwdd2a | 2 |
| 1431123_s_at | Dnajc8 | 2 |
| 1434637_x_at | Sin3b | 2 |
| 1416115_at | Orc3l | 2 |
| 1416311_s_at | Gm5366 /// Tuba3a /// Tuba3b | 2 |
| 1416859_at | Fkbp3 | 2 |
| 1418372_at | Adsl | 2 |
| 1426213_at | Imp4 | 2 |
| 1432444_a_at | Eapp | 2 |
| 1442027_at | Nbeal1 | 2 |
| 1448307_at | Psmg1 | 2 |
| 1450970_at | Got1 | 2 |
| 1428228_at | Pgm3 | 2 |
| 1438993_a_at | Atp6v1d | 2 |
| 1447999_x_at | Gapdh /// Gm10358 /// Gm2076 /// Gm2308 /// LOC100044981 /// LOC100045908 /// LOC100047352 /// LOC100048329 | 2 |
| 1416172_at | Pes1 | 2 |
| 1416667_at | Ebp | 2 |
| 1427253_s_at | Suz12 | 2 |
| 1429817_at | Sohlh2 | 2 |
| 1449171_at | Ttk | 2 |
| 1451746_a_at | Atg12 | 2 |
| 1452852_at | Twistnb | 2 |
| 1455174_at | Rps19bp1 | 2 |
| 1416371_at | Apod | 2 |
| 1416702_at | Serpini1 | 2 |
| 1424436_at | Gart | 2 |
| 1425542_a_at | Ppp2r5c | 2 |
| 1442917_at | Gm11627 | 2 |
| 1460057_at | Gdf3 | 2 |
| 1422650_a_at | Riok3 | 2 |
| 1438520_at | Slc25a36 | 2 |
| 1449731_s_at | Nfkbia | 2 |
| 1452047_at | Cacybp | 2 |
| 1454214_a_at | Znhit6 | 2 |
| 1460354_a_at | Mrpl13 | 2 |
| 1460698_a_at | Sec11c | 2 |
| 1426312_at | Bre | 2 |
| 1436677_at | 1810032O08Rik | 2 |
| 1416517_at | Pnpla6 | 2 |
| 1417250_at | Rlim | 2 |
| 1421951_at | Lhx1 | 2 |
| 1438160_x_at | Slco4a1 | 2 |
| 1455351_at | 2610101N10Rik | 2 |
| 1458163_at | BC066028 | 2 |
| 1460330_at | Anxa3 | 2 |
| 1427505_a_at | Cradd | 2 |
| 1448488_at | Mrps5 | 2 |
| 1460600_at | AA414768 | 2 |
| 1425837_a_at | Ccrn4l /// LOC100047134 | 2 |
| 1428821_at | Agpat2 | 2 |
| 1416850_s_at | Cisd1 | 2 |
| 1417141_at | Igtp | 2 |
| 1419253_at | Mthfd2 | 2 |
| 1455456_a_at | Timm50 | 2 |
| 1417445_at | Ndc80 | 2 |
| 1427930_at | Pdxk | 2 |
| 1418907_at | F5 | 2 |
| 1423766_at | Pak1ip1 | 2 |
| 1429812_at | 2610002D18Rik | 2 |
| 1430165_at | Stk17b | 2 |
| 1436492_x_at | 6430514M23Rik | 2 |
| 1442528_at | Xpo4 | 2 |
| 1443961_at | Gm4340 | 2 |
| 1443377_at | Adam1a | 2 |
| 1453617_at | Zranb3 | 2 |
| 1419095_a_at | Apom | 2 |
| 1452218_at | Ccdc117 | 2 |
| 1449461_at | Rbp7 | 2 |
| 1447802_x_at | --- | 2 |
| 1440815_x_at | Gm8126 /// Gm8267 /// LOC624112 | 2 |
| 1453310_at | Ppil6 | 2 |
| 1456512_at | Pdzrn4 | 2 |
| 1455355_at | G2e3 | 2 |
| 1437540_at | Mcoln3 | 2 |
| 1447691_x_at | --- | 2 |
| 1422605_at | Ppp1r1a | 2 |
| 1439103_at | Cdc73 | 2 |
| 1449641_at | Adk | 2 |
| 1450256_at | Cer1 | 2 |
| 1452987_at | Taf1d | 2 |
| 1453988_a_at | Ide | 2 |
| 1440125_at | A530054K11Rik | 2 |
| 1419223_a_at | Dtna | 2 |
| 1439109_at | Ccdc68 | 2 |
| 1438612_a_at | Clps | 2 |
| 1429055_at | 4930506M07Rik | 2 |
| 1450956_at | LOC100048724 /// Scd3 | 2 |
| 1415917_at | Mthfd1 | 2 |
| 1450714_at | Azin1 | 2 |
| 1417192_at | Tomm70a | 2 |
| 1419351_a_at | l7Rn6 | 2 |
| 1426676_s_at | Tomm70a | 2 |
| 1452935_at | Gm5918 | 2 |
| 1417777_at | Ptgr1 | 2 |
| 1422016_a_at | Cenph | 2 |
| 1423082_at | Derl1 | 2 |
| 1424624_at | 2900011O08Rik | 2 |
| 1428092_at | Cdc5l | 2 |
| 1449855_s_at | Uchl3 /// Uchl4 | 2 |
| 1453210_at | 5730507C01Rik | 2 |
| 1418217_at | Nme7 | 2 |
| 1418369_at | Prim1 | 2 |
| 1430075_at | Sf3b3 | 2 |
| 1439439_x_at | Eef1d | 2 |
| 1419298_at | Pon3 | 2 |
| 1428666_at | Nars | 2 |
| 1438321_x_at | Fam63a | 2 |
| 1448495_at | Tsta3 | 2 |
| 1452061_s_at | Strbp | 2 |
| 1455643_s_at | Tsr1 | 2 |
| 1455832_a_at | Umps | 2 |
| 1433104_at | Pus3 | 2 |
| 1416067_at | Ifrd1 | 2 |
| 1417403_at | Elovl6 | 2 |
| 1455488_at | Haus6 | 2 |
| 1416386_a_at | M6pr | 2 |
| 1417966_at | Mrpl39 | 2 |
| 1419203_at | Gm16516 | 2 |
| 1419513_a_at | Ect2 | 2 |
| 1425788_a_at | Echdc2 | 2 |
| 1426426_at | Mak16 | 2 |
| 1426521_at | D230025D16Rik | 2 |
| 1427153_at | Bckdhb | 2 |
| 1438530_at | Tfpi | 2 |
| 1439440_x_at | Twf2 | 2 |
| 1448626_at | Cdk5rap1 | 2 |
| 1451523_a_at | Mif4gd | 2 |
| 1416413_at | Ctsj | 2 |
| 1446164_at | Tgs1 | 2 |
| 1454703_x_at | Snhg1 | 2 |
| 1420624_a_at | Vamp8 | 2 |
| 1420720_at | LOC100044234 /// Nptx2 | 2 |
| 1424072_at | 2010107G23Rik | 2 |
| 1428194_at | Usp9x | 2 |
| 1428968_at | Cep57 | 2 |
| 1448845_at | Rpp25 | 2 |
| 1449914_at | Ribc1 | 2 |
| 1437435_at | 1700061G19Rik | 2 |
| 1427956_at | Pcgf1 | 2 |
| 1459817_at | --- | 2 |
| 1417611_at | Tmem37 | 2 |
| 1434241_at | Wdr67 | 2 |
| 1424231_s_at | Exoc6 | 2 |
| 1434666_at | LOC100048247 | 2 |
| 1438992_x_at | Atf4 | 2 |
| 1440916_at | 2510049J12Rik | 2 |
| 1425466_at | Senp2 | 2 |
| 1428972_at | Tctex1d2 | 2 |
| 1418186_at | Gstt1 | 2 |
| 1425709_at | Rnf17 | 2 |
| 1453252_at | Dus4l | 2 |
| 1417360_at | Mlh1 | 2 |
| 1440929_at | Ggnbp2 | 2 |
| 1419457_at | Rgnef | 2 |
| 1420439_at | Tsx | 2 |
| 1440066_at | --- | 2 |
| 1449746_s_at | Glipr1 | 2 |
| 1421301_at | Zic2 | 2 |
| 1421278_s_at | LOC630963 /// Spna1 | 2 |
| 1419749_at | Trdmt1 | 2 |
| 1454967_at | A930001N09Rik | 2 |
| 1452257_at | Bdh1 | 2 |
| 1424706_at | Zfp51 | 2 |
| 1438441_at | Id4 | 2 |
| 1429871_at | Hmmr | 2 |
| 1453775_at | Rictor | 2 |
| 1450037_at | Usp9x | 2 |
| 1424118_a_at | Spc25 | 2 |
| 1418656_at | Lsm5 | 2 |
| 1422576_at | Atxn10 | 2 |
| 1449061_a_at | Prim1 | 2 |
| 1454047_a_at | 2410017P07Rik | 2 |
| 1417052_at | Psmb3 | 2 |
| 1418035_a_at | Prim2 | 2 |
| 1427090_at | Zbed4 | 2 |
| 1435140_at | Ide | 2 |
| 1443858_at | 9230105E10Rik | 2 |
| 1444644_x_at | Tatdn1 | 2 |
| 1452598_at | Gins1 | 2 |
| 1423884_at | Cirh1a | 2 |
| 1424858_at | L2hgdh | 2 |
| 1426844_a_at | Pdcd2l | 2 |
| 1430875_a_at | Pak1ip1 | 2 |
| 1452838_at | Ddx10 | 2 |
| 1460429_at | Cdc5l | 2 |
| 1429213_at | 2310030N02Rik | 2 |
| 1438339_at | Fancd2 | 2 |
| 1448953_at | Blm | 2 |
| 1455213_at | Tmsb15b1-Tmsb15b2 /// Tmsb15b2 | 2 |
| 1416569_at | Actl6a | 2 |
| 1416575_at | Cdc45 | 2 |
| 1416958_at | Nr1d2 | 2 |
| 1417791_a_at | Zfml | 2 |
| 1417921_at | 2610029G23Rik | 2 |
| 1424105_a_at | Pttg1 | 2 |
| 1425006_a_at | Vrk1 | 2 |
| 1436174_at | Atad2 | 2 |
| 1452247_at | Fxr1 | 2 |
| 1453018_at | Nvl | 2 |
| 1453064_at | Etaa1 | 2 |
| 1417053_at | Phb | 2 |
| 1423316_at | Tmem39a | 2 |
| 1428084_at | Krr1 | 2 |
| 1438769_a_at | Thyn1 | 2 |
| 1420707_a_at | Traip | 2 |
| 1448809_at | Cse1l | 2 |
| 1449295_at | Sap30bp | 2 |
| 1455576_at | Rinl | 2 |
| 1428004_at | Snrnp25 | 2 |
| 1439208_at | Chek1 | 2 |
| 1439519_at | Slc34a3 | 2 |
| 1443979_at | A830021K08Rik | 2 |
| 1452721_a_at | Ccdc53 | 2 |
| 1455918_at | Adrb3 | 2 |
| 1456548_at | Mtdh | 2 |
| 1418225_at | Orc2l | 2 |
| 1418571_at | Tnfrsf12a | 2 |
| 1420139_s_at | Krr1 | 2 |
| 1423720_a_at | Sar1a | 2 |
| 1435303_at | Taf4b | 2 |
| 1450094_at | Ranbp17 | 2 |
| 1437043_a_at | Fam125a | 2 |
| 1455238_at | Mum1l1 | 2 |
| 1423275_at | Ints6 | 2 |
| 1436683_at | 1700011M02Rik | 2 |
| 1452725_a_at | Rnaseh2a | 2 |
| 1418527_a_at | Sfrs13a | 2 |
| 1424672_at | Dmxl1 | 2 |
| 1438480_a_at | Thyn1 | 2 |
| 1452803_at | Glipr2 | 2 |
| 1419330_a_at | Gpa33 | 2 |
| 1421302_a_at | Gna15 | 2 |
| 1425007_at | Zfp566 | 2 |
| 1425027_s_at | Sft2d2 | 2 |
| 1431388_at | Mphosph10 | 2 |
| 1436796_at | Matr3 | 2 |
| 1437287_at | 1110020G09Rik | 2 |
| 1419368_a_at | Rnf138 | 2 |
| 1459856_at | --- | 2 |
| 1448389_at | Wdr5 | 2 |
| 1437285_at | 1110020G09Rik | 2 |
| 1444530_at | --- | 2 |
| 1447843_at | Psmd13 | 2 |
| 1452596_at | Polr2k | 2 |
| 1430273_at | 2410087M07Rik | 2 |
| 1434917_at | Cobl | 2 |
| 1435987_x_at | 1110059G02Rik | 2 |
| 1448607_at | Nampt | 2 |
| 1435929_at | 9630033F20Rik | 2 |
| 1454008_at | Prmt3 | 2 |
| 1439341_at | Greb1l | 2 |
| 1454946_at | Mybl2 | 2 |
| 1415805_at | Clps | 2 |
| 1441924_x_at | Edn3 | 2 |
| 1423952_a_at | Krt7 | 2 |
| 1424528_at | Cgref1 | 2 |
| 1420310_at | --- | 2 |
| 1419697_at | Cxcl11 | 2 |
| 1437094_x_at | Dnaic1 | 2 |
| 1422716_a_at | Acp1 | 2 |
| 1457410_at | Arhgap5 | 2 |
| 1441444_at | Nbeal1 | 2 |
| 1456359_at | Ppwd1 | 2 |
| 1418110_a_at | Inpp5d | 2 |
| 1455352_at | 2610101N10Rik | 2 |
| AFFX-MURINE_b1_at | --- | 2 |
| 1451377_a_at | Aaas | 2 |
| 1416705_at | Rpe | 2 |
| 1417026_at | Pfdn1 | 2 |
| 1417057_a_at | Lamp3 /// LOC100043444 /// LOC100045251 /// Ppid | 2 |
| 1422301_at | Ftl1 /// Ftl2 /// Gm10116 /// Gm12164 /// LOC434624 /// Mir692-1 | 2 |
| 1448427_at | Ndufa6 | 2 |
| 1448542_at | Bccip | 2 |
| 1449420_at | Pde1b | 2 |
| 1454064_a_at | Rnf138 | 2 |
| 1458303_at | Rnf165 | 2 |
| 1460198_a_at | Psmb3 | 2 |
| 1426496_at | Wdr55 | 2 |
| 1426718_at | Skiv2l2 | 2 |
| 1416751_a_at | Ddx20 | 2 |
| 1428076_s_at | Gm3244 /// Ndufb4 | 2 |
| 1433604_x_at | Aldoa | 2 |
| 1416146_at | Hspa4 | 2 |
| 1419239_at | Zfp54 | 2 |
| 1423522_at | LOC639633 /// Npm3 /// Npm3-ps1 | 2 |
| 1423624_at | Fancl /// LOC100044333 | 2 |
| 1425030_at | Zfp622 | 2 |
| 1430043_at | Ttc19 | 2 |
| 1441504_at | Rbm44 | 2 |
| 1444716_at | Mettl2 | 2 |
| 1451069_at | Pim3 | 2 |
| 1452233_at | Abcc1 | 2 |
| 1418264_at | Cenpk | 2 |
| 1423453_at | Nol12 | 2 |
| 1428458_at | Pop1 | 2 |
| 1444559_at | Phtf2 | 2 |
| 1448287_at | Polr1d | 2 |
| 1424276_at | Snx16 | 2 |
| 1447751_x_at | Dus2l | 2 |
| 1452665_at | Ttc27 | 2 |
| 1418451_at | Gng2 | 2 |
| 1420875_at | Twf1 | 2 |
| 1422630_at | Rad50 | 2 |
| 1424148_a_at | Stap2 | 2 |
| 1424946_a_at | Mtfr1 | 2 |
| 1426256_at | Timm17a | 2 |
| 1429847_a_at | Dcaf17 | 2 |
| 1437696_at | BC049807 | 2 |
| 1448610_a_at | Sod2 | 2 |
| 1450964_a_at | Osbpl9 | 2 |
| 1452266_at | Las1l | 2 |
| 1427541_x_at | Hmmr | 2 |
| 1441098_at | Pnldc1 | 2 |
| 1456200_at | Ipmk | 2 |
| 1429762_a_at | Bbs5 | 2 |
| 1456546_at | 1700097N02Rik | 2 |
| 1456849_at | --- | 2 |
| 1431939_a_at | Mina | 2 |
| 1435181_at | Lin54 | 2 |
| 1446508_at | --- | 2 |
| 1450717_at | Ang | 2 |
| 1436748_at | --- | 2 |
| 1422702_at | Azin1 | 2 |
| 1425538_x_at | Ceacam1 | 2 |
| 1430053_a_at | Ola1 | 2 |
| 1437630_at | Lsg1 | 2 |
| 1447227_at | --- | 2 |
| 1452504_s_at | Ctbs | 2 |
| 1443172_at | Orc1l | 2 |
| 1419959_s_at | Cphx | 2 |
| 1428013_at | 6030458C11Rik | 2 |
| 1426146_a_at | Chpt1 | 2 |
| 1437678_at | Gm1564 | 2 |
| 1450006_at | Gm6768 /// Ncoa4 | 2 |
| 1418495_at | Zc3h8 | 2 |
| 1431345_a_at | Taf1b | 2 |
| 1422567_at | Fam129a | 2 |
| 1424975_at | Siglec5 | 2 |
| 1427658_at | Ctbs | 2 |
| 1432075_a_at | Tekt1 | 2 |
| 1435217_at | Gm7969 | 2 |
| 1439880_at | D630023F18Rik | 2 |
| 1416975_at | Stam2 | 2 |
| 1417401_at | Rai14 | 2 |
| 1422975_at | Mme | 2 |
| 1445787_at | Ccdc162 | 2 |
| 1424974_at | Zfp418 | 2 |
| 1438896_at | Dnajc6 | 2 |
| 1438643_at | Camk1d | 2 |
| 1454799_at | Agpat9 | 2 |
| 1456873_at | Clic5 | 2 |
| 1429270_a_at | Syce2 | 2 |
| 1421020_at | Ranbp17 | 2 |
| 1423636_at | Wdr31 | 2 |
| 1426917_s_at | Scrn3 | 2 |
| 1452880_at | Znhit3 | 2 |
| 1422517_a_at | Znrd1 | 2 |
| 1423772_x_at | Slc25a5 | 2 |
| 1416668_at | Ttc35 | 2 |
| 1442268_a_at | --- | 2 |
| 1452262_at | Grpel2 | 2 |
| 1428215_x_at | Tomm7 | 2 |
| 1456396_at | --- | 2 |
| 1434607_at | Ddx52 | 2 |
| 1448793_a_at | Sdc4 | 2 |
| 1434827_at | Thoc6 | 2 |
| 1426362_at | Tmem144 | 2 |
| 1424070_at | Napg | 2 |
| 1426471_at | Zfp52 | 2 |
| 1426494_at | Rg9mtd3 | 2 |
| 1429248_at | 2810002N01Rik | 2 |
| 1448957_at | Rbpj | 2 |
| 1417659_at | Vps29 | 2 |
| 1418228_at | Nfu1 | 2 |
| 1418530_at | Nup160 | 2 |
| 1428694_at | Mirhg1 | 2 |
| 1436035_at | 3830431G21Rik | 2 |
| 1436874_x_at | Slc25a5 | 2 |
| 1451509_at | Taf9 | 2 |
| 1456282_at | 6720457D02Rik | 2 |
| 1436330_x_at | Gm7072 | 2 |
| 1437423_a_at | Sra1 | 2 |
| 1438021_at | Rbm47 | 2 |
| 1423698_at | Ncaph2 | 2 |
| 1418763_at | Nit2 | 2 |
| 1420850_at | Crnkl1 | 2 |
| 1425627_x_at | Gstm1 | 2 |
| 1429953_at | 2210011C24Rik | 2 |
| 1439091_at | Fancd2 | 2 |
| 1441177_at | --- | 2 |
| 1448205_at | Ccnb1 | 2 |
| 1451417_at | Brca1 | 2 |
| 1452840_at | 1500009L16Rik | 2 |
| 1453169_a_at | Gtf2h1 | 2 |
| 1424927_at | Glipr1 | 2 |
| 1421052_a_at | Sms | 2 |
| 1424392_at | Adhfe1 | 2 |
| 1426353_at | Stat6 | 2 |
| 1448300_at | Mgst3 | 2 |
| 1420603_s_at | Raet1a /// Raet1b /// Raet1c /// Raet1d /// Raet1e | 2 |
| 1427708_a_at | Nf2 | 2 |
| 1430086_at | Chrna9 | 2 |
| 1434151_at | Mettl7a1 | 2 |
| 1441753_at | Rictor | 2 |
| 1450157_a_at | Hmmr | 2 |
| 1429123_at | Rab27a | 2 |
| 1451814_a_at | Htatip2 | 2 |
| 1454036_a_at | Usp15 | 2 |
| 1435597_at | Atad5 | 2 |
| 1447666_x_at | 3200002M19Rik /// Gm6843 | 2 |
| 1428797_at | Setd6 | 2 |
| 1436317_at | Pgap1 | 2 |
| 1451486_at | Slc46a3 | 2 |
| 1451532_s_at | Steap1 | 2 |
| 1453954_a_at | Mrps5 | 2 |
| 1434405_at | Fnip1 | 2 |
| 1450534_x_at | H2-K1 | 2 |
| 1451167_at | Ccdc101 /// LOC100047712 | 2 |
| 1449668_s_at | Fnip1 | 2 |
| 1426255_at | Nefl | 2 |
| 1442223_at | Enah | 2 |
| 1429327_at | Sdccag1 | 2 |
| 1419876_at | 2810449G22Rik | 2 |
| 1423194_at | Arhgap5 | 2 |
| 1439050_at | Gclm | 2 |
| 1443294_at | Cdk12 | 2 |
| 1418686_at | Oas1c | 2 |
| 1421708_a_at | Stat6 | 2 |
| 1451782_a_at | Slc29a1 | 2 |
| 1459890_s_at | 1110008P14Rik | 2 |
| 1426622_a_at | Qpct | 2 |
| 1451436_at | Sbno1 | 2 |
| 1457658_x_at | Anxa4 | 2 |
| 1439415_x_at | Gm5963 /// Rps21 | 2 |
| 1425095_at | BC002059 | 2 |
| 1428680_at | Cds1 | 2 |
| 1448994_at | Sp1 | 2 |
| 1426466_s_at | Rps6kl1 | 2 |
| 1424324_at | Esco1 | 2 |
| 1455913_x_at | Ttr | 2 |
| 1449592_at | Tcf15 | 2 |
| 1440192_at | Ttc39b | 2 |
| 1440924_at | Kif20b | 2 |
| 1419266_at | Nfyb | 2 |
| 1423612_at | Clp1 | 2 |
| 1426451_at | Spg11 | 2 |
| 1429862_at | Pla2g4e | 2 |
| 1432164_a_at | Gcsh | 2 |
| 1438157_s_at | Nfkbia | 2 |
| 1443849_x_at | Urod | 2 |
| 1448447_at | Vps28 | 2 |
| 1449098_a_at | Poli | 2 |
| 1450998_at | Zfp110 | 2 |
| 1452808_at | Snupn | 2 |
| 1454112_a_at | Haus2 | 2 |
| 1454616_at | Ubr7 | 2 |
| 1418439_at | Mrpl42 | 2 |
| 1424641_a_at | Thoc1 | 2 |
| 1428335_a_at | Scfd1 | 2 |
| 1435306_a_at | Kif11 | 2 |
| 1460224_at | Snx2 | 2 |
| 1416610_a_at | Clcn3 | 2 |
| 1417006_at | Commd4 | 2 |
| 1417390_at | Gpn1 | 2 |
| 1419577_at | Fig4 | 2 |
| 1432094_a_at | Ccdc132 | 2 |
| 1440710_at | Zfp706 | 2 |
| 1451042_a_at | Mina | 2 |
| 1416558_at | Melk | 2 |
| 1423129_at | Shoc2 | 2 |
| 1423841_at | Brix1 | 2 |
| 1424712_at | Ahctf1 | 2 |
| 1425058_at | Zfp472 | 2 |
| 1425238_at | Gm6607 | 2 |
| 1426187_a_at | Hax1 | 2 |
| 1426751_s_at | Nup107 | 2 |
| 1428756_at | Aasdhppt | 2 |
| 1429505_at | Nbeal1 | 2 |
| 1457046_s_at | C77370 | 2 |
| 1416994_at | Ttc1 | 2 |
| 1435576_at | AW413774 | 2 |
| 1442058_s_at | Psmc3ip | 2 |
| 1449369_at | Tmprss2 | 2 |
| 1449701_at | Etf1 | 2 |
| 1416910_at | Dnajc15 | 2 |
| 1420425_at | Prdm1 | 2 |
| 1441900_x_at | Hspbap1 | 2 |
| 1452012_a_at | Exosc1 | 2 |
| 1416952_at | Atp6v1d | 2 |
| 1429080_at | Mphosph10 | 2 |
| 1429585_s_at | Mynn | 2 |
| 1434125_at | Utp15 | 2 |
| 1435248_a_at | Btaf1 | 2 |
| 1445391_at | Diap1 | 2 |
| 1453359_at | Exosc1 | 2 |
| 1428389_s_at | Wdr43 | 2 |
| 1436828_a_at | Tpd52l2 | 2 |
| 1438019_at | Ippk | 2 |
| 1448794_s_at | Dnajc2 | 2 |
| 1450745_at | C1galt1 | 2 |
| 1451389_at | Dnajc24 | 2 |
| 1460417_at | AB041803 | 2 |
| 1422470_at | Bnip3 | 2 |
| 1430574_at | Cdkn3 | 2 |
| 1418185_at | Ndufaf3 | 2 |
| 1420776_a_at | Auh | 2 |
| 1421412_at | Gsc | 2 |
| 1423525_at | Mastl | 2 |
| 1423623_at | 2810021B07Rik | 2 |
| 1424843_a_at | Gas5 | 2 |
| 1427931_s_at | Pdxk | 2 |
| 1429795_at | 1700001L05Rik | 2 |
| 1436412_at | --- | 2 |
| 1439131_at | 4932441K18Rik | 2 |
| 1442145_at | Atp13a3 | 2 |
| 1451072_a_at | Rnf4 | 2 |
| 1451512_s_at | Hibch | 2 |
| 1453649_at | C030039L03Rik | 2 |
| 1457021_x_at | Amhr2 | 2 |
| 1426455_at | Cwc27 | 2 |
| 1431086_s_at | Pcmt1 | 2 |
| 1450929_at | Zfp57 | 2 |
| 1460314_s_at | Hist1h3a /// Hist1h3b /// Hist1h3c /// Hist1h3d /// Hist1h3e /// Hist1h3f /// Hist1h3g /// Hist1h3h /// Hist1h3i /// Hist2h3b /// Hist2h3c1 /// Hist2h3c2 | 2 |
| 1416170_at | Trap1 | 2 |
| 1417049_at | Rhd | 2 |
| 1425099_a_at | Arntl | 2 |
| 1425923_at | Mycn | 2 |
| 1442594_at | Ttk | 2 |
| 1429346_a_at | Cep72 | 2 |
| 1439970_at | --- | 2 |
| 1457073_at | --- | 2 |
| 1424766_at | Ercc6l | 2 |
| 1431358_at | 4930547N16Rik | 2 |
| 1418794_at | Cds2 | 2 |
| 1423258_at | Syt9 | 2 |
| 1424153_s_at | Sall4 | 2 |
| 1425210_s_at | Zfp84 | 2 |
| 1429895_at | 2310010G23Rik | 2 |
| 1445519_at | Kcns3 | 2 |
| 1426013_s_at | Plekha4 | 2 |
| 1430604_a_at | Dab2 | 2 |
| 1419461_at | Rpp14 | 2 |
| 1428804_at | Mfap3l | 2 |
| 1435575_at | Kntc1 | 2 |
| 1416240_at | Psmb7 | 2 |
| 1420998_at | Etv5 | 2 |
| 1424097_at | Elovl7 | 2 |
| 1427380_at | Klk1b3 | 2 |
| 1451302_at | 1110012L19Rik | 2 |
| 1452178_at | Parp10 /// Plec | 2 |
| 1456277_at | 7530414M10Rik | 2 |
| 1429477_at | Ncaph2 | 2 |
| 1419805_s_at | Ggps1 /// Gm5630 /// LOC100045315 | 2 |
| 1418345_at | Tnfsf12 /// Tnfsf12-Tnfsf13 /// Tnfsf13 | 2 |
| 1453020_at | 1810048J11Rik | 2 |
| 1420655_at | Pnpla3 | 2 |
| 1455240_x_at | Gm7969 | 2 |
| 1447706_at | --- | 2 |
| 1457566_at | Zfp677 | 2 |
| 1439976_at | Alg11 | 2 |
| 1424676_s_at | Sec14l4 | 2 |
| 1431632_at | 4930526L06Rik | 2 |
| 1429355_at | Tekt5 | 2 |
| 1460038_at | LOC100045707 /// Pou3f1 | 2 |
| 1456554_at | 7530414M10Rik | 2 |
| 1427198_at | BC022960 | 2 |
| 1423860_at | Ptgds | 2 |
| 1419931_at | Abcb7 | 2 |
| 1425996_a_at | Hltf | 2 |
| 1423729_a_at | Rnf181 | 2 |
| 1434801_x_at | Slc25a5 | 2 |
| 1416485_at | Gm10171 /// Timm23 | 2 |
| 1428068_at | Samm50 | 2 |
| 1437046_x_at | Fam63a | 2 |
| 1455800_x_at | Samm50 | 2 |
| 1416595_at | Mrps22 | 2 |
| 1417200_at | Tmem183a | 2 |
| 1423919_at | Haus3 | 2 |
| 1435369_at | Fastkd5 | 2 |
| 1454814_s_at | Gm5081 | 2 |
| 1415984_at | Acadm | 2 |
| 1417193_at | Sod2 | 2 |
| 1417288_at | Plekha2 | 2 |
| 1419173_at | Acy1 | 2 |
| 1423095_s_at | Crbn | 2 |
| 1423620_at | Cenpq | 2 |
| 1424751_at | Abt1 | 2 |
| 1430781_at | Ak7 | 2 |
| 1433959_at | Zmat4 | 2 |
| 1435249_at | Btaf1 | 2 |
| 1448586_at | Hspa14 | 2 |
| 1451258_at | Psca | 2 |
| 1452592_at | Mgst2 | 2 |
| 1454948_at | Usp7 | 2 |
| 1455730_at | Dlgap5 | 2 |
| 1460084_at | --- | 2 |
| 1436723_at | Cenpi | 2 |
| 1438902_a_at | Hsp90aa1 | 2 |
| 1448930_at | 3010026O09Rik | 2 |
| 1418114_at | Rbpj | 2 |
| 1423081_a_at | Tomm20 | 2 |
| 1424434_at | BC024814 | 2 |
| 1424869_at | Dhrs7b | 2 |
| 1428087_at | Dnm1l | 2 |
| 1428754_at | Trmt6 | 2 |
| 1452316_at | Ints12 | 2 |
| 1415826_at | Atp6v1h | 2 |
| 1417400_at | Rai14 | 2 |
| 1417404_at | Elovl6 | 2 |
| 1428331_at | 2210016F16Rik | 2 |
| 1436040_at | Snhg12 | 2 |
| 1438366_x_at | Clcn3 | 2 |
| 1448971_at | 2410022L05Rik | 2 |
| 1450897_at | Arhgap5 | 2 |
| 1451171_at | 2310008H04Rik | 2 |
| 1452314_at | Kif11 | 2 |
| 1452676_a_at | Pnpt1 | 2 |
| 1452917_at | Rfc5 | 2 |
| 1417029_a_at | Trim2 | 2 |
| 1418281_at | Rad51 | 2 |
| 1420021_s_at | Suz12 | 2 |
| 1425129_a_at | Taldo1 | 2 |
| 1425271_at | Psmc3ip | 2 |
| 1436506_a_at | Snhg6 | 2 |
| 1446274_at | Slc16a1 | 2 |
| 1451580_a_at | Ttr | 2 |
| 1451626_x_at | --- | 2 |
| 1417778_at | Zfp35 | 2 |
| 1419927_s_at | Rabif | 2 |
| 1420113_s_at | 2410022L05Rik | 2 |
| 1424333_at | Rg9mtd1 | 2 |
| 1425822_a_at | Dtx1 | 2 |
| 1428593_at | 1700029F09Rik | 2 |
| 1428946_at | Uba6 | 2 |
| 1432181_s_at | Sco2 | 2 |
| 1432332_a_at | Nudt19 | 2 |
| 1433882_at | Cnot10 | 2 |
| 1438937_x_at | Ang | 2 |
| 1440972_at | Nsd1 | 2 |
| 1445897_s_at | Ifi35 | 2 |
| 1449983_a_at | Nqo2 | 2 |
| 1456730_x_at | Actl6a | 2 |
| 1457039_at | Cecr2 | 2 |
| 1419013_at | Gpatch1 | 2 |
| 1420174_s_at | Tax1bp1 | 2 |
| 1428302_at | Mrpl48 | 2 |
| 1452672_at | Thoc5 | 2 |
| 1428551_at | Trmt11 | 2 |
| 1429660_s_at | Smc2 | 2 |
| 1435229_at | Gramd1b | 2 |
| 1436750_a_at | Oxct1 | 2 |
| 1439024_at | Bag4 | 2 |
| 1450402_at | Med1 | 2 |
| 1432459_a_at | Zbtb32 | 2 |
| 1449306_at | Hsf2 | 2 |
| 1434452_x_at | Eif2a | 2 |
| 1459756_at | Cnot10 | 2 |
| 1416062_at | Tbc1d15 | 2 |
| 1420362_a_at | Bik | 2 |
| 1425220_x_at | LOC100038935 | 2 |
| 1433536_at | Lrp11 | 2 |
| 1450668_s_at | Hspe1 | 2 |
| 1455775_at | --- | 2 |
| 1452655_at | Zdhhc2 | 2 |
| 1425116_a_at | Spnb4 | 2 |
| 1429049_at | Nuak2 | 2 |
| 1445708_x_at | 3110021A11Rik | 2 |
| 1439783_at | C330018D20Rik | 2 |
| 1454831_at | Foxn2 | 2 |
| 1423260_at | Id4 /// LOC100045546 | 2 |
| 1449216_at | Itgae | 2 |
| 1434372_at | AW112010 | 2 |
| 1419142_at | Ctsr | 2 |
| 1420335_at | Dmc1 | 2 |
| 1451552_at | Lipt1 | 2 |
| 1419529_at | Il23a | 2 |
| 1434808_at | Palb2 | 2 |
| 1421078_at | Tcf23 | 2 |
| 1424953_at | BC021614 | 2 |
| 1447873_x_at | Bid | 2 |
| 1453092_at | Crct1 | 2 |
| 1439295_x_at | Gm5595 | 2 |
| 1419193_a_at | Gmfg | 2 |
| 1437118_at | Usp7 | 2 |
| 1453727_at | Esf1 | 2 |
| 1431633_x_at | 4930526L06Rik | 2 |
| 1431213_a_at | Gm3579 | 2 |
| 1429900_at | 5330406M23Rik | 2 |
| 1453159_at | Efhc1 | 2 |
| 1426278_at | Ifi27l2a | 2 |
| 1421333_a_at | Mynn | 2 |
| 1458325_x_at | Bmf | 2 |
| 1456864_at | --- | 2 |
| 1454144_a_at | Ccnc | 2 |
| 1443489_at | --- | 2 |
| 1431644_a_at | Ica1 | 2 |
| 1442954_at | --- | 2 |
| 1459989_at | --- | 2 |
| 1439221_s_at | Cd40 | 2 |
| 1421217_a_at | Lgals9 | 2 |
| 1448101_s_at | Trim27 | -2 |
| AFFX-b-ActinMur/M12481_3_at | Actb | -2 |
| 1436722_a_at | Actb | -2 |
| 1448170_at | Siah2 | -2 |
| 1416790_a_at | Gm5806 /// Gm9855 /// Tdg | -2 |
| 1423066_at | Dnmt3a | -2 |
| 1423189_at | 6720456B07Rik | -2 |
| 1425550_a_at | Prkar1a | -2 |
| 1418896_a_at | Rpn2 | -2 |
| 1423956_at | LOC100046746 /// Smap1 | -2 |
| 1424028_at | Chtf8 | -2 |
| 1426685_a_at | Cnot6 | -2 |
| 1428293_at | Bod1 | -2 |
| 1429514_at | Ppap2b | -2 |
| 1434400_at | Tgif2 | -2 |
| 1434486_x_at | Ugp2 | -2 |
| 1449056_at | E330009J07Rik | -2 |
| 1450729_at | Hs2st1 | -2 |
| 1452253_at | Crim1 | -2 |
| 1452910_at | Bcor | -2 |
| 1452952_at | 9030418K01Rik | -2 |
| 1456930_at | Camsap1 | -2 |
| 1415827_a_at | Serp1 | -2 |
| 1424101_at | Hnrnpl | -2 |
| 1436715_s_at | Cdipt | -2 |
| 1452836_at | Lpin2 | -2 |
| 1455598_at | Usp30 | -2 |
| 1415708_at | Tug1 | -2 |
| 1428783_at | Prkar2a | -2 |
| 1429601_x_at | Mthfd2l | -2 |
| 1439478_at | Acot2 | -2 |
| 1440831_at | Bach1 | -2 |
| 1449929_at | Dynlt3 | -2 |
| 1451067_at | Sgta | -2 |
| 1457751_at | Rsf1 | -2 |
| 1417599_at | Cd276 | -2 |
| 1417907_at | Ube2l3 | -2 |
| 1421389_a_at | Eif2ak4 | -2 |
| 1423053_at | Arf4 | -2 |
| 1423297_at | Add3 | -2 |
| 1423897_at | Rnf187 | -2 |
| 1424863_a_at | Hipk2 | -2 |
| 1427078_at | Snx19 | -2 |
| 1436406_at | --- | -2 |
| 1437115_at | Phrf1 | -2 |
| 1438267_x_at | Rnps1 | -2 |
| 1448923_at | Prkra | -2 |
| 1449633_s_at | Nt5c3l | -2 |
| 1451019_at | Ctsf | -2 |
| 1451356_at | Anp32e | -2 |
| 1451495_at | Wac | -2 |
| 1452120_at | Naa40 | -2 |
| 1436560_at | Kif3a | -2 |
| 1437235_x_at | Lpp | -2 |
| 1442124_at | --- | -2 |
| 1448569_at | Mlec | -2 |
| 1453134_at | Pik3ca | -2 |
| 1416036_at | Fkbp1a | -2 |
| 1419819_s_at | Sec63 | -2 |
| 1433796_at | Endod1 | -2 |
| 1447278_at | Cep164 | -2 |
| 1448578_at | Pafah1b1 | -2 |
| 1415726_at | Ankrd17 | -2 |
| 1418144_a_at | Pip5k1a | -2 |
| 1423086_at | Npc1 | -2 |
| 1423893_x_at | Apbb1 | -2 |
| 1424874_a_at | Ptbp1 | -2 |
| 1425940_a_at | Ssbp3 | -2 |
| 1428573_at | Chn2 | -2 |
| 1433625_at | Bat2l | -2 |
| 1436758_at | Hdac4 | -2 |
| 1439619_at | Tcf12 | -2 |
| 1448976_at | Tfip11 | -2 |
| 1460692_at | Ehmt2 | -2 |
| 1421853_at | Psen1 | -2 |
| 1424369_at | Psmf1 | -2 |
| 1433774_x_at | Cog1 | -2 |
| 1439490_at | Nr2c1 | -2 |
| 1449404_at | Pip4k2a | -2 |
| 1426681_at | Unk | -2 |
| 1454985_at | Ambra1 | -2 |
| 1428141_at | Gga2 | -2 |
| 1433541_a_at | Ubap2l | -2 |
| 1434039_at | Appbp2 | -2 |
| 1434233_at | 2610030H06Rik | -2 |
| 1439012_a_at | Dck | -2 |
| 1460578_at | Fgd5 | -2 |
| 1460690_at | Fam195b | -2 |
| 1426738_at | Dgkz | -2 |
| 1428449_at | Gtf3c2 | -2 |
| 1434556_at | Tmem170b | -2 |
| 1437546_at | Dnajc14 | -2 |
| 1439388_s_at | Bcar1 | -2 |
| 1455508_at | Slc35e2 | -2 |
| 1456956_at | Ikzf2 | -2 |
| 1423165_a_at | Mta2 | -2 |
| 1417155_at | Mycn | -2 |
| 1417279_at | Itpr1 | -2 |
| 1418844_at | Alg9 | -2 |
| 1431792_a_at | LOC100047492 /// Stk11ip | -2 |
| 1435473_at | Gm347 | -2 |
| 1454730_at | Tapt1 | -2 |
| 1457935_at | --- | -2 |
| 1434027_at | Rcan3 | -2 |
| 1425344_at | Narf | -2 |
| 1434676_at | Mtmr9 | -2 |
| 1437375_at | Rfx3 | -2 |
| 1437532_at | Rnf216 | -2 |
| 1437542_at | Ikzf2 | -2 |
| 1447788_s_at | Tspyl3 | -2 |
| 1449876_at | Prkg1 | -2 |
| 1434650_at | Pogz | -2 |
| 1452916_at | Wbp7 | -2 |
| 1417630_at | Mknk1 | -2 |
| 1435559_at | Myo6 | -2 |
| 1453059_at | 2310046A06Rik | -2 |
| 1435344_at | Tfdp2 | -2 |
| 1443337_at | --- | -2 |
| 1448340_at | Tmem30a | -2 |
| 1452368_at | Bcr | -2 |
| 1452759_s_at | Ppfibp1 | -2 |
| 1437632_at | Med14 | -2 |
| 1428937_at | Atp2b1 | -2 |
| 1437492_at | Mkx | -2 |
| 1437547_s_at | Dnajc14 | -2 |
| 1455109_at | Tbl1xr1 | -2 |
| 1418772_at | BC016423 | -2 |
| 1418648_at | Egln3 | -2 |
| 1447901_x_at | Sfi1 | -2 |
| 1451860_a_at | Trim30 | -2 |
| 1453101_at | Klhl25 | -2 |
| 1454646_at | Tcp11l2 | -2 |
| 1437434_a_at | Wls | -2 |
| 1417030_at | Tmem206 | -2 |
| 1423286_at | Cbln1 | -2 |
| 1426856_at | Hsdl2 | -2 |
| 1447878_s_at | Fgfrl1 /// LOC100046239 | -2 |
| 1450417_a_at | Rps20 | -2 |
| 1455161_at | AI504432 | -2 |
| 1425899_a_at | Itsn1 | -2 |
| 1426684_at | Cnot6 | -2 |
| 1423689_a_at | Gpsm1 | -2 |
| 1448886_at | Gata3 | -2 |
| 1427550_at | Peg10 | -2 |
| 1449480_at | Sap18 | -2 |
| 1445843_at | Chd2 | -2 |
| 1433853_at | Mib1 | -2 |
| 1424150_at | Gdpd5 | -2 |
| 1424614_at | Pgap2 | -2 |
| 1434632_at | --- | -2 |
| 1426565_at | Igf1r | -2 |
| 1448095_at | --- | -2 |
| 1428607_at | Araf | -2 |
| 1448852_at | Rgn | -2 |
| 1417312_at | Dkk3 | -2 |
| 1434301_at | Fam84b | -2 |
| 1459896_at | Pogk | -2 |
| 1415849_s_at | Stmn1 | -2 |
| 1424143_a_at | Cdt1 | -2 |
| 1419153_at | 2810417H13Rik | -2 |
| 1427333_s_at | Sfrs15 | -2 |
| 1428306_at | Ddit4 | -2 |
| 1437503_a_at | Shisa5 | -2 |
| 1452856_at | Crebzf | -2 |
| 1460428_at | Ankrd13a | -2 |
| 1415696_at | Sar1a | -2 |
| 1416947_s_at | Acaa1a /// Acaa1b | -2 |
| 1417728_at | Mbd3 | -2 |
| 1433682_at | Arhgef17 | -2 |
| 1449144_at | Gna11 | -2 |
| 1417730_at | Ext1 | -2 |
| 1423730_at | Clptm1l | -2 |
| 1426481_at | Klhl22 | -2 |
| 1438165_x_at | Vat1 | -2 |
| 1448180_a_at | Hn1 | -2 |
| 1455046_a_at | Pogz | -2 |
| 1416428_at | Thap11 | -2 |
| 1424049_at | Lrrc42 | -2 |
| 1426776_at | Wasl | -2 |
| 1435573_at | Otud5 | -2 |
| 1436925_at | Foxn3 | -2 |
| 1437288_at | Impad1 | -2 |
| 1438400_at | 4632411B12Rik | -2 |
| 1438652_x_at | Pigq | -2 |
| 1448376_at | Wrnip1 | -2 |
| 1454874_at | Btbd7 | -2 |
| 1455394_at | Pias4 | -2 |
| 1456054_a_at | Pum1 | -2 |
| 1456375_x_at | Trim27 | -2 |
| 1460349_at | Prr14 | -2 |
| 1460545_at | Thrap3 | -2 |
| 1460709_a_at | Bat5 | -2 |
| 1417567_at | Ctnnbip1 | -2 |
| 1427069_at | Fbxo28 | -2 |
| 1433597_at | Arglu1 | -2 |
| 1436028_at | Tmem33 | -2 |
| 1437724_x_at | Pitpnm1 | -2 |
| 1451284_at | Yipf3 | -2 |
| 1453271_at | Phf14 | -2 |
| 1415995_at | Casp6 | -2 |
| 1421048_a_at | Ypel1 | -2 |
| 1422766_at | Stau1 | -2 |
| 1424643_at | Tcof1 | -2 |
| 1436818_a_at | Msi2 | -2 |
| 1437671_x_at | Prss23 | -2 |
| 1451148_at | LOC100047214 /// Pink1 | -2 |
| 1451971_at | Cul4a | -2 |
| 1452812_at | Lphn1 | -2 |
| 1423141_at | Lipa | -2 |
| 1433937_at | Trp53bp2 | -2 |
| 1434088_at | Zkscan17 | -2 |
| 1436908_at | Pcm1 | -2 |
| 1439006_x_at | Fam70a | -2 |
| 1443240_at | --- | -2 |
| 1444543_at | --- | -2 |
| 1448144_at | Hnrnpab | -2 |
| 1448462_at | Gm5806 /// Gm9855 /// Tdg | -2 |
| 1454681_at | Esrp1 | -2 |
| 1455078_at | Ssh2 | -2 |
| 1455855_x_at | Hnrnpab | -2 |
| 1460700_at | Stat3 | -2 |
| 1428253_at | Chmp2b | -2 |
| 1442007_at | Zmym5 | -2 |
| 1453412_a_at | Sec14l1 | -2 |
| 1454644_at | Fam40a | -2 |
| 1423047_at | Tollip | -2 |
| 1447787_x_at | Gjc1 | -2 |
| 1415688_at | Ube2g1 | -2 |
| 1417609_at | Ube2a | -2 |
| 1430012_at | 1110050K14Rik | -2 |
| 1434066_at | Gtf3c1 | -2 |
| 1452036_a_at | Tmpo | -2 |
| 1452438_s_at | LOC100046932 /// Taf4a | -2 |
| 1456731_x_at | Polr3k | -2 |
| 1423370_a_at | Csnk1g2 | -2 |
| 1426850_a_at | Map2k6 | -2 |
| 1427604_a_at | Atp9a | -2 |
| 1429525_s_at | Myo1f | -2 |
| 1436534_at | Trove2 | -2 |
| 1455287_at | Cdk6 | -2 |
| 1429530_a_at | Smpd4 | -2 |
| 1456596_at | Fam70a | -2 |
| 1417386_at | Npepps | -2 |
| 1421139_a_at | Zfp386 | -2 |
| 1422607_at | Etv1 | -2 |
| 1423103_at | Rfx5 | -2 |
| 1423548_s_at | Ergic3 | -2 |
| 1426477_at | Rasa1 | -2 |
| 1434708_at | Vhl | -2 |
| 1435846_x_at | --- | -2 |
| 1442368_at | Kctd12b | -2 |
| 1419097_a_at | Stom | -2 |
| 1458439_a_at | Dzip3 | -2 |
| 1418824_at | Arf6 | -2 |
| 1435981_at | Nav2 | -2 |
| 1420809_a_at | 1500003O03Rik | -2 |
| 1428372_at | St5 | -2 |
| 1452911_at | Spred1 | -2 |
| 1460465_at | A930038C07Rik | -2 |
| 1435488_at | Mthfd2l | -2 |
| 1438385_s_at | Gpt2 | -2 |
| 1453221_at | Gopc | -2 |
| 1417855_at | Fbxl15 | -2 |
| 1417930_at | Nab2 | -2 |
| 1424468_s_at | Phldb1 | -2 |
| 1438462_x_at | Khdrbs1 | -2 |
| 1448891_at | Fcrls | -2 |
| 1450252_at | Onecut1 | -2 |
| 1417305_at | Speg | -2 |
| 1417780_at | Lass4 | -2 |
| 1425214_at | P2ry6 | -2 |
| 1429417_at | Chsy3 | -2 |
| 1449054_a_at | Pcbp4 | -2 |
| 1450973_s_at | Mapkbp1 | -2 |
| 1436953_at | Wipf1 | -2 |
| 1444018_at | B930098A02Rik | -2 |
| 1449298_a_at | Pde1a | -2 |
| 1416863_at | Abhd8 | -2 |
| 1434107_at | Spata2 | -2 |
| 1455419_at | D630045J12Rik | -2 |
| 1415742_at | Aup1 | -2 |
| 1419301_at | Fzd4 | -2 |
| 1426212_s_at | Tmem161a | -2 |
| 1429097_at | Rnf150 | -2 |
| 1436300_at | Dstyk | -2 |
| 1438042_at | Shox2 | -2 |
| 1459948_at | --- | -2 |
| 1426787_at | Sfi1 | -2 |
| 1438154_x_at | 2610002J02Rik | -2 |
| 1455881_at | Ier5l | -2 |
| 1438012_at | Ppm1l | -2 |
| 1418018_at | Cpd | -2 |
| 1443827_x_at | Fam20c | -2 |
| 1457718_at | Srcap | -2 |
| 1421171_at | Adam12 | -2 |
| 1436765_at | Pard3 | -2 |
| 1457094_at | She | -2 |
| 1452159_at | 2310001A20Rik | -2 |
| 1422064_a_at | Zbtb20 | -2 |
| 1435304_at | Sod1 | -2 |
| 1435885_s_at | Itsn1 | -2 |
| 1460741_x_at | D17Wsu92e | -2 |
| 1448760_at | Zfp68 | -2 |
| 1448327_at | Actn2 | -2 |
| 1456603_at | Fam101b | -2 |
| 1424268_at | Smox | -2 |
| 1448680_at | LOC100046946 /// Serpina1c | -2 |
| 1436102_at | Sec22c | -2 |
| 1456500_at | Aph1b | -2 |
| 1439209_at | Tcf12 | -2 |
| 1460394_a_at | Inppl1 | -2 |
| 1416256_a_at | Tubb5 | -2 |
| 1418390_at | Phf21a | -2 |
| 1420614_at | Dynlt3 | -2 |
| 1428768_at | Pan3 | -2 |
| 1433706_a_at | Ptplad1 | -2 |
| 1437032_x_at | Rbm14 | -2 |
| 1448118_a_at | Ctsd | -2 |
| 1449628_s_at | Stard7 | -2 |
| 1451731_at | Abca3 | -2 |
| 1460707_at | Ptp4a2 | -2 |
| 1428422_at | Faf2 | -2 |
| 1434633_at | Crebbp | -2 |
| 1415703_at | Huwe1 | -2 |
| 1422642_at | Cdc42ep3 | -2 |
| 1424638_at | Cdkn1a | -2 |
| 1425801_x_at | Cotl1 | -2 |
| 1428537_at | Csnk1a1 | -2 |
| 1436338_at | --- | -2 |
| 1437211_x_at | Elovl5 | -2 |
| 1438910_a_at | Stom | -2 |
| 1415840_at | Elovl5 | -2 |
| 1416140_a_at | Dhx30 | -2 |
| 1416580_a_at | Stub1 | -2 |
| 1417168_a_at | Usp2 | -2 |
| 1423045_at | Ncbp2 | -2 |
| 1426788_a_at | Ssrp1 | -2 |
| 1427050_at | Txndc16 | -2 |
| 1434294_at | Fam199x | -2 |
| 1434717_at | Cul3 | -2 |
| 1435329_at | Kdm2a | -2 |
| 1437830_x_at | Zbed3 | -2 |
| 1448787_at | LOC100233175 /// Moap1 | -2 |
| 1451970_at | Daglb | -2 |
| 1423799_at | Eif1 | -2 |
| 1435177_a_at | Anapc5 | -2 |
| 1447931_at | Whsc1l1 | -2 |
| 1456111_at | Fam55d | -2 |
| 1423513_at | Uhrf1bp1l | -2 |
| 1427006_at | Rapgef1 | -2 |
| 1429772_at | Plxna2 | -2 |
| 1433711_s_at | LOC100047324 /// Sesn1 | -2 |
| 1435328_at | Cyhr1 | -2 |
| 1450914_at | Ppp1r14b | -2 |
| 1415706_at | Copa | -2 |
| 1417706_at | Naglu | -2 |
| 1418788_at | Tek | -2 |
| 1422466_at | Nxn | -2 |
| 1424236_at | Tbc1d10b | -2 |
| 1426869_at | Boc | -2 |
| 1429246_a_at | Anxa6 | -2 |
| 1431619_a_at | Dtnbp1 | -2 |
| 1434485_a_at | Ugp2 | -2 |
| 1434831_a_at | Foxo3 | -2 |
| 1434845_at | --- | -2 |
| 1436788_at | Acp2 | -2 |
| 1448630_a_at | Sdhc | -2 |
| 1455050_at | E130203B14Rik | -2 |
| 1459861_s_at | Gm12397 /// Kdm2b | -2 |
| 1424442_a_at | Pja2 | -2 |
| 1434009_at | Grlf1 | -2 |
| 1438164_x_at | Flot2 | -2 |
| 1449845_a_at | Ephb4 | -2 |
| 1451359_at | Lpcat1 | -2 |
| 1417327_at | Cav2 | -2 |
| 1433985_at | Abi2 | -2 |
| 1449350_at | Osr1 | -2 |
| 1416401_at | Cd82 | -2 |
| 1424071_s_at | BC018507 | -2 |
| 1428385_at | 08-Mar | -2 |
| 1433754_at | Mbnl2 | -2 |
| 1436584_at | Spry2 | -2 |
| 1454197_a_at | Ccdc86 | -2 |
| 1454732_at | Mtap1s | -2 |
| 1455143_at | Nlgn2 | -2 |
| 1435019_at | Atxn7l3 | -2 |
| 1448567_at | Tmem115 | -2 |
| 1455130_at | Spty2d1 | -2 |
| 1416045_a_at | Smarcb1 | -2 |
| 1416607_at | 4931406C07Rik | -2 |
| 1424634_at | Tceal1 | -2 |
| 1428714_at | Pgrmc2 | -2 |
| 1448510_at | Efna1 | -2 |
| 1451274_at | Ogdh | -2 |
| 1455072_at | Cep350 | -2 |
| 1425419_a_at | Raf1 | -2 |
| 1452768_at | Tex261 | -2 |
| 1459843_s_at | Smad1 | -2 |
| 1415948_at | Creg1 | -2 |
| 1423895_a_at | Celf2 | -2 |
| 1427992_a_at | Rab12 | -2 |
| 1434652_at | Cdc42bpb | -2 |
| 1416339_a_at | Prkcsh | -2 |
| 1416507_at | Cnpy2 | -2 |
| 1420903_at | St6galnac3 | -2 |
| 1421546_a_at | Racgap1 | -2 |
| 1460653_at | Atxn2 /// LOC100047323 | -2 |
| 1422799_at | Bat2 | -2 |
| 1441937_s_at | LOC100047214 /// Pink1 | -2 |
| 1435281_at | Cpt1c | -2 |
| 1442562_at | Zfp827 | -2 |
| 1451761_at | Hoxb4 | -2 |
| 1456331_at | 9130023H24Rik | -2 |
| 1428172_at | Prpf39 | -2 |
| 1434044_at | Repin1 | -2 |
| 1434786_at | Ppp1r12b | -2 |
| 1425125_at | Oit3 | -2 |
| 1419685_at | Upf1 | -2 |
| 1428532_at | Ints7 | -2 |
| 1435010_at | Asb7 | -2 |
| 1458130_at | --- | -2 |
| 1424876_s_at | Spg20 | -2 |
| 1441535_at | Mllt3 | -2 |
| 1422592_at | Ctnnd2 /// LOC100045979 | -2 |
| 1445638_at | --- | -2 |
| 1448818_at | Wnt5a | -2 |
| 1454683_at | Sfrs8 | -2 |
| 1448657_a_at | Dnajb2 | -2 |
| 1455191_x_at | Pip5k1a | -2 |
| 1426994_at | Phlpp1 | -2 |
| 1455145_at | Pcdh19 | -2 |
| 1416077_at | Adm | -2 |
| 1435590_at | Dcun1d5 | -2 |
| 1455686_at | Lcorl | -2 |
| 1415746_at | Cic | -2 |
| 1449249_at | Pcdh7 | -2 |
| 1439799_at | --- | -2 |
| 1424169_at | Tax1bp3 | -2 |
| 1428168_at | Mpzl1 | -2 |
| 1437687_x_at | Fkbp9 | -2 |
| 1423519_at | Fam108c | -2 |
| 1428224_at | Hnrpdl | -2 |
| 1417423_at | Grina | -2 |
| 1451168_a_at | Arhgdia | -2 |
| 1427144_at | Hnrpll | -2 |
| 1447806_s_at | Srpk3 | -2 |
| 1417999_at | Itm2b | -2 |
| 1454680_at | D5Ertd579e | -2 |
| 1449345_at | Ccdc34 | -2 |
| 1416637_at | Slc4a2 | -2 |
| AFFX-b-ActinMur/M12481_5_at | Actb | -2 |
| 1438853_x_at | Ddx54 | -2 |
| 1423049_a_at | Tpm1 | -2 |
| 1456133_x_at | Itgb5 | -2 |
| 1455441_at | Map3k7 | -2 |
| 1454747_a_at | Klhdc3 | -2 |
| 1421315_s_at | Cttn | -2 |
| 1419247_at | Rgs2 | -2 |
| 1438727_at | Dcaf10 | -2 |
| 1421007_at | Col4a6 | -2 |
| 1435504_at | Clip4 | -2 |
| 1434927_at | Hspb7 | -2 |
| 1415963_at | Hnrnph2 | -2 |
| 1424504_at | Rab22a | -2 |
| 1451018_at | Leprotl1 | -2 |
| 1417592_at | Mtor | -2 |
| 1423740_a_at | Rbm10 | -2 |
| 1418397_at | Zfp275 | -2 |
| 1428300_at | Cytsa | -2 |
| 1438478_a_at | Ppp3ca | -2 |
| 1416213_x_at | Surf4 | -2 |
| 1417854_at | Map2k5 | -2 |
| 1420093_s_at | Hnrpdl | -2 |
| 1420376_a_at | H3f3b | -2 |
| 1423872_a_at | Dag1 | -2 |
| 1429050_at | Chic2 | -2 |
| 1430417_s_at | Neurl4 | -2 |
| 1433476_at | C78339 | -2 |
| 1434782_at | Usp42 | -2 |
| 1436877_at | Lrch2 | -2 |
| 1438376_s_at | Trim27 | -2 |
| 1448240_at | Mbtps1 | -2 |
| 1448642_at | Pcbp1 | -2 |
| 1449542_at | Pbx1 | -2 |
| 1452039_a_at | Bap1 | -2 |
| 1455052_a_at | 2410129H14Rik | -2 |
| 1456609_at | Camk2n1 | -2 |
| AFFX-b-ActinMur/M12481_M_at | Actb | -2 |
| 1417666_at | Dnttip1 | -2 |
| 1425042_s_at | Pelp1 | -2 |
| 1428585_at | Actn1 | -2 |
| 1456439_x_at | Mical1 | -2 |
| 1423474_at | Top1 | -2 |
| 1423803_s_at | Gltscr2 | -2 |
| 1428411_at | --- | -2 |
| 1433887_at | Dnajc3 | -2 |
| 1437436_s_at | Grk6 | -2 |
| 1437751_at | Ppargc1a | -2 |
| 1417766_at | Cyb5b | -2 |
| 1424539_at | Ubl4 | -2 |
| 1436596_at | H2afv | -2 |
| 1438602_s_at | Masp1 | -2 |
| 1448191_at | Plk1 | -2 |
| 1448919_at | Cd302 | -2 |
| 1449932_at | Csnk1d | -2 |
| 1454973_at | Atf7ip | -2 |
| 1420326_s_at | Cramp1l | -2 |
| 1424144_at | Cdt1 | -2 |
| 1424215_at | Fundc1 | -2 |
| 1433712_at | AW555464 | -2 |
| 1436495_s_at | Zfp260 | -2 |
| 1450844_at | Stx6 | -2 |
| 1452313_at | 5930416I19Rik | -2 |
| 1416303_at | Litaf | -2 |
| 1427266_at | Pbrm1 | -2 |
| 1441391_at | --- | -2 |
| 1419164_at | Zfp260 | -2 |
| 1425532_a_at | Bin1 | -2 |
| 1426957_at | Trp53bp1 | -2 |
| 1431293_a_at | Cldnd1 | -2 |
| 1433582_at | 1190002N15Rik /// LOC100044725 | -2 |
| 1434021_at | C230096C10Rik | -2 |
| 1436422_at | BC026590 | -2 |
| 1449058_at | Gli1 | -2 |
| 1450388_s_at | Twsg1 | -2 |
| 1457843_at | Lypd6 | -2 |
| 1456741_s_at | Gpm6a | -2 |
| 1426366_at | Eif2c2 | -2 |
| 1435762_at | Pacs1 | -2 |
| 1439235_x_at | Tm2d2 | -2 |
| 1439500_at | Scrn1 | -2 |
| 1455269_a_at | Coro1a | -2 |
| 1424586_at | Ehbp1 | -2 |
| 1433946_at | Zik1 | -2 |
| 1436819_at | 06-Sep | -2 |
| 1442810_x_at | Scn9a | -2 |
| 1443966_at | --- | -2 |
| 1421290_at | Hspb7 | -2 |
| 1452193_a_at | Wasl | -2 |
| 1418774_a_at | Atp7a | -2 |
| 1434274_at | Mycbp2 | -2 |
| 1439498_at | Igdcc3 | -2 |
| 1448698_at | Ccnd1 | -2 |
| 1449553_at | Nkain1 | -2 |
| 1455368_at | Zdhhc3 | -2 |
| 1455381_at | 4921513D23Rik | -2 |
| 1424877_a_at | Alad /// LOC100046072 | -2 |
| 1434832_at | Foxo3 | -2 |
| 1455606_at | N4bp1 | -2 |
| 1431424_at | 2810055G20Rik | -2 |
| 1433672_at | Trmt2b | -2 |
| 1454780_at | Galntl4 | -2 |
| 1457043_at | B3galtl | -2 |
| 1417135_at | Srpk2 | -2 |
| 1435346_at | Ccdc82 | -2 |
| 1453128_at | Lyve1 | -2 |
| 1441970_at | E430010N07Rik | -2 |
| 1453030_at | Msl2 | -2 |
| 1416108_a_at | Tmed3 | -2 |
| 1450673_at | Col9a2 | -2 |
| 1427274_at | Rnf214 | -2 |
| 1433757_a_at | Nisch | -2 |
| 1435656_at | Gmps | -2 |
| 1454742_at | Rasgef1b | -2 |
| 1451115_at | Pias3 | -2 |
| 1452848_at | Tmem181a /// Tmem181b | -2 |
| 1418181_at | Ptp4a3 | -2 |
| 1416714_at | Irf8 | -2 |
| 1435343_at | Dock10 | -2 |
| 1435558_at | Bai2 /// LOC100048816 | -2 |
| 1438254_at | 1110007A13Rik | -2 |
| 1451264_at | Frmd6 | -2 |
| 1435644_at | Sh3pxd2b | -2 |
| 1434887_at | Ncs1 | -2 |
| 1451507_at | Mef2c | -2 |
| 1452750_at | 5530601H04Rik | -2 |
| 1453107_s_at | 4933413G19Rik /// Foxm1 /// Pebp1 | -2 |
| 1455407_at | Zfp236 | -2 |
| 1455428_at | Fam53b | -2 |
| 1451866_a_at | Hgf | -2 |
| 1449544_a_at | Kcnh2 /// LOC100047076 | -2 |
| 1450482_a_at | Pitx2 | -2 |
| 1442095_at | Asxl3 | -2 |
| 1428319_at | Pdlim7 | -2 |
| 1449286_at | Ntng1 | -2 |
| 1444229_at | Nr2f2 | -2 |
| 1429796_at | Kalrn | -2 |
| 1433864_at | Lrp12 | -2 |
| 1434272_at | Cpeb2 | -2 |
| 1435626_a_at | Herpud1 | -2 |
| 1449070_x_at | Apcdd1 | -2 |
| 1452693_at | Dhx35 | -2 |
| 1424306_at | Elovl4 | -2 |
| 1416192_at | Napa | -2 |
| 1429656_at | Rhobtb1 | -2 |
| 1435538_at | 0610030E20Rik | -2 |
| 1455150_at | Hecw2 | -2 |
| 1457273_at | Odz2 | -2 |
| 1455943_at | Zfp451 | -2 |
| 1426889_at | Zfp821 | -2 |
| 1419550_a_at | Stk39 | -2 |
| 1441214_at | Exph5 | -2 |
| 1442903_at | 4732423E21Rik | -2 |
| 1419033_at | 2610018G03Rik | -2 |
| 1436270_at | Cyb5d1 /// Gm6685 | -2 |
| 1437984_x_at | Bat1a | -2 |
| 1450770_at | 3632451O06Rik | -2 |
| 1420997_a_at | Gpi1 | -2 |
| 1434999_at | Suv420h1 | -2 |
| 1436193_at | Man1c1 | -2 |
| 1460571_at | Dicer1 | -2 |
| 1424294_at | Ppp4r1 | -2 |
| 1426473_at | Dnajc9 | -2 |
| 1429093_at | Ddi2 | -2 |
| 1429679_at | Lrrc17 | -2 |
| 1434521_at | Rfx7 | -2 |
| 1435339_at | Kctd15 | -2 |
| 1438416_at | Med16 | -2 |
| 1452545_a_at | Itgb1 | -2 |
| 1418123_at | Unc119 | -2 |
| 1419869_s_at | Hdlbp | -2 |
| 1422730_at | Limd1 | -2 |
| 1423591_at | Fgfr1op2 | -2 |
| 1427245_at | Arfgap1 | -2 |
| 1428646_at | LOC676870 /// Pbx1 | -2 |
| 1435353_a_at | Pisd-ps1 /// Pisd-ps3 | -2 |
| 1436775_a_at | Ankrd17 | -2 |
| 1442757_at | Lrch1 | -2 |
| 1448297_a_at | Tnk2 | -2 |
| 1448661_at | Plcb3 | -2 |
| 1450081_x_at | Gpi1 | -2 |
| 1454609_x_at | 6430527G18Rik | -2 |
| 1454861_at | Txlna | -2 |
| 1456763_at | Mprip | -2 |
| 1460285_at | Itga9 | -2 |
| 1460350_at | Osbp | -2 |
| 1421654_a_at | Lmna | -2 |
| 1428254_at | Purb | -2 |
| 1448850_a_at | Dnajc5 | -2 |
| 1450307_x_at | H2afy3 | -2 |
| 1420711_a_at | Pxmp3 | -2 |
| 1423910_at | Agap3 | -2 |
| 1427149_at | Plekha6 | -2 |
| 1427903_at | Phpt1 | -2 |
| 1428788_at | Pgp | -2 |
| 1436438_s_at | Dcaf5 | -2 |
| 1437142_a_at | Pigo | -2 |
| 1448580_at | Glg1 | -2 |
| 1450814_a_at | Ipo4 | -2 |
| 1452424_at | Lpar4 | -2 |
| 1416156_at | Vcl | -2 |
| 1416937_at | Gabarap | -2 |
| 1417827_at | Ngly1 | -2 |
| 1419256_at | Spnb2 | -2 |
| 1423687_a_at | Man2c1 | -2 |
| 1424778_at | Reep3 | -2 |
| 1433807_at | 6720463M24Rik | -2 |
| 1435630_s_at | Acat2 | -2 |
| 1436513_at | Tanc2 | -2 |
| 1438969_x_at | Dhx30 | -2 |
| 1449026_at | Ifnar1 | -2 |
| 1449942_a_at | Ilk | -2 |
| 1451896_a_at | Cherp | -2 |
| 1418694_at | Kcmf1 | -2 |
| 1419689_at | Gpc6 | -2 |
| 1424532_at | Ylpm1 | -2 |
| 1428596_at | Tbc1d9b | -2 |
| 1434116_at | Cbx2 | -2 |
| 1439565_at | Fam123b | -2 |
| 1460556_at | Micall1 | -2 |
| 1434488_at | Arfrp1 | -2 |
| 1439122_at | Ddx6 | -2 |
| 1448663_s_at | Mvd | -2 |
| 1419499_at | Gpam | -2 |
| 1420934_a_at | Srrm1 | -2 |
| 1435362_at | Foxj3 | -2 |
| 1452660_s_at | Klhl7 | -2 |
| 1452960_at | Scyl3 | -2 |
| 1454668_at | Ubr4 | -2 |
| 1456011_x_at | Acaa1a | -2 |
| 1458943_at | --- | -2 |
| 1460344_at | Pbxip1 | -2 |
| 1435923_at | Ado | -2 |
| 1426530_a_at | Klhl5 | -2 |
| 1452765_at | Slc39a9 | -2 |
| 1423962_at | Wdr26 | -2 |
| 1424954_a_at | Pip5k1c | -2 |
| 1428270_at | Glt8d1 | -2 |
| 1439113_at | 2410018L13Rik /// Gm4983 /// Gm5977 /// Gm9237 /// Gm9282 /// Gm9292 /// Gm9309 /// Gm9312 | -2 |
| 1459108_a_at | Yeats2 | -2 |
| 1416891_at | Numb | -2 |
| 1418081_at | Dnajc30 | -2 |
| 1429065_at | Ikbip | -2 |
| 1460646_at | Csnk2a2 | -2 |
| 1453957_a_at | Igf2bp3 | -2 |
| 1456040_at | Sf3b2 | -2 |
| 1428715_at | Gfpt1 | -2 |
| 1436133_at | Ccdc127 | -2 |
| 1452345_at | Lmod2 | -2 |
| 1450994_at | Rock1 | -2 |
| 1460371_at | Hspa12b | -2 |
| 1428976_at | Tmpo | -2 |
| 1419181_at | Zfp326 | -2 |
| 1435921_at | Atxn1l | -2 |
| 1420619_a_at | Aes | -2 |
| 1424182_at | Acat1 | -2 |
| 1416432_at | Pfkfb3 | -2 |
| 1451046_at | LOC100047651 /// Zfpm1 | -2 |
| 1445420_at | --- | -2 |
| 1453110_at | Poc1b | -2 |
| 1455715_at | LOC100038746 | -2 |
| 1460356_at | Esam | -2 |
| 1416611_at | Scamp2 | -2 |
| 1424824_at | Slain1 | -2 |
| 1428904_at | Ammecr1l | -2 |
| 1439078_at | Klhl4 | -2 |
| 1442348_at | Katnal1 | -2 |
| 1436867_at | Srl | -2 |
| 1440093_at | Ednra | -2 |
| 1416564_at | Sox7 | -2 |
| 1421892_at | St3gal2 | -2 |
| 1429086_at | Gm16136 /// Grhl2 | -2 |
| 1417929_at | Slc7a8 | -2 |
| 1451110_at | Egln1 | -2 |
| 1434130_at | Lhfpl2 | -2 |
| 1435198_at | Gm5817 | -2 |
| 1443793_x_at | Ints1 | -2 |
| 1452992_at | Cdc26 | -2 |
| 1424023_at | Ctu1 | -2 |
| 1455354_at | Dcaf5 | -2 |
| 1456232_at | Gmeb1 | -2 |
| 1459895_at | Ppp4r1l | -2 |
| 1419004_s_at | Bcl2a1a /// Bcl2a1b /// Bcl2a1d | -2 |
| 1417806_at | Popdc2 | -2 |
| 1424692_at | 2810055F11Rik | -2 |
| 1434451_at | Gm10786 | -2 |
| 1439188_at | Cpsf6 | -2 |
| 1442427_at | --- | -2 |
| 1436309_at | Neto2 | -2 |
| 1420654_a_at | Gbe1 | -2 |
| 1428457_at | Ttpal | -2 |
| 1444606_at | Efna2 | -2 |
| 1438676_at | Mpa2l | -2 |
| 1446525_at | --- | -2 |
| 1436778_at | Cybb | -2 |
| 1419662_at | Ogn | -2 |
| 1446086_s_at | Gli2 | -2 |
| 1453596_at | Id2 | -2 |
| 1435715_x_at | Gm5806 /// Gm9855 /// Tdg | -2 |
| 1425913_a_at | Spats2l | -2 |
| 1419954_s_at | Zfand3 | -2 |
| 1425340_a_at | Ptpra | -2 |
| 1439259_x_at | Abhd4 | -2 |
| 1428528_at | Get4 /// Gm3606 | -2 |
| 1434245_a_at | Cybasc3 | -2 |
| 1436522_at | Map3k3 | -2 |
| 1451988_s_at | Chmp4b /// LOC674706 | -2 |
| 1454612_at | Mex3c | -2 |
| 1418850_at | Epc1 | -2 |
| 1415920_at | Cstf2t | -2 |
| 1416083_at | Zfand5 | -2 |
| 1417964_at | Ap3d1 | -2 |
| 1419945_s_at | Rab2a | -2 |
| 1428346_at | Trafd1 | -2 |
| 1428549_at | Ccdc3 | -2 |
| 1429434_at | Pik3ca | -2 |
| 1417205_at | Kdelr2 | -2 |
| 1456615_a_at | Bptf | -2 |
| 1416493_at | Ddost | -2 |
| 1418067_at | Cfl2 | -2 |
| 1421907_at | Med1 | -2 |
| 1423707_at | Tmem50b | -2 |
| 1424311_at | Carkd | -2 |
| 1429152_at | Zkscan1 | -2 |
| 1434820_s_at | Pkig | -2 |
| 1451073_at | Sppl3 | -2 |
| 1434796_at | Vamp4 | -2 |
| 1452832_s_at | Cds2 | -2 |
| 1417284_at | Mapkap1 | -2 |
| 1429166_s_at | Clmn | -2 |
| 1434087_at | Mthfr | -2 |
| 1435000_at | Gspt1 | -2 |
| 1456072_at | Ppp1r9a | -2 |
| 1438459_x_at | Sfpq | -2 |
| 1426908_at | Galnt7 | -2 |
| 1439060_s_at | Wipi1 | -2 |
| 1428689_at | Tysnd1 | -2 |
| 1446516_at | Bcl7c | -2 |
| 1454929_s_at | Safb | -2 |
| 1423825_at | Wls | -2 |
| 1416038_at | Snd1 | -2 |
| 1426238_at | Bmp1 | -2 |
| 1436489_x_at | C85492 | -2 |
| 1419295_at | Creb3l1 | -2 |
| 1428594_at | Ralgapa1 | -2 |
| 1448530_at | Gmpr /// LOC100045393 | -2 |
| 1438034_at | Fam82b | -2 |
| 1456798_at | 9330118A15Rik | -2 |
| 1433823_at | Ptpdc1 | -2 |
| 1449510_at | Zfp467 | -2 |
| 1457024_x_at | Slc35a2 | -2 |
| 1436678_at | Sgcb | -2 |
| 1419032_at | 2610018G03Rik | -2 |
| 1415753_at | Fam108a | -2 |
| 1416945_at | Ptov1 | -2 |
| 1426215_at | Ddc | -2 |
| 1429233_at | 11-Sep | -2 |
| 1435195_at | Vash1 | -2 |
| 1438082_at | Tmem206 | -2 |
| 1455308_at | Ano6 | -2 |
| 1455539_at | Gm9983 | -2 |
| 1456308_x_at | Trim28 | -2 |
| 1434898_at | Tnrc6a | -2 |
| 1450048_a_at | Idh2 | -2 |
| 1420110_s_at | Zfp334 | -2 |
| 1426160_a_at | Stk16 | -2 |
| 1431057_a_at | Prss23 | -2 |
| 1432673_at | 2300010F08Rik | -2 |
| 1434663_at | 2410129H14Rik | -2 |
| 1437331_a_at | Arf3 | -2 |
| 1451899_a_at | Gtf2ird1 | -2 |
| 1453095_at | Rab10 | -2 |
| 1456424_s_at | Pltp | -2 |
| 1456765_at | Nhsl2 | -2 |
| 1417136_s_at | Srpk2 | -2 |
| 1419027_s_at | Gltp | -2 |
| 1426878_at | Pbrm1 | -2 |
| 1427879_at | 1810031K17Rik | -2 |
| 1433507_a_at | Hmgn2 | -2 |
| 1436448_a_at | Ptgs1 | -2 |
| 1448858_at | Ulk2 | -2 |
| 1417786_a_at | Rgs19 | -2 |
| 1418066_at | Cfl2 | -2 |
| 1433509_s_at | Reep1 | -2 |
| 1434703_at | Extl3 | -2 |
| 1435745_at | 5031439G07Rik | -2 |
| 1436518_at | Usp46 | -2 |
| 1450881_s_at | Gpr137b | -2 |
| 1452166_a_at | Krt10 | -2 |
| 1460398_at | Phf8 | -2 |
| 1440770_at | Bcl2 | -2 |
| 1441139_at | Gm10336 | -2 |
| 1434729_at | Zc4h2 | -2 |
| 1437615_s_at | Vps37c | -2 |
| 1416323_at | Kctd20 | -2 |
| 1422736_at | LOC100045146 /// Ranbp9 | -2 |
| 1424326_at | Lemd2 | -2 |
| 1426587_a_at | Stat3 | -2 |
| 1426877_a_at | Pbrm1 | -2 |
| 1433475_a_at | C78339 | -2 |
| 1445108_at | --- | -2 |
| 1453014_a_at | Sec31a | -2 |
| 1435551_at | Fhod3 | -2 |
| 1419308_at | Invs | -2 |
| 1423083_at | Rab33b | -2 |
| 1423672_at | Ttc30b | -2 |
| 1429302_at | Csnk2a2 | -2 |
| 1429383_at | Csnk1g3 | -2 |
| 1439965_at | Slc43a2 | -2 |
| 1455033_at | Fam102b | -2 |
| 1418985_at | Cttnbp2nl | -2 |
| 1425391_a_at | Osbpl5 | -2 |
| 1426818_at | Arrdc4 | -2 |
| 1436062_at | Arcn1 | -2 |
| 1438419_at | Rbm16 | -2 |
| 1439014_at | 1600021P15Rik | -2 |
| 1448165_at | Casp2 | -2 |
| 1422572_at | Rhog | -2 |
| 1451134_a_at | Tm2d2 | -2 |
| 1426866_at | Chst14 | -2 |
| 1428650_at | Tns1 | -2 |
| 1428653_x_at | Elavl1 | -2 |
| 1427970_at | Zfp689 | -2 |
| 1432591_at | Pappa | -2 |
| 1443833_at | --- | -2 |
| 1429435_x_at | Pik3ca | -2 |
| 1428564_at | Zfp579 | -2 |
| 1434379_at | Mxd4 | -2 |
| 1436217_at | Zfp148 | -2 |
| 1430187_at | Zfp60 | -2 |
| 1416482_at | Ttc3 | -2 |
| 1425083_at | Otor | -2 |
| 1424917_a_at | Wipi1 | -2 |
| 1440870_at | Prdm16 | -2 |
| 1454863_at | Ankrd11 | -2 |
| 1422135_at | Zfp146 | -2 |
| 1453581_at | Cep170 | -2 |
| 1437122_at | Bcl2 | -2 |
| 1438156_x_at | Cpt1a | -2 |
| 1416190_a_at | Sec61a1 | -2 |
| 1458505_at | LOC552901 | -2 |
| 1457446_at | Opcml | -2 |
| 1444974_at | AU023617 | -2 |
| 1437468_x_at | Fbxw11 | -2 |
| 1448479_at | Psmd3 | -2 |
| 1453849_s_at | Hnrnpab | -2 |
| 1421374_a_at | Fxyd1 | -2 |
| 1423205_at | Tm9sf4 | -2 |
| 1423917_a_at | Cttn | -2 |
| 1424769_s_at | Cald1 | -2 |
| 1428108_x_at | Tmcc2 | -2 |
| 1437403_at | Samd5 | -2 |
| 1452774_at | Hnrnpa3 | -2 |
| 1453102_at | Flrt3 | -2 |
| 1419062_at | Epb4.1l3 | -2 |
| 1423961_at | Wdr26 | -2 |
| 1437474_at | Gatad2b | -2 |
| 1452694_at | Ip6k1 | -2 |
| 1423653_at | Atp1a1 | -2 |
| 1424711_at | Tmem2 | -2 |
| 1433926_at | Dync1li2 | -2 |
| 1435609_at | Trp53bp1 | -2 |
| 1437290_at | Impad1 | -2 |
| 1415914_at | Hnrnpab | -2 |
| 1421964_at | Notch3 | -2 |
| 1423037_at | Aplnr | -2 |
| 1426209_at | Strn4 | -2 |
| 1434184_s_at | Map4k4 | -2 |
| 1434207_at | Rnf169 | -2 |
| 1434986_a_at | Sec61a1 | -2 |
| 1437041_at | Sfrs18 | -2 |
| 1437308_s_at | F2r | -2 |
| 1438752_at | A230058F20Rik | -2 |
| 1440817_x_at | LOC100047782 /// Zfp771 | -2 |
| 1448185_at | Herpud1 | -2 |
| 1453285_at | Tmem88 | -2 |
| 1422487_at | Smad4 | -2 |
| 1429251_at | Prdm2 | -2 |
| 1433725_at | Acvr1b | -2 |
| 1435398_at | Stxbp5 | -2 |
| 1436169_at | --- | -2 |
| 1436186_at | E2f8 | -2 |
| 1442883_s_at | Fam108a | -2 |
| 1450053_at | Kif2a | -2 |
| 1454776_at | Ehmt1 | -2 |
| 1416534_at | Dpf2 | -2 |
| 1424619_at | Sf3b4 | -2 |
| 1415974_at | Map2k2 | -2 |
| 1429841_at | Megf10 | -2 |
| 1434389_at | Sos1 | -2 |
| 1436421_s_at | Arpc5l | -2 |
| 1437871_at | LOC100046963 /// Pgm5 | -2 |
| 1439405_x_at | Cntd1 | -2 |
| 1441955_s_at | LOC676674 /// Paip1 | -2 |
| 1449813_at | Zfp30 | -2 |
| 1424341_s_at | Pcdha1 /// Pcdha10 /// Pcdha11 /// Pcdha12 /// Pcdha2 /// Pcdha3 /// Pcdha4 /// Pcdha5 /// Pcdha6 /// Pcdha7 /// Pcdha8 /// Pcdha9 /// Pcdhac1 /// Pcdhac2 | -2 |
| 1431032_at | Agl | -2 |
| 1460173_at | Lasp1 | -2 |
| 1437861_s_at | Prkce | -2 |
| 1451554_a_at | Aph1a | -2 |
| 1455481_at | Ids | -2 |
| 1416124_at | Ccnd2 | -2 |
| 1420834_at | Vamp2 | -2 |
| 1422866_at | Col13a1 | -2 |
| 1428101_at | Rnf38 | -2 |
| 1437471_at | Lrrc45 | -2 |
| 1442174_at | Tspan18 | -2 |
| 1452783_at | Fndc3b | -2 |
| 1455430_at | Rbm33 | -2 |
| 1459622_at | Gm22 | -2 |
| 1460735_at | Svil | -2 |
| 1421043_s_at | Arhgef2 | -2 |
| 1427962_at | Ccdc102a | -2 |
| 1452328_s_at | Pja2 | -2 |
| 1460565_at | Slc41a1 | -2 |
| 1424376_at | Cdc42ep1 | -2 |
| 1426476_at | Rasa1 | -2 |
| 1429987_at | 9930013L23Rik | -2 |
| 1435676_at | Cep164 | -2 |
| 1416866_at | Bet1 | -2 |
| 1441680_at | --- | -2 |
| 1448209_a_at | Slc22a17 | -2 |
| 1418164_at | Stx2 | -2 |
| 1425918_at | --- | -2 |
| 1434690_at | Lclat1 | -2 |
| 1424812_at | BC017158 | -2 |
| 1435244_at | Vav2 | -2 |
| 1453848_s_at | Zbed3 | -2 |
| 1456901_at | Adamts20 | -2 |
| 1449065_at | Acot1 /// LOC100044830 | -2 |
| 1455941_s_at | Map2k5 | -2 |
| 1437473_at | Maf | -2 |
| 1449073_at | Flnc | -2 |
| 1452358_at | Rai2 | -2 |
| 1456663_x_at | Tm2d2 | -2 |
| 1452100_at | Dullard /// LOC100048221 | -2 |
| 1418939_at | Hlx | -2 |
| 1449334_at | Timp3 | -2 |
| 1455256_at | Tnik | -2 |
| 1418265_s_at | Irf2 | -2 |
| 1436082_at | --- | -2 |
| 1458578_at | --- | -2 |
| 1419443_at | Sap18 | -2 |
| 1430697_at | Ammecr1 | -2 |
| 1417540_at | Elf1 | -2 |
| 1452872_at | Ank3 | -2 |
| 1457305_at | Btrc | -2 |
| 1426828_at | 1300018I17Rik | -2 |
| 1435668_at | Fam120b | -2 |
| 1428940_at | Gnaq | -2 |
| 1429197_s_at | Rabgap1l | -2 |
| 1416412_at | Nsmaf | -2 |
| 1436535_at | Trove2 | -2 |
| 1443075_at | --- | -2 |
| 1453512_at | 5830407P18Rik | -2 |
| 1450746_at | Keap1 | -2 |
| 1421477_at | Cplx2 | -2 |
| 1434828_at | Fam102b | -2 |
| 1416419_s_at | Gabarapl1 | -2 |
| 1416457_at | Ddah2 | -2 |
| 1422849_a_at | Pabpn1 | -2 |
| 1426812_a_at | Fam129b | -2 |
| 1434790_a_at | LOC100048056 /// Lta4h | -2 |
| 1454689_at | Srrm1 | -2 |
| 1455719_at | Tubb5 | -2 |
| 1429652_at | Prrc1 | -2 |
| 1435194_at | Hspa4 | -2 |
| 1436179_a_at | Dnajc5 | -2 |
| 1449094_at | Gjc1 | -2 |
| 1452784_at | Itgav | -2 |
| 1438458_a_at | Sfpq | -2 |
| 1455470_x_at | Lasp1 | -2 |
| 1415743_at | Hdac5 | -2 |
| 1415951_at | Fkbp10 | -2 |
| 1415999_at | Hey1 | -2 |
| 1422673_at | Prkd1 | -2 |
| 1423613_at | Ssfa2 | -2 |
| 1433648_at | Spag9 | -2 |
| 1434440_at | Gnai1 | -2 |
| 1435253_at | Rab11b | -2 |
| 1448487_at | Lrrfip1 | -2 |
| 1449041_a_at | Trip6 | -2 |
| 1450393_a_at | Adat3 /// Scamp4 | -2 |
| 1417407_at | Fbxl14 | -2 |
| 1429958_x_at | Haghl | -2 |
| 1460303_at | Nr3c1 | -2 |
| 1416198_at | Th1l | -2 |
| 1418469_at | Nrip1 | -2 |
| 1426817_at | Mki67 | -2 |
| 1437465_a_at | P4hb | -2 |
| 1416872_at | Tspan6 | -2 |
| 1418859_at | Rfxap | -2 |
| 1428343_at | Rcor3 | -2 |
| 1439511_at | Cdk7 | -2 |
| 1447852_x_at | Rilpl1 | -2 |
| 1451434_s_at | Gpatch8 | -2 |
| 1452650_at | Trim62 | -2 |
| 1454806_at | Fam49a | -2 |
| 1454819_at | Plekha8 | -2 |
| 1456698_s_at | Hnrpdl | -2 |
| 1416812_at | Tia1 | -2 |
| 1417493_at | Bmi1 | -2 |
| 1420973_at | Arid5b /// LOC100044968 | -2 |
| 1434423_at | Gulp1 | -2 |
| 1437234_x_at | Prmt2 | -2 |
| 1450519_a_at | Prkaca | -2 |
| 1418577_at | Trim8 | -2 |
| 1429539_at | Bcl2l13 | -2 |
| 1435771_at | Plcb4 | -2 |
| 1448007_at | Baz2b | -2 |
| 1451447_at | Cuedc1 | -2 |
| 1424114_s_at | Lamb1-1 | -2 |
| 1437187_at | E2f7 | -2 |
| 1448413_at | 2410016O06Rik | -2 |
| 1453013_at | Zfp740 | -2 |
| 1455535_at | Sox5 | -2 |
| 1458218_s_at | Pde7a | -2 |
| 1434918_at | Sox6 | -2 |
| 1422789_at | Aldh1a2 | -2 |
| 1423155_at | Sri | -2 |
| 1437298_at | Wipf2 | -2 |
| 1453199_at | Acbd6 | -2 |
| 1428455_at | Col14a1 | -2 |
| 1434185_at | Acaca | -2 |
| 1445518_at | --- | -2 |
| 1451501_a_at | Ghr | -2 |
| 1425510_at | Mark1 | -2 |
| 1429839_a_at | Yaf2 | -2 |
| 1442061_at | Btbd7 | -2 |
| 1420416_at | Sema3a | -2 |
| 1431353_at | Pabpc4l | -2 |
| 1448384_at | Pofut2 | -2 |
| 1418585_at | Ccnh | -2 |
| 1424055_at | Ncoa5 | -2 |
| 1429163_at | Dchs1 | -2 |
| 1434034_at | Cerk /// LOC676420 | -2 |
| 1435739_at | Lats1 | -2 |
| 1436436_at | Cnih4 | -2 |
| 1438802_at | Foxp1 | -2 |
| 1454736_at | Ankrd57 | -2 |
| 1456312_x_at | Gsn | -2 |
| 1420841_at | Ptprf | -2 |
| 1422510_at | Ctdspl | -2 |
| 1420612_s_at | Ptp4a2 | -2 |
| 1425711_a_at | Akt1 | -2 |
| 1435879_at | Akt3 | -2 |
| 1426597_s_at | Iffo2 | -2 |
| 1436269_s_at | Htra2 | -2 |
| 1440383_at | Dclre1b | -2 |
| 1458892_at | 9430047G12Rik | -2 |
| 1416017_at | Copg | -2 |
| 1437753_at | 6230409E13Rik | -2 |
| 1442050_at | Zfp608 | -2 |
| 1443282_at | Prpf38a | -2 |
| 1418192_at | Mnt | -2 |
| 1436382_at | Zbtb12 | -2 |
| 1421072_at | Irx5 | -2 |
| 1447854_s_at | Hist2h2be | -2 |
| 1424194_at | Rcsd1 | -2 |
| 1460684_at | Tm7sf2 | -2 |
| 1426673_at | Cdh3 | -2 |
| 1436054_at | Khnyn | -2 |
| 1440037_at | Pbx1 | -2 |
| 1452657_at | Ap1s2 | -2 |
| 1425981_a_at | Rbl2 | -2 |
| 1448501_at | Tspan6 | -2 |
| 1439101_at | Mylk3 | -2 |
| 1424138_at | Rhbdf1 | -2 |
| 1428643_at | Mgat5 | -2 |
| 1438027_at | --- | -2 |
| 1438878_at | 6430537K16Rik | -2 |
| 1429196_at | Rabgap1l | -2 |
| 1437310_at | Bbs1 | -2 |
| 1438114_x_at | Efs | -2 |
| 1447900_x_at | Entpd4 /// LOC100048085 | -2 |
| 1435327_at | Lpgat1 | -2 |
| 1443790_x_at | 4930414L22Rik | -2 |
| 1434333_a_at | Prkd2 | -2 |
| 1434547_at | Cpd | -2 |
| 1448625_at | Golga2 | -2 |
| 1443952_at | --- | -2 |
| 1458811_at | 9430047L24Rik | -2 |
| 1455901_at | Chpt1 | -2 |
| 1438303_at | Tgfb2 | -2 |
| 1436059_at | Rfx1 | -2 |
| 1451364_at | Polr3gl | -2 |
| 1424783_a_at | Ugt1a1 /// Ugt1a10 /// Ugt1a2 /// Ugt1a5 /// Ugt1a6a /// Ugt1a6b /// Ugt1a7c /// Ugt1a9 | -2 |
| 1440954_at | --- | -2 |
| 1435384_at | Ube2n | -2 |
| 1448221_at | Bat1a | -2 |
| 1422511_a_at | Ogfr | -2 |
| 1435652_a_at | Gnai2 | -2 |
| 1436297_a_at | Grina | -2 |
| 1448908_at | Ppap2b | -2 |
| 1452056_s_at | Ppp3ca | -2 |
| 1453381_at | Rsf1 | -2 |
| 1423030_at | Vcp | -2 |
| 1424466_at | Ipo9 | -2 |
| 1418622_at | Rab2a | -2 |
| 1423394_at | Pcyox1 | -2 |
| 1424145_at | Prr3 | -2 |
| 1435638_at | Gsk3a | -2 |
| 1450711_at | Brd4 | -2 |
| 1417144_at | Tubg1 | -2 |
| 1418101_a_at | Rtn3 | -2 |
| 1423160_at | Spred1 | -2 |
| 1426779_x_at | Dag1 | -2 |
| 1430554_at | Lrig3 | -2 |
| 1433933_s_at | Slco2b1 | -2 |
| 1433986_at | BC024659 | -2 |
| 1437745_at | Chd7 | -2 |
| 1439460_a_at | Arfgap2 | -2 |
| 1441170_a_at | Dab2ip | -2 |
| 1460561_x_at | Sepw1 | -2 |
| 1429296_at | Rab10 | -2 |
| 1437891_at | Frs2 | -2 |
| 1452675_at | Rbm22 | -2 |
| 1460495_s_at | Htra2 | -2 |
| 1425505_at | Mylk | -2 |
| 1451538_at | Sox9 | -2 |
| 1455038_at | 5730471H19Rik | -2 |
| 1416536_at | Mum1 | -2 |
| 1423350_at | Socs5 | -2 |
| 1429727_at | Slc16a9 | -2 |
| 1434281_at | Dda1 | -2 |
| 1434621_at | Tmem204 | -2 |
| 1436180_at | Dnajc5 | -2 |
| 1451458_at | Tmem2 | -2 |
| 1452338_s_at | Itsn1 | -2 |
| 1454723_at | Fam172a | -2 |
| 1417269_at | Cdk9 | -2 |
| 1420713_a_at | Mdfi | -2 |
| 1427228_at | Palld | -2 |
| 1437395_at | Zcchc11 | -2 |
| 1451221_at | BC018507 | -2 |
| 1417438_at | Rdh14 | -2 |
| 1439411_a_at | Xpo7 | -2 |
| 1415794_a_at | Spin1 | -2 |
| 1416759_at | Mical1 | -2 |
| 1426983_at | Fnbp1 | -2 |
| 1448669_at | Dkk3 | -2 |
| 1450883_a_at | Cd36 | -2 |
| 1415766_at | Sec22b | -2 |
| 1420150_at | Spsb1 | -2 |
| 1436809_a_at | Spin1 | -2 |
| 1437526_x_at | Gm6159 /// Hnrnpr | -2 |
| 1455403_at | Manea | -2 |
| 1424424_at | Slc39a1 | -2 |
| 1425508_s_at | Arfrp1 | -2 |
| 1434441_at | 1110018J18Rik | -2 |
| 1416406_at | Pea15a | -2 |
| 1417876_at | Fcgr1 | -2 |
| 1418715_at | Pank1 | -2 |
| 1420772_a_at | Tsc22d3 | -2 |
| 1425231_a_at | Zfp46 | -2 |
| 1429749_at | Sfmbt1 | -2 |
| 1434756_at | 5430421B17 | -2 |
| 1450377_at | LOC640441 /// Thbs1 | -2 |
| 1455008_at | Gna12 | -2 |
| 1418006_at | Zc3h18 | -2 |
| 1438635_x_at | B930041F14Rik | -2 |
| 1425892_a_at | Pnoc | -2 |
| 1435313_at | Cd200r4 | -2 |
| 1452181_at | Ckap4 | -2 |
| 1428681_at | Gm608 | -2 |
| 1429722_at | Zbtb4 | -2 |
| 1434105_at | Epm2aip1 | -2 |
| 1439484_at | Pde7a | -2 |
| 1417927_at | Ddx19a | -2 |
| 1427131_s_at | Lrrc58 | -2 |
| 1429242_at | 1110054O05Rik | -2 |
| 1437057_at | Megf6 | -2 |
| 1439148_a_at | Pfkl | -2 |
| 1452895_at | Fbxo45 | -2 |
| 1434246_at | L3mbtl3 | -2 |
| 1439847_s_at | Klf12 | -2 |
| 1455057_at | Gmps | -2 |
| 1418106_at | Hey2 | -2 |
| 1428246_at | Vps26b | -2 |
| 1429579_at | 6330407I18Rik | -2 |
| 1434967_at | Zswim6 | -2 |
| 1418967_a_at | St7 | -2 |
| 1419668_at | Sgcb | -2 |
| 1434866_x_at | Cpt1a | -2 |
| 1437173_at | S1pr3 | -2 |
| 1451413_at | Cast | -2 |
| 1457009_at | Rhobtb3 | -2 |
| 1419829_a_at | Gab2 | -2 |
| 1439616_at | --- | -2 |
| 1417803_at | 1110032A04Rik | -2 |
| 1454787_at | Zdhhc9 | -2 |
| 1425514_at | Pik3r1 | -2 |
| 1460345_at | Aida | -2 |
| 1438024_at | --- | -2 |
| 1418835_at | Phlda1 | -2 |
| 1427084_a_at | Map4k5 | -2 |
| 1437206_at | Setd5 | -2 |
| 1456480_at | Fry | -2 |
| 1423162_s_at | Spred1 | -2 |
| 1459048_s_at | Zfp142 | -2 |
| 1422752_at | Polr3k | -2 |
| 1437748_at | Fut11 | -2 |
| 1423229_at | Inpp5e | -2 |
| 1426952_at | Arhgap18 | -2 |
| 1452366_at | Csgalnact1 | -2 |
| 1447471_at | --- | -2 |
| 1460363_at | Tnrc6c | -2 |
| 1425567_a_at | Anxa5 | -2 |
| 1448422_at | Tmed4 | -2 |
| 1429942_at | Gmeb1 | -2 |
| 1452182_at | Galnt2 | -2 |
| 1415721_a_at | Nat15 | -2 |
| 1416690_at | Gtpbp2 | -2 |
| 1423334_at | Ergic1 | -2 |
| 1426388_s_at | Ryk | -2 |
| 1426830_a_at | Ahcyl1 | -2 |
| 1433955_at | Brwd1 | -2 |
| 1441894_s_at | Grasp | -2 |
| 1426539_at | Usp11 | -2 |
| 1429104_at | Limd2 | -2 |
| 1433429_at | Pigs | -2 |
| 1456399_at | --- | -2 |
| 1429047_at | Rtf1 | -2 |
| 1460450_at | Ammecr1l | -2 |
| 1416094_at | Adam9 | -2 |
| 1422103_a_at | Stat5b | -2 |
| 1426940_at | Sidt2 | -2 |
| 1427125_s_at | Lrrc41 | -2 |
| 1428982_at | Atad2b | -2 |
| 1435240_at | Baz2b | -2 |
| 1436235_x_at | 4732471D19Rik | -2 |
| 1428779_at | Zbtb41 | -2 |
| 1438214_at | Trps1 | -2 |
| 1460172_at | 5730403B10Rik | -2 |
| 1438211_s_at | Dbp | -2 |
| 1460411_s_at | Pkdcc | -2 |
| 1416503_at | Lxn | -2 |
| 1436849_x_at | Gaa | -2 |
| 1437537_at | Casp9 | -2 |
| 1440411_at | --- | -2 |
| 1449660_s_at | Coro1c | -2 |
| 1451066_at | Mboat7 | -2 |
| 1416454_s_at | Acta2 | -2 |
| 1422551_at | Zkscan3 | -2 |
| 1424039_at | Tmem66 | -2 |
| 1417389_at | Gpc1 | -2 |
| 1417624_at | Nab1 | -2 |
| 1424540_at | Hipk1 | -2 |
| 1434775_at | Pard3 | -2 |
| 1452966_at | Bcl11b | -2 |
| 1430535_at | Tsc22d2 | -2 |
| 1436195_at | BC046404 | -2 |
| 1439845_at | Hp1bp3 | -2 |
| 1454988_s_at | Rab22a | -2 |
| 1460567_at | Rfx7 | -2 |
| 1454794_at | Spast | -2 |
| 1427384_at | Chd6 | -2 |
| 1430030_at | 5330426P16Rik | -2 |
| 1433897_at | AI597468 | -2 |
| 1436921_at | Atp7a | -2 |
| 1459868_x_at | Gm13305 /// Gm2002 /// Il11ra1 /// Il11ra2 | -2 |
| 1451895_a_at | Dhcr24 | -2 |
| 1456778_at | --- | -2 |
| 1434010_at | Fam117b | -2 |
| 1418637_at | Etv3 | -2 |
| 1451435_at | Cux1 | -2 |
| 1423417_at | Smarcc1 | -2 |
| 1425521_at | Paip1 | -2 |
| 1433633_at | Irf2bp2 | -2 |
| 1429590_at | Tacc1 | -2 |
| 1457044_at | Macc1 | -2 |
| 1416246_a_at | Coro1a | -2 |
| 1454893_at | Fam189b | -2 |
| 1460379_at | Hoxb4 | -2 |
| 1416025_at | Fgg | -2 |
| 1436860_at | Senp7 | -2 |
| 1456199_x_at | LOC100045146 /// Ranbp9 | -2 |
| 1458585_at | --- | -2 |
| 1455418_at | --- | -2 |
| 1417987_at | Btd | -2 |
| 1420697_at | Slc15a3 | -2 |
| 1435664_at | Zfp397 | -2 |
| 1449370_at | Sox4 | -2 |
| 1454884_at | Zbtb46 | -2 |
| 1455277_at | Hhip | -2 |
| 1421191_s_at | Gopc | -2 |
| 1433788_at | Nrxn3 | -2 |
| 1439541_at | 4930414L22Rik | -2 |
| 1428990_at | 2310047K21Rik | -2 |
| 1457867_at | Sgpp2 | -2 |
| 1419555_at | Elf5 | -2 |
| 1425281_a_at | Tsc22d3 | -2 |
| 1444767_at | --- | -2 |
| 1445381_at | --- | -2 |
| 1423824_at | Wls | -2 |
| 1436276_at | --- | -2 |
| 1415906_at | Tmsb4x | -2 |
| 1423521_at | Lmnb1 | -2 |
| 1424370_s_at | Psmf1 | -2 |
| 1434824_at | Baz1b | -2 |
| 1456131_x_at | Dag1 | -2 |
| 1434395_at | Man1a2 | -2 |
| 1415812_at | Gsn | -2 |
| 1451634_at | Airn | -2 |
| 1418187_at | Ramp2 | -2 |
| 1457454_at | Usp47 | -2 |
| 1418169_at | Zcchc14 | -2 |
| 1426403_at | Actr1b | -2 |
| 1437462_x_at | Mmp15 | -2 |
| 1420953_at | Add1 | -2 |
| 1426241_a_at | Scmh1 | -2 |
| 1452187_at | Rbm5 | -2 |
| 1454675_at | Thra | -3 |
| 1433723_s_at | Serf2 | -3 |
| 1452649_at | Rtn4 | -3 |
| 1417818_at | Wwtr1 | -3 |
| 1450079_at | Nrk | -3 |
| 1427036_a_at | Eif4g1 | -3 |
| 1420611_at | Prkacb | -3 |
| 1426401_at | Ppp3ca | -3 |
| 1415869_a_at | Trim28 | -3 |
| 1429234_s_at | 11-Sep | -3 |
| 1439264_x_at | Lasp1 | -3 |
| 1436051_at | Myo5a | -3 |
| 1448021_at | Fam46c | -3 |
| 1428936_at | Atp2b1 | -3 |
| 1417968_a_at | Mbd1 | -3 |
| 1426987_at | 5430417L22Rik | -3 |
| 1418539_a_at | Ptpre | -3 |
| 1417005_at | Klc1 | -3 |
| 1433852_at | Kidins220 | -3 |
| 1416189_a_at | Sec61a1 | -3 |
| 1425611_a_at | Cux1 | -3 |
| 1428252_at | Chmp2b | -3 |
| 1438047_at | Zfp384 | -3 |
| 1441645_s_at | Ipo9 | -3 |
| 1455117_at | Mcm9 | -3 |
| 1416813_at | Tia1 | -3 |
| 1423074_at | Lman2 | -3 |
| 1436884_x_at | Ewsr1 | -3 |
| 1438663_at | Bat2l2 | -3 |
| 1448276_at | Tspan4 | -3 |
| 1455871_s_at | Rpl13 /// Tax1bp3 | -3 |
| 1434387_at | Itfg3 | -3 |
| 1437910_at | Tmem39b | -3 |
| 1423278_at | Ptprk | -3 |
| 1433908_a_at | Cttn | -3 |
| 1426746_at | 1810026J23Rik | -3 |
| 1434975_x_at | Pisd-ps3 | -3 |
| 1448335_s_at | Ccni | -3 |
| 1449221_a_at | Rrbp1 | -3 |
| 1450021_at | Ubqln2 | -3 |
| 1454773_at | Rxra | -3 |
| 1438075_at | Fem1c | -3 |
| 1417534_at | Itgb5 | -3 |
| 1426123_a_at | Rrbp1 | -3 |
| 1426454_at | Arhgdib | -3 |
| 1451200_at | Kif1b | -3 |
| 1416332_at | Cirbp | -3 |
| 1423052_at | Arf4 | -3 |
| 1423765_at | Athl1 | -3 |
| 1424895_at | Gpsm2 | -3 |
| 1434063_at | Zfp664 | -3 |
| 1435478_at | Wdr26 | -3 |
| 1442297_at | BB212172 | -3 |
| 1448708_at | Med1 | -3 |
| 1450755_at | Pafah1b2 | -3 |
| 1455768_at | Npc2 | -3 |
| 1460444_at | Arrb1 | -3 |
| 1431226_a_at | Fndc4 | -3 |
| 1434028_at | Arnt2 | -3 |
| 1428630_x_at | Haghl | -3 |
| 1433741_at | Cd38 | -3 |
| 1434427_a_at | Rnf157 | -3 |
| 1426293_at | Zfp790 | -3 |
| 1434481_at | Msl1 | -3 |
| 1448649_at | Enpep | -3 |
| 1452014_a_at | Igf1 | -3 |
| 1447830_s_at | Rgs2 | -3 |
| 1429269_at | BC068157 | -3 |
| 1435293_at | Adam22 | -3 |
| 1438555_x_at | Muc4 | -3 |
| 1439638_at | Erbb2ip | -3 |
| 1448619_at | Dhcr7 | -3 |
| 1421047_at | LOC100046891 /// Smad5 | -3 |
| 1426247_at | Stk24 | -3 |
| 1452221_a_at | Cxxc1 | -3 |
| 1419450_at | Ormdl3 | -3 |
| 1421059_a_at | Alg2 | -3 |
| 1435550_at | Mll2 | -3 |
| 1421818_at | Bcl6 | -3 |
| 1421963_a_at | Cdc25b | -3 |
| 1451533_at | BC022687 | -3 |
| 1429549_at | Col27a1 | -3 |
| 1452232_at | Galnt7 | -3 |
| 1416573_at | Pofut2 | -3 |
| 1435913_at | B4galnt4 | -3 |
| 1424604_s_at | Sumf1 | -3 |
| 1438069_a_at | Rbm5 | -3 |
| 1455180_at | Gcom1 | -3 |
| 1441212_at | Fam92a | -3 |
| 1437181_at | Peli2 | -3 |
| 1442270_at | --- | -3 |
| 1426491_at | Herc2 | -3 |
| 1440275_at | Runx3 | -3 |
| 1446708_at | Hif3a | -3 |
| 1426314_at | Ednrb | -3 |
| 1455886_at | Cbl /// LOC100048010 | -3 |
| 1435464_at | 1110003E01Rik | -3 |
| 1436413_at | Frk | -3 |
| 1460337_at | Sh3kbp1 | -3 |
| 1435608_at | LOC631806 /// Znrf3 | -3 |
| 1448323_a_at | Bgn | -3 |
| 1419066_at | Ier5l | -3 |
| 1423954_at | C3 | -3 |
| 1451520_at | Spg20 | -3 |
| 1417420_at | Ccnd1 | -3 |
| 1423423_at | Pdia3 | -3 |
| 1427033_at | Dnmbp | -3 |
| 1429367_at | Wipi2 | -3 |
| 1430286_s_at | Gm14057 /// Ppp1r14c | -3 |
| 1434564_at | E2f3 | -3 |
| 1455333_at | Tns3 | -3 |
| 1460203_at | Itpr1 | -3 |
| 1416578_at | Rbx1 | -3 |
| 1420880_a_at | Ywhab | -3 |
| 1423102_a_at | Rnf10 | -3 |
| 1423694_at | Kctd10 | -3 |
| 1428423_at | Pcgf3 | -3 |
| 1454670_at | Rere | -3 |
| 1460716_a_at | Cbfb | -3 |
| 1416408_at | Acox1 | -3 |
| 1419246_s_at | Rab14 | -3 |
| 1422713_a_at | Ube2i | -3 |
| 1425978_at | Myocd | -3 |
| 1426015_s_at | Asph | -3 |
| 1428091_at | Klhl7 | -3 |
| 1428325_at | Cnpy4 | -3 |
| 1436118_at | Vangl2 | -3 |
| 1436315_at | Myst3 | -3 |
| 1436703_x_at | Snapc2 | -3 |
| 1447812_x_at | Flnc | -3 |
| 1452217_at | Ahnak | -3 |
| 1452830_s_at | Cad | -3 |
| 1416105_at | Nnt | -3 |
| 1421201_a_at | Tro | -3 |
| 1426368_at | LOC100044115 /// Rin2 | -3 |
| 1421918_at | Anp32a | -3 |
| 1422453_at | Prpf8 | -3 |
| 1423085_at | Efnb3 | -3 |
| 1441947_x_at | Sik3 | -3 |
| 1451736_a_at | Map2k7 | -3 |
| 1453269_at | Unc5b | -3 |
| 1416289_at | Plod1 | -3 |
| 1455389_s_at | 2310051F07Rik | -3 |
| 1442800_x_at | Fam181b | -3 |
| 1426571_at | Ano1 | -3 |
| 1430769_s_at | Fam122a | -3 |
| 1454900_s_at | Mycbp2 | -3 |
| 1428065_at | Slc44a2 | -3 |
| 1432827_x_at | Ubc | -3 |
| 1434075_at | BC030336 | -3 |
| 1434539_at | Lrrn3 | -3 |
| 1434891_at | Ptgfrn | -3 |
| 1452152_at | Clint1 | -3 |
| 1455286_at | Btbd1 | -3 |
| 1422498_at | Mageh1 | -3 |
| 1435417_at | AI464131 | -3 |
| 1416722_at | Hmg20a | -3 |
| 1426790_at | Ssrp1 | -3 |
| 1450852_s_at | F2r | -3 |
| 1451755_a_at | Apobec1 | -3 |
| 1457035_at | AI607873 | -3 |
| 1417311_at | Crip2 | -3 |
| 1424038_a_at | 2310044H10Rik | -3 |
| 1451355_at | Acer2 | -3 |
| 1454753_at | Rnpepl1 | -3 |
| 1416344_at | Lamp2 | -3 |
| 1434011_a_at | Ints5 | -3 |
| 1451469_at | Cntln | -3 |
| 1426947_x_at | Col6a2 | -3 |
| 1447567_at | Odz3 | -3 |
| 1460287_at | Timp2 | -3 |
| 1433643_at | Cacna2d1 | -3 |
| 1460220_a_at | Csf1 | -3 |
| 1429506_at | LOC634379 /// Nkd1 | -3 |
| 1417542_at | Rps6ka2 | -3 |
| 1427785_x_at | Solh | -3 |
| 1433939_at | Aff3 | -3 |
| 1452788_at | Ppp2r5e | -3 |
| 1456084_x_at | Fmod | -3 |
| 1421292_a_at | Cobra1 | -3 |
| 1455492_at | B330016D10Rik | -3 |
| 1435068_at | Pip4k2b | -3 |
| 1421346_a_at | Slc6a6 | -3 |
| 1448502_at | Slc16a7 | -3 |
| 1418892_at | Rhoj | -3 |
| 1454822_x_at | Apcdd1 | -3 |
| 1429064_at | Dip2c | -3 |
| 1455262_at | Thsd4 | -3 |
| 1426801_at | 08-Sep | -3 |
| 1455398_at | Lrrc8c | -3 |
| 1428917_at | Stx17 | -3 |
| 1425270_at | Kif1b | -3 |
| 1440052_at | --- | -3 |
| 1418749_at | Psd3 | -3 |
| 1426218_at | Glcci1 | -3 |
| 1427891_at | Gimap6 | -3 |
| 1430252_at | 3110027N22Rik | -3 |
| 1437886_at | Klhl6 | -3 |
| 1436175_at | Atxn7 | -3 |
| 1451406_a_at | Pcsk5 | -3 |
| 1441590_at | Kcnj5 | -3 |
| 1430379_at | 5830411K21Rik | -3 |
| 1443260_at | Meis1 | -3 |
| 1415686_at | Rab14 | -3 |
| 1434664_at | 2410129H14Rik | -3 |
| 1434555_at | Anp32a | -3 |
| 1415779_s_at | Actg1 | -3 |
| 1417715_a_at | Got2 | -3 |
| 1439436_x_at | Incenp | -3 |
| 1450431_a_at | Nedd4 | -3 |
| 1416488_at | Ccng2 | -3 |
| 1430538_at | 2210013O21Rik | -3 |
| 1433627_at | Sec23ip | -3 |
| 1433820_a_at | 1110012D08Rik | -3 |
| 1435531_at | Usp3 | -3 |
| 1451521_x_at | Eif4h | -3 |
| 1448255_a_at | Surf4 | -3 |
| 1453071_s_at | Kdelc2 | -3 |
| 1456042_s_at | Cramp1l | -3 |
| 1418628_at | Khdrbs1 | -3 |
| 1426342_at | Stt3b | -3 |
| 1426915_at | Dapk1 | -3 |
| 1431012_a_at | Peci | -3 |
| 1448410_at | Ube4b | -3 |
| 1449192_at | Atf7ip | -3 |
| 1452736_at | R3hcc1 | -3 |
| 1419140_at | Acvr2b | -3 |
| 1448402_at | Tln1 | -3 |
| 1434025_at | --- | -3 |
| 1416347_at | Men1 | -3 |
| 1416746_at | H2afx | -3 |
| 1417627_a_at | Limk1 | -3 |
| 1427250_at | Atp2a2 | -3 |
| 1429273_at | Bmper | -3 |
| 1433442_at | Klhl9 | -3 |
| 1434928_at | Gas2l1 | -3 |
| 1448334_a_at | Ccni | -3 |
| 1455089_at | Gng12 | -3 |
| 1455562_at | Sox12 | -3 |
| 1424615_at | Pgap2 | -3 |
| 1429556_at | Tead1 | -3 |
| 1437061_at | Mbd1 | -3 |
| 1438637_x_at | Sf3b2 | -3 |
| 1448189_a_at | Flii | -3 |
| 1423965_at | Cd99l2 | -3 |
| 1435233_at | Ncoa2 | -3 |
| 1437378_x_at | Scarb1 | -3 |
| 1419743_s_at | Carm1 | -3 |
| 1454928_at | Safb | -3 |
| 1455182_at | Kif1b | -3 |
| 1429192_at | Ski | -3 |
| 1434829_at | Cbl | -3 |
| 1447766_x_at | Limd2 | -3 |
| 1450675_at | Smap2 | -3 |
| 1434361_at | Snx33 | -3 |
| 1417846_at | Ulk2 | -3 |
| 1425315_at | Dock7 | -3 |
| 1426376_at | Reep5 | -3 |
| 1435880_at | Ankrd50 | -3 |
| 1435951_at | Grip1 | -3 |
| 1426461_at | Ugp2 | -3 |
| 1428673_at | Sympk | -3 |
| 1448942_at | Gng11 | -3 |
| 1448250_at | 9030425E11Rik | -3 |
| 1436918_at | LOC100044376 | -3 |
| 1438682_at | Pik3r1 | -3 |
| 1456310_a_at | 2610002J02Rik | -3 |
| 1451078_at | 2510039O18Rik | -3 |
| 1418517_at | Irx3 | -3 |
| 1433695_at | Cnrip1 | -3 |
| 1434123_at | Fut11 | -3 |
| 1438442_at | Sike1 | -3 |
| 1453238_s_at | 3930401B19Rik /// A130040M12Rik /// E430024C06Rik | -3 |
| 1450058_at | Asph | -3 |
| 1423746_at | Txndc5 | -3 |
| 1430543_at | Clip3 | -3 |
| 1456070_at | Ptprg | -3 |
| 1424949_at | Huwe1 | -3 |
| 1431033_x_at | Agl | -3 |
| 1437171_x_at | Gsn | -3 |
| 1428285_at | 8430427H17Rik | -3 |
| 1436892_at | Spred2 | -3 |
| 1426973_at | Gpr153 | -3 |
| 1426462_at | Gphn | -3 |
| 1422562_at | Rrad | -3 |
| 1425481_at | Cnot6l | -3 |
| 1439181_at | Zfp658 | -3 |
| 1437506_at | Adamts6 | -3 |
| 1436719_at | Slc35f1 | -3 |
| 1421871_at | Sh3bgrl | -3 |
| 1434401_at | Zcchc2 | -3 |
| 1460718_s_at | Mtch1 | -3 |
| 1421810_at | Dgcr2 | -3 |
| 1444523_s_at | Ube2v1 | -3 |
| 1452285_a_at | Eif3f | -3 |
| 1453367_a_at | Abhd12 | -3 |
| 1455785_at | Kcna1 | -3 |
| 1425052_at | --- | -3 |
| 1436997_x_at | Sh3bgrl | -3 |
| 1417081_a_at | Syngr2 | -3 |
| 1428333_at | 2900062L11Rik | -3 |
| 1428500_at | 2210419D22Rik /// Lrp6 | -3 |
| 1428623_at | Plxna1 | -3 |
| 1434931_at | Neo1 | -3 |
| 1437849_x_at | Armcx2 | -3 |
| 1438554_x_at | Eif4h | -3 |
| 1455468_at | Dpy19l3 | -3 |
| 1415755_a_at | Ube2v1 | -3 |
| 1452733_at | Pank2 | -3 |
| 1418204_s_at | Aif1 | -3 |
| 1424919_at | Erbb2 | -3 |
| 1426677_at | Flna | -3 |
| 1428512_at | Bhlhb9 | -3 |
| 1450870_at | Rala | -3 |
| 1417500_a_at | Tgm2 | -3 |
| 1418758_a_at | Cyth3 | -3 |
| 1422486_a_at | Smad4 | -3 |
| 1437210_a_at | Brd2 | -3 |
| 1450662_at | Tesk1 | -3 |
| 1426831_at | Ahcyl1 | -3 |
| 1428758_at | Tmem86a | -3 |
| 1439382_x_at | Ddr1 | -3 |
| 1440153_at | --- | -3 |
| 1424463_at | Mfsd6 | -3 |
| 1431110_at | Plxdc2 | -3 |
| 1422818_at | Nedd9 | -3 |
| 1423140_at | Lipa | -3 |
| 1423374_at | Ncoa6 | -3 |
| 1449319_at | Rspo1 | -3 |
| 1459840_s_at | Ccdc28b | -3 |
| 1428773_s_at | Bcor | -3 |
| 1429310_at | Flrt3 | -3 |
| 1428469_a_at | Dzip1 | -3 |
| 1429237_at | Stx16 | -3 |
| 1433895_at | Tmem127 | -3 |
| 1436178_at | Leprel1 | -3 |
| 1453200_at | Rai1 | -3 |
| 1455164_at | Arhgap31 | -3 |
| 1436589_x_at | Prkd2 | -3 |
| 1449126_at | Zfp90 | -3 |
| 1425280_at | Leng1 | -3 |
| 1455820_x_at | Scarb1 | -3 |
| 1427894_at | Vasn | -3 |
| 1441656_at | B930068K11Rik | -3 |
| 1454699_at | LOC100047324 /// Sesn1 | -3 |
| 1433651_at | Wtip | -3 |
| 1415977_at | Isyna1 | -3 |
| 1431056_a_at | Lpl | -3 |
| 1457424_at | Eya1 | -3 |
| 1452446_a_at | Tmub2 | -3 |
| 1437419_at | Bmp2k | -3 |
| 1448667_x_at | Tob2 | -3 |
| 1454857_at | Rnf122 | -3 |
| 1426848_at | LOC100047481 /// Sec24b | -3 |
| 1437351_at | Cxxc4 | -3 |
| 1419536_a_at | Rela | -3 |
| 1434326_x_at | Coro2b | -3 |
| 1436913_at | Cdc14a | -3 |
| 1419873_s_at | Csf1r | -3 |
| 1428862_at | Ttc17 | -3 |
| 1441972_at | 6230424C14Rik | -3 |
| 1441746_at | --- | -3 |
| 1436991_x_at | Gsn | -3 |
| 1436979_x_at | Rbm14 | -3 |
| 1431593_a_at | Tsen34 | -3 |
| 1434126_at | 4930402H24Rik | -3 |
| 1434232_a_at | 2610030H06Rik | -3 |
| 1428128_at | 4921506J03Rik | -3 |
| 1459832_s_at | Ap1m1 | -3 |
| 1427535_s_at | Obsl1 | -3 |
| 1418926_at | Zeb1 | -3 |
| 1448645_at | Msl3 | -3 |
| 1449240_at | Gsbs | -3 |
| 1452147_at | Sec24c | -3 |
| 1454931_at | Eid2 | -3 |
| 1436804_s_at | Scyl1 | -3 |
| 1438633_x_at | Lasp1 | -3 |
| 1416509_at | Tm9sf3 | -3 |
| 1433954_at | 4632419I22Rik | -3 |
| 1436783_x_at | Ywhab | -3 |
| 1452055_at | Ctdsp1 | -3 |
| 1452186_at | Rbm5 | -3 |
| 1415818_at | Anxa6 | -3 |
| 1421116_a_at | Rtn4 | -3 |
| 1428540_at | Fam115a | -3 |
| 1435207_at | Dixdc1 | -3 |
| 1450024_at | Sufu | -3 |
| 1455066_s_at | Mia3 | -3 |
| 1456405_at | Dido1 | -3 |
| 1421841_at | Fgfr3 | -3 |
| 1423161_s_at | Spred1 | -3 |
| 1426484_at | Ubxn4 | -3 |
| 1439882_at | Sec23ip | -3 |
| 1449270_at | Plxdc2 | -3 |
| 1426345_at | Prepl | -3 |
| 1419978_s_at | D10Ertd610e | -3 |
| 1427310_at | Bptf | -3 |
| 1428413_at | Ccny /// LOC100044842 | -3 |
| 1436085_at | Zbtb34 | -3 |
| 1436184_at | Znf512b | -3 |
| 1444956_at | --- | -3 |
| 1447693_s_at | Neo1 | -3 |
| 1448435_at | Med15 | -3 |
| 1451071_a_at | Atp1a1 | -3 |
| 1424925_at | Sec63 | -3 |
| 1427081_at | A630072M18Rik | -3 |
| 1428881_at | Klc1 /// LOC100047609 | -3 |
| 1418670_s_at | Hspg2 | -3 |
| 1423899_at | Trip12 | -3 |
| 1434880_at | Etv6 | -3 |
| 1438407_at | Dsel | -3 |
| 1460647_a_at | Nr2f6 | -3 |
| 1421813_a_at | Psap | -3 |
| 1433481_at | Fkbp14 | -3 |
| 1444232_at | Prkg1 | -3 |
| 1452690_at | Khsrp | -3 |
| 1417295_at | Mta1 | -3 |
| 1421604_a_at | Klf3 /// LOC100046855 | -3 |
| 1452878_at | Prkce | -3 |
| 1435916_at | Zfp84 | -3 |
| 1422733_at | Fjx1 | -3 |
| 1439077_at | Zxda | -3 |
| 1434383_at | Pja2 | -3 |
| 1423690_s_at | Gpsm1 | -3 |
| 1426260_a_at | Ugt1a1 /// Ugt1a10 /// Ugt1a2 /// Ugt1a5 /// Ugt1a6a /// Ugt1a6b /// Ugt1a7c /// Ugt1a9 | -3 |
| 1447943_x_at | Yeats2 | -3 |
| 1460586_at | Megf8 | -3 |
| 1435903_at | Cd300a | -3 |
| 1436590_at | Ppp1r3b | -3 |
| 1424208_at | Ptger4 | -3 |
| 1441693_at | Adamts3 | -3 |
| 1433717_at | D19Wsu162e | -3 |
| 1454869_at | Dcaf12l1 | -3 |
| 1457229_at | Gpr173 | -3 |
| 1437150_at | Fam110b | -3 |
| 1441022_at | Arih1 | -3 |
| 1435774_at | LOC106740 | -3 |
| 1435574_at | --- | -3 |
| 1449459_s_at | Asb13 | -3 |
| 1416619_at | 4632428N05Rik | -3 |
| 1426349_s_at | Tmpo | -3 |
| 1428127_at | 4921506J03Rik | -3 |
| 1447447_s_at | Srrm1 | -3 |
| 1455042_at | Tbl1x | -3 |
| 1427005_at | Plk2 | -3 |
| 1416483_at | Ttc3 | -3 |
| 1434331_at | Eif2c1 | -3 |
| 1426648_at | Mapkapk2 | -3 |
| 1424373_at | Armcx3 | -3 |
| 1415689_s_at | Zkscan3 | -3 |
| 1454899_at | Lpp | -3 |
| 1455929_x_at | Ppp2r1a | -3 |
| 1459874_s_at | Mtmr4 | -3 |
| 1449333_at | Sf3a1 | -3 |
| 1417128_at | Plekho1 | -3 |
| 1428822_a_at | Snx24 | -3 |
| 1428902_at | Chst11 | -3 |
| 1430561_at | Dnajb14 | -3 |
| 1435272_at | Itpkb | -3 |
| 1416630_at | Id3 | -3 |
| 1423795_at | Sfpq | -3 |
| 1427489_at | Itga8 | -3 |
| 1428269_a_at | Glt8d1 | -3 |
| 1429718_at | Slitrk5 | -3 |
| 1433500_at | Dennd2a | -3 |
| 1434806_at | Mtx3 | -3 |
| 1452769_at | Rnf145 | -3 |
| 1426972_at | Sec24d | -3 |
| 1448779_at | Ciz1 | -3 |
| 1451344_at | Tmem119 | -3 |
| 1455651_at | Terf2 | -3 |
| 1417607_at | Cox6a2 | -3 |
| 1423796_at | Sfpq | -3 |
| 1424797_a_at | Pitx2 | -3 |
| 1426764_at | Oaz2 | -3 |
| 1433890_a_at | Bat3 | -3 |
| 1434106_at | Epm2aip1 | -3 |
| 1435149_at | Plcg1 | -3 |
| 1436319_at | Sulf1 | -3 |
| 1437377_a_at | Polrmt | -3 |
| 1437385_at | Ccbe1 | -3 |
| 1438029_at | Rprd2 | -3 |
| 1444058_at | Dzip3 | -3 |
| 1448400_a_at | Smarcd2 | -3 |
| 1452225_at | 2010106G01Rik | -3 |
| 1416796_at | LOC100044475 /// Nck2 | -3 |
| 1438370_x_at | Dos | -3 |
| 1450872_s_at | Lipa | -3 |
| 1452915_at | Prkar2a | -3 |
| 1416324_s_at | Kctd20 | -3 |
| 1421147_at | Terf2 | -3 |
| 1423321_at | Myadm | -3 |
| 1423831_at | Prkag2 | -3 |
| 1428657_at | Rreb1 | -3 |
| 1439389_s_at | Myadm | -3 |
| 1422662_at | Lgals8 | -3 |
| 1428174_x_at | Khsrp | -3 |
| 1434964_at | Amz2 | -3 |
| 1452283_at | Rassf8 | -3 |
| 1416666_at | Serpine2 | -3 |
| 1427483_at | Slc25a24 | -3 |
| 1433581_at | 1190002N15Rik /// LOC100044725 | -3 |
| 1434487_at | Mef2d | -3 |
| 1439433_a_at | Slc35a2 | -3 |
| 1439495_at | 4933407H18Rik | -3 |
| 1439833_at | 03-Sep | -3 |
| 1455310_at | Rbm16 | -3 |
| 1416568_a_at | Acin1 | -3 |
| 1422631_at | Ahr | -3 |
| 1460003_at | AI956758 | -3 |
| 1449885_at | Tmem47 | -3 |
| 1418569_at | Fblim1 | -3 |
| 1426465_at | Dlgap4 | -3 |
| 1426934_at | Nhsl1 | -3 |
| 1453078_at | 2610002M06Rik | -3 |
| 1433657_at | Fam78a | -3 |
| 1425452_s_at | Fam84a | -3 |
| 1447877_x_at | Dnmt1 | -3 |
| 1448875_at | Zhx1 | -3 |
| 1426282_at | Ntm | -3 |
| 1424754_at | Ms4a7 | -3 |
| 1426569_a_at | Frk | -3 |
| 1451174_at | Lrrc33 | -3 |
| 1452913_at | Pcp4l1 | -3 |
| 1438931_s_at | LOC100047324 /// Sesn1 | -3 |
| 1452922_at | Ppp1r3d | -3 |
| 1454625_at | Phf6 | -3 |
| 1436879_x_at | Afp | -3 |
| 1452632_at | Aak1 | -3 |
| 1428444_at | Asb2 | -3 |
| 1424076_at | Gdpd1 | -3 |
| 1435415_x_at | Marcksl1 | -3 |
| 1437289_at | Impad1 | -3 |
| 1450649_at | Gng10 | -3 |
| 1417090_at | Rcn1 | -3 |
| 1455875_x_at | Tm9sf2 | -3 |
| 1417039_a_at | Cul7 | -3 |
| 1428107_at | Sh3bgrl | -3 |
| 1426754_x_at | Ckap4 | -3 |
| 1435612_at | Opcml | -3 |
| 1435136_at | Whsc1 | -3 |
| 1416021_a_at | Fabp5 /// Gm6166 | -3 |
| 1422791_at | Pafah1b2 | -3 |
| 1423896_a_at | Rnf187 | -3 |
| 1425966_x_at | Ubc | -3 |
| 1451575_a_at | LOC100047647 /// Nudt3 | -3 |
| 1423702_at | H1f0 | -3 |
| 1424005_at | B230219D22Rik | -3 |
| 1433719_at | Slc9a9 | -3 |
| 1434957_at | Cdon | -3 |
| 1456005_a_at | Bcl2l11 | -3 |
| 1426245_s_at | Mapre2 | -3 |
| 1448392_at | Sparc | -3 |
| 1450078_at | Nrk | -3 |
| 1417165_at | Mbd2 | -3 |
| 1422611_s_at | Igf2bp3 | -3 |
| 1429764_at | Fam101b | -3 |
| 1436018_at | Mex3a | -3 |
| 1437521_s_at | Ammecr1l | -3 |
| 1444089_at | Spnb2 | -3 |
| 1455246_at | Smarcc1 | -3 |
| 1437389_x_at | Khdrbs1 | -3 |
| 1451091_at | Txndc5 | -3 |
| 1416836_at | Lrp10 | -3 |
| 1416481_s_at | Higd1a | -3 |
| 1416724_x_at | Tcf4 | -3 |
| 1424198_at | Dlg5 | -3 |
| 1435437_at | Setd7 | -3 |
| 1448526_at | Kpnb1 | -3 |
| 1449576_at | Eif1ax | -3 |
| 1451188_at | Wdr26 | -3 |
| 1455712_at | Hist3h2a | -3 |
| 1418887_a_at | D11Wsu99e | -3 |
| 1420851_at | Pard6g | -3 |
| 1423407_a_at | Fbln2 | -3 |
| 1424410_at | Ttc8 | -3 |
| 1444693_at | --- | -3 |
| 1422541_at | Ptprm | -3 |
| 1425050_at | Isoc1 | -3 |
| 1428412_at | Tm9sf3 | -3 |
| 1428463_a_at | Ppp2r5e | -3 |
| 1429438_at | Bcor | -3 |
| 1434937_at | Mycbp2 | -3 |
| 1435448_at | Bcl2l11 | -3 |
| 1452760_at | 2510012J08Rik | -3 |
| 1428637_at | Dyrk2 /// LOC100044376 | -3 |
| 1438664_at | Prkar2b | -3 |
| 1455031_at | Cdk19 | -3 |
| 1416589_at | Sparc | -3 |
| 1427410_at | Dleu2 | -3 |
| 1418402_at | Adam19 /// LOC100045780 | -3 |
| 1418876_at | Foxd1 | -3 |
| 1433605_at | Inpp5a | -3 |
| 1420443_at | Pcdhb19 | -3 |
| 1427560_at | Six5 | -3 |
| 1428618_at | Hcfc2 | -3 |
| 1442352_at | 9430091N11Rik | -3 |
| 1454941_at | LOC100045684 /// Nmt1 | -3 |
| 1423718_at | Ak3 | -3 |
| 1454630_at | Samd14 | -3 |
| 1427934_at | Lyrm2 | -3 |
| 1419047_at | Pcnx | -3 |
| 1419243_at | Rab14 | -3 |
| 1419401_at | Asb13 | -3 |
| 1435043_at | Plcb1 | -3 |
| 1460342_s_at | Mprip | -3 |
| 1422245_a_at | Mrvi1 | -3 |
| 1459301_at | --- | -3 |
| 1448251_at | 9030425E11Rik | -3 |
| 1416818_at | Parva | -3 |
| 1447100_s_at | 5730508B09Rik | -3 |
| 1451104_a_at | Snrnp70 | -3 |
| 1428158_at | Akt1s1 | -3 |
| 1448892_at | Dock7 | -3 |
| 1449261_at | Pbx2 | -3 |
| 1450699_at | Selenbp1 | -3 |
| 1415983_at | Lcp1 | -3 |
| 1427177_at | Fyco1 | -3 |
| 1423928_at | Ubac2 | -3 |
| 1440201_at | Slc8a1 | -3 |
| 1454086_a_at | Lmo2 | -3 |
| 1457040_at | Lgi2 | -3 |
| 1424603_at | Sumf1 | -3 |
| 1436956_at | --- | -3 |
| 1434834_at | Socs7 | -3 |
| 1420909_at | Vegfa | -3 |
| 1440836_at | Setd1b | -3 |
| 1417519_at | Plagl2 | -3 |
| 1433455_at | LOC100047863 | -3 |
| 1455592_at | Vangl2 | -3 |
| 1418801_at | Zkscan1 | -3 |
| 1443485_at | Epha7 | -3 |
| 1447903_x_at | Ap1s2 | -3 |
| 1428891_at | Parm1 | -3 |
| 1418746_at | Pnkd | -3 |
| 1451003_at | Tab2 | -3 |
| 1451679_at | 6530401D17Rik | -3 |
| 1418255_s_at | Srf | -3 |
| 1452330_a_at | Mxra8 | -3 |
| 1428339_at | Nudt21 | -3 |
| 1438383_x_at | Ppp2r1a | -3 |
| 1438991_x_at | Ppp2r1a | -3 |
| 1424480_s_at | Akt2 | -3 |
| 1455955_s_at | Snx17 | -3 |
| 1417440_at | Arid1a | -3 |
| 1450877_at | BC005537 | -3 |
| 1454740_at | Mib1 | -3 |
| 1455594_at | Exoc3 | -3 |
| 1420879_a_at | Ywhab | -3 |
| 1426012_a_at | 2610301G19Rik | -3 |
| 1433979_at | Rbms2 | -3 |
| 1416949_s_at | Slc39a7 | -3 |
| 1428373_at | Ip6k2 | -3 |
| 1433568_at | Papd4 | -3 |
| 1450449_a_at | Rilpl1 | -3 |
| 1454920_at | Uhrf2 | -3 |
| 1428349_s_at | Ebf3 | -3 |
| 1454696_at | Gnb1 | -3 |
| 1455746_at | Kif13a | -3 |
| 1419024_at | Ptp4a1 | -3 |
| 1425115_at | Rbbp6 | -3 |
| 1436944_x_at | Pisd-ps1 /// Pisd-ps3 | -3 |
| 1437154_at | Cep170 | -3 |
| 1451020_at | Gsk3b | -3 |
| 1419244_a_at | Rab14 | -3 |
| 1422490_at | Bnip2 | -3 |
| 1425745_a_at | Tacc2 | -3 |
| 1427296_at | Fam120a | -3 |
| 1433851_at | Ppp4r2 | -3 |
| 1450072_at | Ash1l | -3 |
| 1452669_at | Fam53c | -3 |
| 1448155_at | Pdcd6ip | -3 |
| 1421321_a_at | Net1 | -3 |
| 1443018_at | --- | -3 |
| 1460554_s_at | Glg1 | -3 |
| 1419835_s_at | Plec | -3 |
| 1421908_a_at | Tcf12 | -3 |
| 1437149_at | Slc6a6 | -3 |
| 1448926_at | Hoxa5 | -3 |
| 1422208_a_at | Gnb5 | -3 |
| 1421066_at | Jak2 | -3 |
| 1450687_at | Igf2bp3 | -3 |
| 1455303_at | Rfxap | -3 |
| 1427056_at | Adamts15 | -3 |
| 1435433_at | Agap1 | -3 |
| 1450637_a_at | Aebp1 | -3 |
| 1429599_a_at | Mthfd2l | -3 |
| 1418983_at | Inadl | -3 |
| 1424666_at | Gpatch8 | -3 |
| 1436190_at | Zfp618 | -3 |
| 1428383_a_at | 2310021P13Rik | -3 |
| 1436002_at | Scube3 | -3 |
| 1444158_at | Kdm5c | -3 |
| 1455009_at | Cpd | -3 |
| 1448749_at | Plek | -3 |
| 1428603_at | Gm16039 | -3 |
| 1435990_at | Adamts2 | -3 |
| 1419519_at | Igf1 | -3 |
| 1442129_at | --- | -3 |
| 1439817_at | 2900064A13Rik | -3 |
| 1435492_at | Socs6 | -3 |
| 1425383_a_at | LOC676870 /// Pbx1 | -3 |
| 1428432_at | Zcchc24 | -3 |
| 1455548_at | Dlgap4 | -3 |
| 1439015_at | Gfra1 | -3 |
| 1443773_at | Ylpm1 | -3 |
| 1420753_at | Tll1 | -3 |
| 1427042_at | Mal2 | -3 |
| 1440650_at | --- | -3 |
| 1416621_at | Llgl1 | -3 |
| 1424128_x_at | Aurkb | -3 |
| 1415908_at | Tspyl1 | -3 |
| 1448548_at | Tulp4 | -3 |
| 1443394_at | --- | -3 |
| 1451984_at | Hnrnpul1 | -3 |
| 1416498_at | Ppic | -3 |
| 1422610_s_at | Igf2bp3 | -3 |
| 1426207_at | Ikbkb | -3 |
| 1433925_at | Dync1li2 | -3 |
| 1436226_at | Tceb1 | -3 |
| 1416941_s_at | Eif4h | -3 |
| 1418258_s_at | Dynll2 | -3 |
| 1419157_at | Sox4 | -3 |
| 1423069_at | Adnp | -3 |
| 1433833_at | Fndc3b | -3 |
| 1426956_a_at | Trp53bp1 | -3 |
| 1455815_a_at | Ywhab | -3 |
| 1423679_at | 2810432L12Rik | -3 |
| 1428230_at | Prkd3 | -3 |
| 1431390_a_at | Grinl1a | -3 |
| 1455529_at | Mex3a | -3 |
| 1416612_at | Cyp1b1 | -3 |
| 1460246_at | Mecp2 | -3 |
| 1451728_at | Wdr13 | -3 |
| 1418260_at | Hunk | -3 |
| 1426992_at | Xpr1 | -3 |
| 1427162_a_at | Elk4 | -3 |
| 1429084_at | Vezf1 | -3 |
| 1431028_a_at | Pank1 | -3 |
| 1447894_x_at | H2afb3 | -3 |
| 1449117_at | Jund | -3 |
| 1451021_a_at | Klf5 | -3 |
| 1455396_at | Atp8b1 | -3 |
| 1456566_x_at | Rbm14 | -3 |
| 1460116_s_at | Spred1 | -3 |
| 1456573_x_at | Nnt | -3 |
| 1456786_at | Ldb2 | -3 |
| 1448434_at | Rnf103 | -3 |
| 1417866_at | Tnfaip1 | -3 |
| 1427038_at | Penk | -3 |
| 1433983_at | Cnksr3 | -3 |
| 1453197_at | Med25 | -3 |
| 1455059_at | Helz | -3 |
| 1443881_at | Pofut1 | -3 |
| 1415904_at | Lpl | -3 |
| 1422168_a_at | Bdnf | -3 |
| 1427985_at | Spin4 | -3 |
| 1435414_s_at | Dctn1 | -3 |
| 1417204_at | Kdelr2 | -3 |
| 1430425_at | Sdk2 | -3 |
| 1455928_x_at | Lztr1 | -3 |
| 1433558_at | Dab2ip | -3 |
| 1434655_at | Foxk1 | -3 |
| 1438634_x_at | Lasp1 | -3 |
| 1417558_at | Fyn | -3 |
| 1420484_a_at | Vtn | -3 |
| 1422754_at | Tmod1 | -3 |
| 1426348_at | Col4a1 | -3 |
| 1437810_a_at | Hbb-bh1 | -3 |
| 1429468_at | 1110018F16Rik /// Dnajb14 | -3 |
| 1434133_s_at | Dcaf8 | -3 |
| 1429678_at | 5730508B09Rik | -3 |
| 1437633_at | Ankrd11 | -3 |
| 1438038_at | 4930402H24Rik | -3 |
| 1422540_at | Fbln1 | -3 |
| 1433825_at | Ntrk3 | -3 |
| 1424239_at | Fam65a | -3 |
| 1419549_at | Arg1 | -3 |
| 1417839_at | Cldn5 | -3 |
| 1456392_at | Negr1 | -3 |
| 1416002_x_at | Cotl1 | -3 |
| 1429362_a_at | Sf3b2 | -3 |
| 1420422_at | Pcdhb21 | -3 |
| 1435292_at | Tbc1d4 | -3 |
| 1437360_at | Pcdh19 | -3 |
| 1437675_at | Slc8a1 | -3 |
| 1424932_at | Egfr | -3 |
| 1416444_at | Elovl2 | -3 |
| 1451986_s_at | Lrrk1 | -3 |
| 1458229_at | Robo2 | -3 |
| 1457635_s_at | Nr3c1 | -3 |
| 1441547_at | --- | -3 |
| 1422946_a_at | Dnmt1 | -3 |
| 1428249_at | Pygo2 | -3 |
| 1437103_at | Igf2bp2 | -3 |
| 1454745_at | Arhgap29 | -3 |
| 1415765_at | Hnrnpul2 | -3 |
| 1424275_s_at | Trim41 | -3 |
| 1434338_at | Rgp1 | -3 |
| 1435591_at | AI426330 | -3 |
| 1448363_at | Yap1 | -3 |
| 1450667_a_at | Cs | -3 |
| 1451286_s_at | Fus | -3 |
| 1422578_at | Cs | -3 |
| 1437325_x_at | Aldh18a1 | -3 |
| 1437382_at | Acvr2a | -3 |
| 1415707_at | Anapc2 | -3 |
| 1423532_at | Rnf44 | -3 |
| 1425985_s_at | Masp1 | -3 |
| 1433997_at | Klhdc10 | -3 |
| 1436885_a_at | Cherp | -3 |
| 1437967_at | Ccdc141 | -3 |
| 1455547_at | Zc3h7b | -3 |
| 1455794_at | Smtnl2 | -3 |
| 1460559_at | Kank2 | -3 |
| 1416511_a_at | Cdc42ep4 | -3 |
| 1450686_at | Pon2 | -3 |
| 1418492_at | Grem2 | -3 |
| 1428342_at | Rcor3 | -3 |
| 1426863_at | Rbmx | -3 |
| 1436962_at | Prdm6 | -3 |
| 1452309_at | Cgnl1 | -3 |
| 1455155_at | Lsm14b | -3 |
| 1456253_s_at | Klhl17 | -3 |
| 1424780_a_at | Reep3 | -3 |
| 1438684_at | Nuak1 | -3 |
| 1422731_at | Limd1 | -3 |
| 1433632_at | Irf2bp2 | -3 |
| 1449141_at | Fblim1 | -3 |
| 1437132_x_at | Nedd9 | -3 |
| 1456482_at | Pik3r3 | -3 |
| 1417663_a_at | Ndrg3 | -3 |
| 1419905_s_at | Hpgd | -3 |
| 1451177_at | Dnajb4 | -3 |
| 1452331_s_at | Qser1 | -3 |
| 1418776_at | Gbp8 | -3 |
| 1429413_at | Cpm | -3 |
| 1429861_at | Pcdh9 | -3 |
| 1416732_at | Top2b | -3 |
| 1428207_at | Bcl7a | -3 |
| 1435648_at | Lrrn4 | -3 |
| 1456974_at | Onecut1 | -3 |
| 1460121_at | 9630010G10Rik | -3 |
| 1447040_at | --- | -3 |
| 1455090_at | Angptl2 | -3 |
| 1430407_at | 3110035C09Rik | -3 |
| 1437667_a_at | Bach2 | -3 |
| 1452141_a_at | Sepp1 | -3 |
| 1436234_at | 4732471D19Rik | -3 |
| 1451315_at | Tmem101 | -3 |
| 1451574_at | Bcl9 | -3 |
| 1415986_at | Clcn4-2 | -3 |
| 1418046_at | Nap1l2 | -3 |
| 1435020_at | Klhdc2 | -3 |
| 1455176_a_at | Syt11 | -3 |
| 1415806_at | Plat | -3 |
| 1438402_at | Fam171a1 | -3 |
| 1438072_at | --- | -3 |
| 1456212_x_at | Socs3 | -3 |
| 1435595_at | 1810011O10Rik | -3 |
| 1417701_at | Ppp1r14c | -3 |
| 1434639_at | Klhl29 | -3 |
| 1435540_at | Irgq | -3 |
| 1452751_at | Ebf3 | -3 |
| 1423990_at | Rab28 | -3 |
| 1417112_at | Arl2bp | -3 |
| 1418300_a_at | Mknk2 | -3 |
| 1415981_at | Herpud2 | -3 |
| 1426834_s_at | D930015E06Rik | -3 |
| 1433976_at | Reep3 | -3 |
| 1438200_at | Sulf1 | -3 |
| 1449893_a_at | Lrig1 | -3 |
| 1416708_a_at | Gramd1a | -3 |
| 1425480_at | Cnot6l | -3 |
| 1433668_at | Pnrc1 | -3 |
| 1415799_at | Wbp11 | -3 |
| 1426763_at | Oaz2 | -3 |
| 1433501_at | Ctso | -3 |
| 1435778_at | Ankrd11 | -3 |
| 1417038_at | 09-Sep | -3 |
| 1423462_at | Tab2 | -3 |
| 1447657_s_at | Synpo2l | -3 |
| 1452767_at | Rrbp1 | -3 |
| 1460331_at | Tm9sf2 | -3 |
| 1455524_at | 2810046L04Rik | -3 |
| 1419866_s_at | Atxn2 /// LOC100047323 | -3 |
| 1419938_s_at | Arhgef17 | -3 |
| 1426670_at | Agrn | -3 |
| 1450102_a_at | Amfr /// LOC100046262 | -3 |
| 1450741_at | Stau1 | -3 |
| 1452589_at | Ptk7 | -3 |
| 1449135_at | Sox18 | -3 |
| 1416442_at | Ier2 | -3 |
| 1418373_at | Pgam2 | -3 |
| 1424781_at | Reep3 | -3 |
| 1430133_at | Tbc1d8b | -3 |
| 1433572_a_at | Fam120a | -3 |
| 1434940_x_at | Rgs19 | -3 |
| 1435468_at | B3gnt9 | -3 |
| 1436537_at | Zfp629 | -3 |
| 1454738_x_at | Pex6 | -3 |
| 1454995_at | Ddah1 | -3 |
| 1457633_x_at | Cox6a2 | -3 |
| 1428266_at | Myl3 | -3 |
| 1437921_x_at | Zfp516 | -3 |
| 1438401_at | Ubn1 | -3 |
| 1449852_a_at | Ehd4 | -3 |
| 1455402_at | Socs7 | -3 |
| 1417893_at | Sfxn3 | -3 |
| 1418540_a_at | Ptpre | -3 |
| 1419564_at | Zfp467 | -3 |
| 1434557_at | Hip1 | -3 |
| 1447584_s_at | Myct1 | -3 |
| 1424616_s_at | Pgap2 | -3 |
| 1451295_a_at | Chd4 | -3 |
| 1455205_a_at | Usp19 | -3 |
| 1457246_at | Fam160b2 | -3 |
| 1416895_at | Efna1 | -3 |
| 1423295_at | Tm9sf2 | -3 |
| 1435170_at | Tsr2 | -3 |
| 1436789_at | Ccnjl | -3 |
| 1448666_s_at | Tob2 | -3 |
| 1436827_at | Fam59a | -3 |
| 1428117_x_at | Dynlt1-ps1 /// Dynlt1a /// Dynlt1b /// Dynlt1c /// Dynlt1d /// Dynlt1f | -3 |
| 1451246_s_at | Aurkb | -3 |
| 1455091_at | Ppp2r3a | -3 |
| 1449017_at | Nutf2 | -3 |
| 1437545_at | Rcor1 | -3 |
| 1416022_at | Fabp5 | -3 |
| 1438201_at | Pdp1 | -3 |
| 1455584_at | Sdf4 | -3 |
| 1442724_at | Dlk1 | -3 |
| 1424768_at | Cald1 | -3 |
| 1426251_at | Cpz | -3 |
| 1448862_at | Icam2 | -3 |
| 1448390_a_at | Dhrs3 | -3 |
| 1422890_at | Pcdh18 | -3 |
| 1426063_a_at | Gem | -3 |
| 1453406_a_at | Rab28 | -3 |
| 1418629_a_at | Khdrbs1 | -3 |
| 1434291_a_at | Serf1 | -3 |
| 1451912_a_at | Fgfrl1 /// LOC100046239 | -3 |
| 1415715_at | Tmem129 | -3 |
| 1416683_at | Plxnb2 | -3 |
| 1421861_at | Clstn1 | -3 |
| 1422629_s_at | Shroom3 | -3 |
| 1426682_at | Cnot6 | -3 |
| 1434551_at | Hnrnpul2 | -3 |
| 1448778_at | Sfrs4 | -3 |
| 1455314_at | Lpp | -3 |
| 1455581_x_at | 9530028C05 | -3 |
| 1417848_at | Zfp704 | -3 |
| 1460328_at | Brd3 | -3 |
| 1426373_at | Ski | -3 |
| 1437025_at | Cd28 | -3 |
| 1444761_at | --- | -3 |
| 1448647_at | Man2a1 | -3 |
| 1424281_at | Ubap2 | -3 |
| 1429783_at | Pdlim5 | -3 |
| 1433461_at | Sf3b2 | -3 |
| 1451347_at | Ino80e | -3 |
| 1451980_at | Casd1 | -3 |
| 1455049_at | Igsf3 | -3 |
| 1418703_at | Rbms1 | -3 |
| 1423809_at | Tcf19 | -3 |
| 1434416_a_at | Solh | -3 |
| 1448396_at | Tmem131 | -3 |
| 1418672_at | Akr1c13 | -3 |
| 1429739_a_at | Patz1 | -3 |
| 1434541_x_at | Khdrbs1 | -3 |
| 1455521_at | Klf12 | -3 |
| 1425463_at | Gata6 | -3 |
| 1435326_at | Lpgat1 | -3 |
| 1416427_at | Ccni | -3 |
| 1428126_a_at | 4921506J03Rik | -3 |
| 1435397_at | Zbtb44 | -3 |
| 1448416_at | Mgp | -3 |
| 1450902_at | Brd3 | -3 |
| 1452377_at | Mll1 | -3 |
| 1455668_at | Whsc1l1 | -3 |
| 1416527_at | Rab32 | -3 |
| 1417707_at | N4bp2l1 | -3 |
| 1417084_at | Eif4ebp2 | -3 |
| 1438999_a_at | Nfat5 | -3 |
| 1452346_at | B3gnt1 | -3 |
| 1435985_at | Farp2 | -3 |
| 1425803_a_at | Mbd2 | -3 |
| 1418634_at | Notch1 | -3 |
| 1451063_at | Stxbp4 | -3 |
| 1452882_at | Pgrmc2 | -3 |
| 1426771_at | Tug1 | -3 |
| 1437303_at | Il6st | -3 |
| 1419482_at | C3ar1 | -3 |
| 1430125_s_at | Pqlc1 | -3 |
| 1438004_at | Papd7 | -3 |
| 1441823_at | Zmiz1 | -3 |
| 1443823_s_at | Atp1a2 | -3 |
| 1437404_at | Mast4 | -3 |
| 1419688_at | Gpc6 | -3 |
| 1448161_a_at | Clcn4-2 | -3 |
| 1454959_s_at | Gnai1 | -3 |
| 1424113_at | Lamb1-1 | -3 |
| 1455306_at | Fam193a | -3 |
| 1416590_a_at | Rab34 | -3 |
| 1416418_at | Gabarapl1 | -3 |
| 1417865_at | Tnfaip1 | -3 |
| 1437811_x_at | --- | -3 |
| 1423750_a_at | Sf1 | -3 |
| 1424882_a_at | Nt5dc2 | -3 |
| 1425142_a_at | Hnrnpd | -3 |
| 1437151_at | Usp22 | -3 |
| 1447624_s_at | Stox2 | -3 |
| 1448154_at | Ndrg2 | -3 |
| 1449441_a_at | Wbp1 | -3 |
| 1450723_at | Isl1 | -3 |
| 1448863_a_at | Tnfaip1 | -3 |
| 1416044_at | Flii | -3 |
| 1424094_at | Nek9 | -3 |
| 1427476_a_at | Trim32 | -3 |
| 1433736_at | Hcfc1 | -3 |
| 1442542_at | Eya4 | -3 |
| 1452149_at | Ube3b | -3 |
| 1435449_at | Bcl2l11 | -3 |
| 1451225_at | Ptpn11 | -3 |
| 1428871_at | LOC100047441 /// Msl1 | -3 |
| 1429085_at | Vezf1 | -3 |
| 1460211_a_at | Kdelr1 | -3 |
| 1438171_x_at | Mettl9 | -3 |
| 1444157_a_at | Kdm5c | -3 |
| 1437482_at | Tecrl | -3 |
| 1417287_at | H13 | -3 |
| 1458299_s_at | Nfkbie | -3 |
| 1458370_at | Bmp2k | -3 |
| 1455267_at | Esrrg | -3 |
| 1422139_at | Plau | -3 |
| 1427469_at | Helz | -3 |
| 1439485_at | Zfp608 | -3 |
| 1422710_a_at | Cacna1h | -3 |
| 1429515_at | Ubr2 | -3 |
| 1452903_at | 6230427J02Rik | -3 |
| 1427129_a_at | Hnrnpr | -3 |
| 1417148_at | Pdgfrb | -3 |
| 1416257_at | Capn2 | -3 |
| 1428853_at | Ptch1 | -3 |
| 1433868_at | Btbd3 | -3 |
| 1440310_at | Runx1t1 | -3 |
| 1440911_at | Col23a1 | -3 |
| 1426024_a_at | Dbn1 | -3 |
| 1438143_s_at | Atxn2 /// LOC100047323 | -3 |
| 1418497_at | Fgf13 | -3 |
| 1416903_at | Nucb1 | -3 |
| 1438476_a_at | Chd4 | -3 |
| 1455622_at | Podxl2 | -3 |
| 1455149_at | Sh3rf1 | -3 |
| 1442350_at | --- | -3 |
| 1426574_a_at | Add3 | -3 |
| 1435627_x_at | Marcksl1 | -3 |
| 1434403_at | Spred2 | -3 |
| 1440441_at | Fbxl7 | -3 |
| 1427918_a_at | Rhoq | -3 |
| 1435824_at | Yy1 | -3 |
| 1416130_at | Prnp | -3 |
| 1416591_at | Rab34 | -3 |
| 1429019_s_at | Pon2 | -3 |
| 1434360_s_at | LOC632664 /// Ptprg | -3 |
| 1455741_a_at | Ece1 | -3 |
| 1416701_at | Rnd3 | -3 |
| 1423885_at | Lamc1 | -3 |
| 1428074_at | Tmem158 | -3 |
| 1455653_at | Ccnj | -3 |
| 1416645_a_at | Afp | -3 |
| 1436838_x_at | Cotl1 | -3 |
| 1457666_s_at | Ifi202b | -3 |
| 1424652_at | Fam176a | -3 |
| 1436305_at | Rnf217 | -3 |
| 1427295_at | Slc38a10 | -3 |
| 1435197_at | Pou3f3 | -3 |
| 1449066_a_at | Arhgef7 | -3 |
| 1433546_at | Gns | -3 |
| 1418198_a_at | Tm9sf1 | -3 |
| 1428431_at | Zcchc24 | -3 |
| 1448737_at | Tspan7 | -3 |
| 1416268_at | Ets2 | -3 |
| 1422431_at | Magee1 | -3 |
| 1448939_at | Usp25 | -3 |
| 1456483_at | Zfp9 | -3 |
| 1455135_at | Prr24 | -3 |
| 1427932_s_at | 1200003I10Rik /// 1200015M12Rik /// A130040M12Rik /// E430024C06Rik | -3 |
| 1434384_at | Nrip1 | -3 |
| 1450071_at | Ash1l | -3 |
| 1435743_at | Klhl23 | -3 |
| 1438020_at | Hapln1 | -3 |
| 1440084_at | --- | -3 |
| 1450910_at | Cap2 | -3 |
| 1455717_s_at | Daam2 | -3 |
| 1420895_at | Tgfbr1 | -3 |
| 1415770_at | Wdr6 | -3 |
| 1420992_at | Ankrd1 | -3 |
| 1423038_at | Stx6 | -3 |
| 1434101_at | Nfib | -3 |
| 1450803_at | Ntf3 | -3 |
| 1431362_a_at | Smoc2 | -3 |
| 1440920_at | Mmp14 | -3 |
| 1439901_at | --- | -3 |
| 1436181_at | Asap2 | -3 |
| 1451287_s_at | Aif1l | -3 |
| 1428541_at | Fam115a | -3 |
| 1437594_x_at | Pigt | -3 |
| 1417490_at | Ctsb | -3 |
| 1437829_s_at | Eef2k | -3 |
| 1434260_at | Fchsd2 | -3 |
| 1424189_at | Pigc | -3 |
| 1434530_at | Odz4 | -3 |
| 1440999_at | Zfp697 | -3 |
| 1440253_at | Psmd11 | -3 |
| 1416006_at | Mdk | -3 |
| 1428286_at | 2900097C17Rik | -3 |
| 1455002_at | Ptp4a1 | -3 |
| 1434807_s_at | Mtx3 | -3 |
| 1435866_s_at | Hist3h2a | -3 |
| 1439764_s_at | Igf2bp2 | -3 |
| 1433439_at | Cpne1 | -3 |
| 1456326_at | Fndc3c1 /// LOC676436 | -3 |
| 1435338_at | Cdk6 | -3 |
| 1456768_a_at | Mmrn2 | -3 |
| 1423152_at | Vapb | -3 |
| 1436774_at | Sel1l | -3 |
| 1460205_at | Dcakd | -3 |
| 1455082_at | Cblb | -3 |
| 1458407_s_at | --- | -3 |
| 1434443_at | Anapc1 | -3 |
| 1452249_at | Prickle1 | -3 |
| 1435542_s_at | Cttnbp2nl | -3 |
| 1455169_at | Rab11fip2 | -3 |
| 1426913_at | Lss | -3 |
| 1428808_at | Prickle2 | -3 |
| 1450069_a_at | Celf2 | -3 |
| 1451268_at | Tram1l1 | -3 |
| 1452123_s_at | Frmd4b | -3 |
| 1416200_at | Il33 | -3 |
| 1426690_a_at | Srebf1 | -3 |
| 1428498_at | Rnf219 | -3 |
| 1429428_at | Tcf7l2 | -3 |
| 1422659_at | Camk2d | -3 |
| 1418011_a_at | Sh3glb1 | -3 |
| 1418934_at | Mab21l2 | -3 |
| 1448671_at | --- | -3 |
| 1427293_a_at | Auts2 | -3 |
| 1457065_at | Upk1b | -3 |
| 1417173_at | Atf6b | -3 |
| 1417618_at | Itih2 | -3 |
| 1434740_at | Scarf2 | -3 |
| 1433761_at | Pde4dip | -3 |
| 1456063_at | Fam120c | -3 |
| 1427915_s_at | LOC100045866 /// Tceb1 | -3 |
| 1448433_a_at | Pcolce | -4 |
| 1435547_at | Mkl2 | -4 |
| 1416648_at | Dync1h1 | -4 |
| 1434108_at | Fbxo11 | -4 |
| 1433816_at | Mcart1 | -4 |
| 1435122_x_at | Dnmt1 | -4 |
| 1439506_at | Gm98 | -4 |
| 1451328_at | Pcnxl3 | -4 |
| 1434311_at | Cnot6l | -4 |
| 1448223_at | Fto | -4 |
| 1419551_s_at | Stk39 | -4 |
| 1423065_at | Dnmt3a | -4 |
| 1426976_at | Usp47 | -4 |
| 1455247_at | Amotl1 | -4 |
| 1455406_at | Cask | -4 |
| 1418518_at | Furin | -4 |
| 1428272_at | Eif1b | -4 |
| 1444028_s_at | Dock9 | -4 |
| 1450044_at | Fzd7 | -4 |
| 1454958_at | Gsk3b | -4 |
| 1456783_at | Zdbf2 | -4 |
| 1438665_at | Smpd3 | -4 |
| 1436364_x_at | Nfix | -4 |
| 1448412_a_at | Tsc22d4 | -4 |
| 1456014_s_at | Fermt3 | -4 |
| 1417399_at | Gas6 | -4 |
| 1428229_at | Prkd3 | -4 |
| 1428517_at | Wdfy3 | -4 |
| 1452908_at | Dip2a /// LOC100044059 | -4 |
| 1428466_at | Chd3 | -4 |
| 1435221_at | Foxp1 | -4 |
| 1417011_at | Sdc2 | -4 |
| 1424029_at | Tspyl4 | -4 |
| 1441994_at | Pcdhb16 | -4 |
| 1448705_at | Zbtb22 | -4 |
| 1460009_at | Ier5 | -4 |
| 1433812_at | Lix1l | -4 |
| 1419455_at | Il10rb | -4 |
| 1433434_at | AW551984 | -4 |
| 1437270_a_at | Clcf1 | -4 |
| 1449089_at | Nrip1 | -4 |
| 1455340_at | Dennd5b | -4 |
| 1435137_s_at | 1200015M12Rik /// A130040M12Rik /// E430024C06Rik | -4 |
| 1428011_a_at | Erbb2ip | -4 |
| 1416882_at | Rgs10 | -4 |
| 1441438_at | Gpc6 | -4 |
| 1416532_at | Trrap | -4 |
| 1450065_at | Adcy7 | -4 |
| 1415797_at | Ddr1 | -4 |
| 1457973_at | --- | -4 |
| 1428114_at | Slc14a1 | -4 |
| 1455060_at | G3bp1 | -4 |
| 1452717_at | Slc25a24 | -4 |
| 1438628_x_at | Cntn3 | -4 |
| 1423499_at | Sncaip | -4 |
| 1426727_s_at | Gm2213 /// Gm8801 /// LOC677319 /// Ppp1r10 | -4 |
| 1439665_at | Lpar4 | -4 |
| 1417680_at | Kcna5 | -4 |
| 1450105_at | Adam10 | -4 |
| 1456874_at | Flrt2 | -4 |
| 1423100_at | Fos | -4 |
| 1450054_at | Add1 | -4 |
| 1426531_at | Zmynd11 | -4 |
| 1435260_at | Akt3 | -4 |
| 1418237_s_at | Col18a1 | -4 |
| 1419031_at | Fads2 | -4 |
| 1434797_at | Kirrel | -4 |
| 1453065_at | Aldh5a1 | -4 |
| 1455822_x_at | Surf4 | -4 |
| 1435720_at | Kcnd3 | -4 |
| 1448765_at | Fyn | -4 |
| 1436813_x_at | Khsrp | -4 |
| 1449145_a_at | Cav1 | -4 |
| 1417710_at | Mettl9 | -4 |
| 1423416_at | Smarcc1 | -4 |
| 1423994_at | Kif1b | -4 |
| 1450106_a_at | Evl | -4 |
| 1433624_at | Bat2l | -4 |
| 1448519_at | Tead2 | -4 |
| 1416405_at | Bgn | -4 |
| 1420631_a_at | Blcap | -4 |
| 1423974_at | Numa1 | -4 |
| 1433634_at | Irf2bp2 | -4 |
| 1457032_at | Ak5 | -4 |
| 1416121_at | Lox | -4 |
| 1426541_a_at | Endod1 | -4 |
| 1448929_at | F13a1 | -4 |
| 1456641_at | 1190007F08Rik | -4 |
| 1454752_at | Rbm24 | -4 |
| 1418357_at | Foxg1 | -4 |
| 1433907_at | Pknox2 | -4 |
| 1424996_at | Cflar | -4 |
| 1456475_s_at | Prkar2b | -4 |
| 1416484_at | Ttc3 | -4 |
| 1424062_at | Ube2d1 | -4 |
| 1455607_at | Rspo3 | -4 |
| 1450905_at | Plxnc1 | -4 |
| 1418170_a_at | Zcchc14 | -4 |
| 1450268_at | Fign | -4 |
| 1419483_at | C3ar1 | -4 |
| 1459665_s_at | Mrvi1 | -4 |
| 1434935_at | Aak1 | -4 |
| 1435494_s_at | Dsp | -4 |
| 1439514_at | A930004D18Rik | -4 |
| 1418057_at | Tiam1 | -4 |
| 1415907_at | Ccnd3 | -4 |
| 1428382_at | Smarcc2 | -4 |
| 1450455_s_at | Akr1c12 /// Akr1c13 | -4 |
| 1454960_at | Smad3 | -4 |
| 1455706_at | Stxbp4 | -4 |
| 1423298_at | Add3 | -4 |
| 1417435_at | Large | -4 |
| 1452860_at | Fbxl17 | -4 |
| 1454783_at | Il13ra1 | -4 |
| 1416845_at | Tmem132a | -4 |
| 1436821_at | Plcxd3 | -4 |
| 1425546_a_at | Trf | -4 |
| 1457272_at | --- | -4 |
| 1450732_a_at | Bicd2 | -4 |
| 1427040_at | Mdfic | -4 |
| 1434186_at | Lpar4 | -4 |
| 1435787_at | Ppm1l | -4 |
| 1439906_at | --- | -4 |
| 1441388_at | Mbd2 | -4 |
| 1451119_a_at | Fbln1 | -4 |
| 1435831_at | Upk1b | -4 |
| 1451506_at | Mef2c | -4 |
| 1449630_s_at | Mark1 | -4 |
| 1423852_at | Shisa2 | -4 |
| 1428493_at | Sipa1l3 | -4 |
| 1435059_at | --- | -4 |
| 1438718_at | Fgf9 | -4 |
| 1434459_at | Zc3h4 | -4 |
| 1425484_at | Tox | -4 |
| 1435828_at | Maf | -4 |
| 1424427_at | Tada1 | -4 |
| 1429281_at | 2610008E11Rik | -4 |
| 1438619_x_at | Zdhhc14 | -4 |
| 1456388_at | Atp11a | -4 |
| 1416111_at | Cd83 | -4 |
| 1415937_s_at | Pdcd6ip | -4 |
| 1434869_at | Tdrd3 | -4 |
| 1434788_at | Fzd3 | -4 |
| 1454969_at | Lypd6 | -4 |
| 1424375_s_at | Gimap4 | -4 |
| 1434111_at | LOC100048050 /// Lphn2 | -4 |
| 1424707_at | Tmed10 | -4 |
| 1434589_x_at | Surf4 | -4 |
| 1435056_x_at | Pofut2 | -4 |
| 1427232_at | Tshz1 | -4 |
| 1440167_s_at | Lpp | -4 |
| 1428103_at | Adam10 | -4 |
| 1415877_at | Dpysl3 | -4 |
| 1450728_at | Fjx1 | -4 |
| 1431099_at | Hoxd8 | -4 |
| 1442977_at | --- | -4 |
| 1440238_at | Gltscr1 | -4 |
| 1448361_at | Ttc3 | -4 |
| 1448579_at | Glg1 | -4 |
| 1419155_a_at | Sox4 | -4 |
| 1449303_at | Sesn3 | -4 |
| 1426248_at | Stk24 | -4 |
| 1416598_at | Glis2 | -4 |
| 1426755_at | Ckap4 | -4 |
| 1427902_at | LOC100046744 /// Srrm2 | -4 |
| 1436812_at | Fkrp | -4 |
| 1419428_a_at | Gaa | -4 |
| 1426680_at | Sepn1 | -4 |
| 1451415_at | 1810011O10Rik | -4 |
| 1457198_at | Nrp1 | -4 |
| 1422864_at | Runx1 | -4 |
| 1436125_at | D16Ertd472e | -4 |
| 1418201_at | Plekhg2 | -4 |
| 1457670_s_at | Lmna | -4 |
| 1423448_at | Rab11b | -4 |
| 1419485_at | Foxc1 | -4 |
| 1434446_at | Insr | -4 |
| 1452982_at | Igf1r | -4 |
| 1418183_a_at | Cyth1 | -4 |
| 1452287_at | --- | -4 |
| 1428340_s_at | Atp13a2 | -4 |
| 1458406_at | --- | -4 |
| 1418340_at | Fcer1g | -4 |
| 1419561_at | Ccl3 | -4 |
| 1427769_x_at | Myl3 | -4 |
| 1418901_at | Cebpb | -4 |
| 1421870_at | Trim44 | -4 |
| 1437666_x_at | Ubc | -4 |
| 1427123_s_at | Copg2as2 | -4 |
| 1437885_at | D030029J20Rik | -4 |
| 1439481_at | Ipo9 | -4 |
| 1451037_at | Ptpn9 | -4 |
| 1452035_at | Col4a1 | -4 |
| 1436420_a_at | Ipo4 | -4 |
| 1435777_at | Itpripl2 | -4 |
| 1436839_at | 2010109K11Rik | -4 |
| 1426615_s_at | Ndrg4 | -4 |
| 1433745_at | Trio | -4 |
| 1439106_at | Zfp462 | -4 |
| 1436293_x_at | Ildr2 | -4 |
| 1427048_at | Smo | -4 |
| 1452347_at | Mef2a | -4 |
| 1435340_at | Kdm4a | -4 |
| 1416513_at | Lamb2 | -4 |
| 1455899_x_at | Socs3 | -4 |
| 1424746_at | Kif1c | -4 |
| 1416646_at | Afp | -4 |
| 1428535_at | 9430020K01Rik | -4 |
| 1450241_a_at | Evi2a | -4 |
| 1434875_a_at | Hmgn3 | -4 |
| 1437627_at | Mex3d | -4 |
| 1435884_at | Itsn1 | -4 |
| 1433529_at | Pamr1 | -4 |
| 1460336_at | Ppargc1a | -4 |
| 1419248_at | Rgs2 | -4 |
| 1426448_at | Pja1 | -4 |
| 1417729_at | Myh6 | -4 |
| 1419449_a_at | Gnai2 | -4 |
| 1435493_at | Dsp | -4 |
| 1442006_at | --- | -4 |
| 1438930_s_at | Mecp2 | -4 |
| 1441259_s_at | Ift122 | -4 |
| 1454965_at | Fam171b | -4 |
| 1456423_at | Mbd5 | -4 |
| 1416814_at | Tia1 | -4 |
| 1422489_at | Mogs | -4 |
| 1460694_s_at | Svil | -4 |
| 1428508_at | Tbc1d2b | -4 |
| 1433520_at | Scap | -4 |
| 1448901_at | Cpxm1 | -4 |
| 1439493_at | Zfp827 | -4 |
| 1438245_at | --- | -4 |
| 1459790_x_at | Alx3 | -4 |
| 1426288_at | Lrp4 | -4 |
| 1419833_s_at | Arap3 | -4 |
| 1460620_at | Zfp592 | -4 |
| 1454604_s_at | Tspan12 | -4 |
| 1434043_a_at | Repin1 | -4 |
| 1417727_at | Sfrs9 | -4 |
| 1426911_at | Dsc2 | -4 |
| 1417533_a_at | Itgb5 | -4 |
| 1420518_a_at | Igsf9 | -4 |
| 1416009_at | Tspan3 | -4 |
| 1447883_x_at | Map1lc3a | -4 |
| 1437875_at | Bicd2 | -4 |
| 1424394_at | Selm | -4 |
| 1439044_at | Zfp354c | -4 |
| 1426756_at | Galnt2 | -4 |
| 1458140_at | Slit2 | -4 |
| 1447725_at | C030034E14Rik | -4 |
| 1415890_at | Papss1 | -4 |
| 1452789_at | Snn | -4 |
| 1460464_at | 2700089E24Rik | -4 |
| 1432750_at | Zfp711 | -4 |
| 1457271_at | Cym | -4 |
| 1451545_at | Tdrd3 | -4 |
| 1417234_at | Mmp11 | -4 |
| 1418090_at | Plvap | -4 |
| 1424089_a_at | Tcf4 | -4 |
| 1440346_at | Kdm6b | -4 |
| 1448664_a_at | Speg | -4 |
| 1423902_s_at | Arhgef12 | -4 |
| 1437598_at | --- | -4 |
| 1436459_at | --- | -4 |
| 1418912_at | Plxdc2 | -4 |
| 1433855_at | Abat | -4 |
| 1439610_at | Rab27b | -4 |
| 1435029_at | B230120H23Rik | -4 |
| 1433762_at | C630043F03Rik | -4 |
| 1420565_at | Hoxa1 | -4 |
| 1427112_at | Ttl | -4 |
| 1422293_a_at | Kctd1 | -4 |
| 1456395_at | Ppargc1a | -4 |
| 1443437_at | --- | -4 |
| 1448613_at | Ecm1 | -4 |
| 1455464_x_at | Upk1b | -4 |
| 1422452_at | Bag3 | -4 |
| 1415687_a_at | Psap | -4 |
| 1423999_at | Abl1 | -4 |
| 1455546_s_at | Sf3a2 | -4 |
| 1436030_at | Cachd1 | -4 |
| 1433504_at | Pygb | -4 |
| 1422514_at | Aebp1 | -4 |
| 1435536_at | Ddi2 | -4 |
| 1448732_at | Ctsb | -4 |
| 1420981_a_at | Lmo4 | -4 |
| 1422542_at | Gpr34 | -4 |
| 1438680_at | Auts2 | -4 |
| 1447277_s_at | Pcyox1 | -4 |
| 1427887_at | Rprd1b | -4 |
| 1449353_at | Zmat3 | -4 |
| 1423516_a_at | Nid2 | -4 |
| 1426965_at | Rap2a | -4 |
| 1452032_at | Prkar1a | -4 |
| 1417753_at | Pkd2 | -4 |
| 1436897_at | Mfhas1 | -4 |
| 1416340_a_at | Man2b1 | -4 |
| 1437626_at | Zfp36l2 | -4 |
| 1449335_at | Timp3 | -4 |
| 1422518_at | Cask | -4 |
| 1434128_a_at | Zfp574 | -4 |
| 1438404_at | Rnf144a | -4 |
| 1417214_at | Rab27b | -4 |
| 1460279_a_at | Gtf2i | -4 |
| 1417363_at | Zfp61 | -4 |
| 1417439_at | Cd248 | -4 |
| 1435348_at | D930009K15Rik | -4 |
| 1456067_at | Gli3 | -4 |
| 1456220_at | Fbxl7 | -4 |
| 1417714_x_at | Hba-a1 /// Hba-a2 | -4 |
| 1450621_a_at | Hbb-y | -4 |
| 1424980_s_at | Aph1a | -4 |
| 1418551_at | Mybpc3 | -4 |
| 1420941_at | Rgs5 | -4 |
| 1444980_at | Onecut2 | -4 |
| 1428873_a_at | LOC100047441 /// Msl1 | -4 |
| 1448733_at | Bmi1 | -4 |
| 1424409_at | Cldn23 | -4 |
| 1442434_at | D8Ertd82e | -4 |
| 1447623_s_at | Prkd1 | -4 |
| 1416306_at | Clca3 | -4 |
| 1453009_at | Cpm | -4 |
| 1418158_at | Trp63 | -4 |
| 1460174_at | Dexi | -4 |
| 1423753_at | Bambi | -4 |
| 1434336_s_at | Rcor1 | -4 |
| 1429359_s_at | Rbpms | -4 |
| 1454933_at | Fam176b | -4 |
| 1428026_at | Tshz2 | -4 |
| 1434129_s_at | Lhfpl2 | -4 |
| 1426514_at | Chst15 | -4 |
| 1415768_a_at | Ube2r2 | -4 |
| 1420494_x_at | LOC100048105 /// Ubc | -4 |
| 1437001_at | Gsk3b | -4 |
| 1455288_at | 1110036O03Rik | -4 |
| 1448233_at | Prnp | -4 |
| 1422676_at | Smarce1 | -4 |
| 1425628_a_at | Gtf2i | -4 |
| 1426782_at | Gpr125 | -4 |
| 1429210_at | Col23a1 | -4 |
| 1448204_at | LOC100048168 /// Sav1 | -4 |
| 1459765_s_at | Sf1 | -4 |
| 1416950_at | Tnfaip8 | -4 |
| 1422714_at | Ube2i | -4 |
| 1423941_at | Camk2g /// LOC100045547 | -4 |
| 1434373_at | Fam168a | -4 |
| 1434444_s_at | Anapc1 | -4 |
| 1438169_a_at | Frmd4b | -4 |
| 1419089_at | Timp3 | -4 |
| 1423785_at | Egln1 | -4 |
| 1455979_at | Arid1b | -4 |
| 1448475_at | Olfml3 | -4 |
| 1416840_at | Mid1ip1 | -4 |
| 1423978_at | Sbk1 | -4 |
| 1448026_at | Chd7 | -4 |
| 1454711_at | Trio | -4 |
| 1448827_s_at | Myh6 /// Myh7 | -4 |
| 1418587_at | Traf3 | -4 |
| 1448925_at | Twist2 | -4 |
| 1434825_at | Tnrc18 | -4 |
| 1416048_at | Phc2 | -4 |
| 1428221_at | Klhdc8b | -4 |
| 1442067_at | --- | -4 |
| 1453286_at | Plxna2 | -4 |
| 1435892_at | Asxl3 | -4 |
| 1424010_at | Mfap4 | -4 |
| 1449397_at | Hoxb2 | -4 |
| 1450700_at | Cdc42ep3 | -4 |
| 1416803_at | Fkbp7 | -4 |
| 1428125_at | 4921506J03Rik | -4 |
| 1460044_at | Onecut2 | -4 |
| 1433641_at | LOC100046891 /// Smad5 | -4 |
| 1418128_at | Adcy6 | -4 |
| 1455048_at | Igsf3 | -4 |
| 1434520_at | Sc5d | -4 |
| 1441870_s_at | Pkd2 | -4 |
| 1434258_s_at | Phactr4 | -4 |
| 1455437_at | Sik3 | -4 |
| 1435822_at | D830012I24Rik | -4 |
| 1437639_at | Fam115a | -4 |
| 1423428_at | Ror2 | -4 |
| 1436942_at | A930035D04Rik | -4 |
| 1438058_s_at | Ptov1 | -4 |
| 1449379_at | Kdr | -4 |
| 1434694_at | Lrrc8a | -4 |
| 1431777_a_at | Hmgn3 | -4 |
| 1423854_a_at | Rasl11b | -4 |
| 1438244_at | Nfib | -4 |
| 1452436_at | Loxl2 | -4 |
| 1455993_at | Odz4 | -4 |
| 1425114_at | Rbbp6 | -4 |
| 1452030_a_at | Hnrnpr | -4 |
| 1421142_s_at | Foxp1 | -4 |
| 1428785_at | Amotl1 | -4 |
| 1424572_a_at | H2afy | -4 |
| 1426778_at | Dag1 | -4 |
| 1434026_at | Atp8b2 | -4 |
| 1454650_at | Trim35 | -4 |
| 1448131_at | Mfn2 | -4 |
| 1435227_at | Bcl11b | -4 |
| 1438106_at | --- | -4 |
| 1436188_a_at | Ndrg4 | -4 |
| 1450623_at | Gnb2 | -4 |
| 1420514_at | Tmem47 | -4 |
| 1455507_s_at | Atxn1l | -4 |
| 1422474_at | Pde4b | -4 |
| 1424358_at | Ube2e2 | -4 |
| 1449110_at | Rhob | -4 |
| 1442322_at | --- | -4 |
| 1416852_a_at | Ncdn | -4 |
| 1426363_x_at | H2afy2 | -4 |
| 1450952_at | Pln | -4 |
| 1453266_at | Zbtb4 | -4 |
| 1447927_at | Mpa2l | -4 |
| 1420004_s_at | Pom121 | -4 |
| 1428971_at | Ccny /// LOC100044842 | -4 |
| 1433768_at | Palld | -4 |
| 1453386_at | Tusc1 | -4 |
| 1421140_a_at | Foxp1 | -4 |
| 1434018_at | Fam168b | -4 |
| 1452854_at | Sec63 | -4 |
| 1440739_at | Vegfc | -4 |
| 1429622_at | Cand2 | -4 |
| 1433722_at | Akap13 | -4 |
| 1427185_at | Mef2a | -4 |
| 1427764_a_at | Tcf3 | -4 |
| 1420406_at | Peg12 | -4 |
| 1452380_at | Epha7 | -4 |
| 1422573_at | Ampd3 | -4 |
| 1429293_at | Gpc2 | -4 |
| 1442884_at | Hgf | -4 |
| 1428791_at | Ube2h | -4 |
| 1436449_at | Pcdh11x | -4 |
| 1448893_at | Ncor2 | -4 |
| 1448709_at | Arid1a | -4 |
| 1451244_a_at | Zfp422 | -4 |
| 1415961_at | Itm2c | -4 |
| 1436026_at | Zfp703 | -4 |
| 1423909_at | Tmem176a | -4 |
| 1420822_s_at | Sgpp1 | -4 |
| 1421891_at | St3gal2 | -4 |
| 1416625_at | Serping1 | -4 |
| 1418100_at | A030009H04Rik | -4 |
| 1424099_at | Gpx8 | -4 |
| 1435254_at | Plxnb1 | -4 |
| 1455019_x_at | Ckap4 | -4 |
| 1449514_at | Grk5 | -4 |
| 1450754_at | Cacna2d2 | -4 |
| 1423650_at | Rnf26 | -4 |
| 1421252_a_at | Mef2a | -4 |
| 1449465_at | Reln | -4 |
| 1437277_x_at | Tgm2 | -4 |
| 1429427_s_at | Tcf7l2 | -4 |
| 1449531_at | Leprel2 | -4 |
| 1456735_x_at | Acpl2 | -4 |
| 1441165_s_at | Clstn2 | -4 |
| 1449520_at | Ttc28 | -4 |
| 1452092_at | Chst15 | -4 |
| 1439249_at | Wac | -4 |
| 1457358_at | --- | -4 |
| 1435143_at | Elk3 | -4 |
| 1424970_at | Purg | -4 |
| 1433575_at | Sox4 | -4 |
| 1424015_at | Dennd5a | -4 |
| 1452169_a_at | Dgkz | -4 |
| 1428510_at | Lphn1 | -4 |
| 1433428_x_at | Tgm2 | -4 |
| 1437071_at | Eif1ax | -4 |
| 1415897_a_at | Mgst1 | -4 |
| 1456241_a_at | Slc38a10 | -4 |
| 1452761_a_at | Rbms3 | -4 |
| 1448864_at | Snrk | -4 |
| 1417110_at | Man1a | -4 |
| 1416221_at | Fstl1 | -5 |
| 1415751_at | Hp1bp3 | -5 |
| 1426833_at | Eif4g3 | -5 |
| 1440179_x_at | Rnf217 | -5 |
| 1415943_at | Sdc1 | -5 |
| 1427236_a_at | Mll5 | -5 |
| 1426242_at | Polr2a | -5 |
| 1426955_at | Col18a1 | -5 |
| 1450123_at | Ryr2 | -5 |
| 1418220_at | Foxf2 | -5 |
| 1426642_at | Fn1 | -5 |
| 1429006_s_at | 2610110G12Rik | -5 |
| 1434219_at | Stim2 | -5 |
| 1418049_at | Ltbp3 | -5 |
| 1434310_at | Bmpr2 | -5 |
| 1418723_at | Lpar3 | -5 |
| 1451236_at | Rerg | -5 |
| 1417318_at | Dbc1 | -5 |
| 1420505_a_at | Stxbp1 | -5 |
| 1440007_at | D930003E18Rik | -5 |
| 1428922_at | 1200009O22Rik | -5 |
| 1425678_a_at | Snrk | -5 |
| 1426799_at | Rab8b | -5 |
| 1435176_a_at | Id2 | -5 |
| 1448898_at | Ccl9 | -5 |
| 1419584_at | Ttc28 | -5 |
| 1429089_s_at | 2900026A02Rik | -5 |
| 1453247_at | Zfp618 | -5 |
| 1437291_at | 2700081O15Rik | -5 |
| 1427151_at | Qser1 | -5 |
| 1444611_at | --- | -5 |
| 1433787_at | Nell1 | -5 |
| 1435477_s_at | Fcgr2b | -5 |
| 1433647_s_at | Rhobtb3 | -5 |
| 1450062_a_at | Maged1 | -5 |
| 1427294_a_at | Slc38a10 | -5 |
| 1426897_at | Rcc2 | -5 |
| 1433489_s_at | Fgfr2 | -5 |
| 1453015_at | 4933407C03Rik | -5 |
| 1435167_at | Ranbp6 | -5 |
| 1436866_at | Efna5 | -5 |
| 1417517_at | Plagl2 | -5 |
| 1436714_at | Lpp | -5 |
| 1439755_at | Sipa1l1 | -5 |
| 1449530_at | Trps1 | -5 |
| 1435078_at | Tanc2 | -5 |
| 1423584_at | Igfbp7 | -5 |
| 1417184_s_at | Hbb-b1 /// Hbb-b2 | -5 |
| 1442214_at | --- | -5 |
| 1448670_at | Ube2e3 | -5 |
| 1449280_at | Esm1 | -5 |
| 1456135_s_at | Pxn | -5 |
| 1436960_at | Brd3 | -5 |
| 1451691_at | Ednra | -5 |
| 1455034_at | Nr4a2 | -5 |
| 1420621_a_at | App | -5 |
| 1430164_a_at | Grb10 | -5 |
| 1434005_at | Rbms1 | -5 |
| 1422537_a_at | Id2 | -5 |
| 1434683_at | --- | -5 |
| 1426614_at | Zmynd8 | -5 |
| 1455900_x_at | Tgm2 | -5 |
| 1455729_at | Gnaq | -5 |
| 1419417_at | Vegfc | -5 |
| 1422124_a_at | Ptprc | -5 |
| 1436954_at | Wipf1 | -5 |
| 1421182_at | Clec1b | -5 |
| 1435396_at | Stxbp6 | -5 |
| 1451154_a_at | Celf2 | -5 |
| 1438316_a_at | Ccdc102a | -5 |
| 1436948_a_at | Fam70a | -5 |
| 1459250_at | Tshz2 | -5 |
| 1434776_at | Sema5a | -5 |
| 1450843_a_at | Serpinh1 | -5 |
| 1454613_at | Dpysl3 | -5 |
| 1454673_at | Wasf2 | -5 |
| 1415702_a_at | Ctbp1 /// LOC100045360 | -5 |
| 1424086_at | Oaf | -5 |
| 1455040_s_at | Nhsl2 | -5 |
| 1437422_at | Sema5a | -5 |
| 1434442_at | Stbd1 | -5 |
| 1417455_at | Tgfb3 | -5 |
| 1451105_at | Vash2 | -5 |
| 1460292_a_at | Smarca1 | -5 |
| 1451418_a_at | Spsb4 | -5 |
| 1420693_at | Myom1 | -5 |
| 1444269_at | --- | -5 |
| 1427298_at | Dnm3os | -5 |
| 1443609_s_at | Syvn1 | -5 |
| 1437690_x_at | Csnk1d | -5 |
| 1448259_at | Fstl1 | -5 |
| 1452156_a_at | Nisch | -5 |
| 1416731_at | Top2b | -5 |
| 1437008_x_at | Tmem109 | -5 |
| 1428938_at | Gnaq | -5 |
| 1438559_x_at | Slc44a2 | -5 |
| 1424050_s_at | Fgfr1 | -5 |
| 1434678_at | Mbnl3 | -5 |
| 1436153_a_at | Zmynd11 | -5 |
| 1416914_s_at | Mtvr2 | -5 |
| 1418142_at | Kcnj8 | -5 |
| 1436185_at | AI314180 | -5 |
| 1456013_x_at | Slc35a4 | -5 |
| 1454200_at | Zeb2 | -5 |
| 1416693_at | Foxc2 | -5 |
| 1428122_s_at | Fam125b | -5 |
| 1448995_at | Pf4 | -5 |
| 1422667_at | Krt15 | -5 |
| 1416723_at | Tcf4 | -5 |
| 1450990_at | Gpc3 | -5 |
| 1428914_at | Sh3pxd2a | -5 |
| 1417936_at | Ccl9 | -5 |
| 1455826_a_at | Bace1 | -5 |
| 1416468_at | Aldh1a1 | -5 |
| 1452281_at | Sos2 | -5 |
| 1417262_at | Ptgs2 | -5 |
| 1424852_at | Mef2c | -5 |
| 1456377_x_at | Limd2 | -5 |
| 1429151_at | Dcaf7 | -5 |
| 1435256_at | Clip3 | -5 |
| 1437845_x_at | Pofut2 | -5 |
| 1435018_at | 5930434B04Rik | -5 |
| 1436993_x_at | Pfn2 | -5 |
| 1439381_x_at | Marveld1 | -5 |
| 1417104_at | Emp3 | -5 |
| 1433956_at | Cdh5 | -5 |
| 1452265_at | Clasp1 | -5 |
| 1456028_x_at | Marcks | -5 |
| 1448754_at | Rbp1 | -5 |
| 1423851_a_at | Shisa2 | -5 |
| 1425896_a_at | Fbn1 | -5 |
| 1426819_at | Hipk3 | -5 |
| 1423594_a_at | Ednrb | -5 |
| 1417962_s_at | Ghr | -5 |
| 1436044_at | Scn7a | -5 |
| 1451086_s_at | Rac1 | -5 |
| 1418545_at | Wasf1 | -5 |
| 1439108_at | Mll5 | -5 |
| 1425652_s_at | LOC100044395 /// Rbpms | -5 |
| 1442041_at | LOC552876 | -5 |
| 1421840_at | Abca1 | -5 |
| 1433488_x_at | Gns | -5 |
| 1438688_at | Srrm2 | -5 |
| 1431751_a_at | Mpped2 | -5 |
| 1434149_at | Tcf4 | -5 |
| 1435649_at | Nexn | -5 |
| 1452127_a_at | Ptpn13 | -5 |
| 1435064_a_at | Tmem27 | -5 |
| 1435399_at | Synpo2 | -5 |
| 1417266_at | Ccl6 | -5 |
| 1418467_at | Smarcd3 | -5 |
| 1417852_x_at | Clca1 | -5 |
| 1421965_s_at | Notch3 | -5 |
| 1450199_a_at | Stab1 | -5 |
| 1441620_at | --- | -5 |
| 1438030_at | Rasgrp3 | -5 |
| 1439760_x_at | Upk1b | -5 |
| 1451542_at | Ssbp2 | -5 |
| 1435763_at | Tbc1d16 | -5 |
| 1457568_at | Hnrnpd | -5 |
| 1440819_s_at | Szt2 | -5 |
| 1436043_at | Scn7a | -5 |
| 1438231_at | Foxp2 | -5 |
| 1430038_at | Gphn | -5 |
| 1429896_at | 5830408B19Rik | -5 |
| 1417597_at | Cd28 | -5 |
| 1427670_a_at | Tcf12 | -5 |
| 1454666_at | LOC100046855 | -5 |
| 1423828_at | Fasn | -5 |
| 1426794_at | Ptprs | -5 |
| 1415922_s_at | Marcksl1 | -5 |
| 1448148_at | Grn | -5 |
| 1449815_a_at | Ssbp2 | -5 |
| 1429559_at | Gnaq | -5 |
| 1418282_x_at | Serpina1b | -5 |
| 1418894_s_at | Pbx2 | -5 |
| 1423488_at | Mmd | -5 |
| 1434148_at | Tcf4 | -5 |
| 1456057_x_at | Tmem109 | -5 |
| 1419554_at | Cd47 | -5 |
| 1418532_at | Fzd2 | -5 |
| 1424922_a_at | Brd4 | -5 |
| 1423287_at | Cbln1 | -5 |
| 1460302_at | Thbs1 | -5 |
| 1422449_s_at | Rcn2 | -5 |
| 1427186_a_at | Mef2a | -5 |
| 1419592_at | Unc5c | -5 |
| 1425016_at | Ephb2 | -5 |
| 1425582_a_at | Emcn | -5 |
| 1457042_at | AI256396 | -5 |
| 1422742_at | Hivep1 | -5 |
| 1424594_at | Samd4 | -5 |
| 1423489_at | LOC100047565 /// Mmd | -5 |
| 1456789_at | Zfp462 | -5 |
| 1448405_a_at | Eid1 | -5 |
| 1417491_at | Ctsb | -5 |
| 1439556_at | Ncam1 | -5 |
| 1424051_at | Col4a2 | -5 |
| 1448592_at | Crtap | -5 |
| 1426297_at | Tcf3 | -5 |
| 1438007_at | Fam19a2 | -5 |
| 1416700_at | Rnd3 | -5 |
| 1454882_at | L3mbtl3 | -5 |
| 1426769_s_at | Maml1 | -5 |
| 1438861_at | Bnc2 | -5 |
| 1416808_at | Nid1 | -5 |
| 1443832_s_at | Sdpr | -5 |
| 1428402_at | Zcchc3 | -5 |
| 1450736_a_at | Hbb-bh1 | -5 |
| 1434667_at | Col8a2 | -5 |
| 1417644_at | Sspn | -5 |
| 1428407_at | Hnrnpa0 | -5 |
| 1426804_at | Smarca4 | -5 |
| 1451990_at | Mapre2 | -5 |
| 1454862_at | Phldb2 | -5 |
| 1427917_s_at | Ssbp3 | -6 |
| 1416657_at | Akt1 | -6 |
| 1418168_at | Zcchc14 | -6 |
| 1427677_a_at | Sox6 | -6 |
| 1451285_at | Fus | -6 |
| 1455242_at | Foxp1 | -6 |
| 1454997_at | Msrb3 | -6 |
| 1453012_at | Tsc22d2 | -6 |
| 1431004_at | Loxl2 | -6 |
| 1446048_at | --- | -6 |
| 1439732_at | --- | -6 |
| 1436692_at | E130308A19Rik | -6 |
| 1454867_at | Mn1 | -6 |
| 1460125_at | Ccdc141 | -6 |
| 1456739_x_at | Armcx2 | -6 |
| 1436343_at | Chd4 | -6 |
| 1429459_at | Sema3d | -6 |
| 1456811_at | Cxxc4 | -6 |
| 1447915_x_at | Tmem204 | -6 |
| 1450117_at | Tcf7l1 | -6 |
| 1451888_a_at | Odz4 | -6 |
| 1419249_at | Cdk14 | -6 |
| 1419598_at | Ms4a6d | -6 |
| 1416514_a_at | Fscn1 | -6 |
| 1459211_at | Gli2 | -6 |
| 1441948_x_at | Zfand3 | -6 |
| 1448617_at | Cd53 | -6 |
| 1455505_at | Gatad2a | -6 |
| 1459850_x_at | Glrb | -6 |
| 1425506_at | Mylk | -6 |
| 1453622_s_at | Mllt3 | -6 |
| 1455638_at | Zfp319 | -6 |
| 1454741_s_at | Tmem164 | -6 |
| 1437247_at | Fosl2 /// LOC634417 | -6 |
| 1435349_at | Nrp2 | -6 |
| 1419638_at | Efnb2 | -6 |
| 1420859_at | Pkia | -6 |
| 1456733_x_at | Serpinh1 | -6 |
| 1419156_at | Sox4 | -6 |
| 1433691_at | Ppp1r3c | -6 |
| 1457687_at | Bcl2 | -6 |
| 1450429_at | Capn6 | -6 |
| 1435224_at | Crebbp | -6 |
| 1451801_at | Trdn | -6 |
| 1458341_x_at | Pde5a | -6 |
| 1459713_s_at | Ano1 | -6 |
| 1428911_at | Ttll4 | -6 |
| 1431339_a_at | Efhd2 | -6 |
| 1434112_at | LOC100048050 /// Lphn2 | -6 |
| 1428136_at | Sfrp1 | -6 |
| 1437279_x_at | Sdc1 | -6 |
| 1452700_s_at | Kbtbd7 | -6 |
| 1433857_at | Fat1 | -6 |
| 1423680_at | Fads1 | -6 |
| 1417087_at | Glg1 | -6 |
| 1455862_at | Ubtd2 | -6 |
| 1455154_at | Gli3 | -6 |
| 1416008_at | Satb1 | -6 |
| 1454838_s_at | Pkdcc | -6 |
| 1428280_at | Fip1l1 | -6 |
| 1435337_at | Tshz3 | -6 |
| 1439107_a_at | Mll5 | -6 |
| 1438118_x_at | Vim | -6 |
| 1451075_s_at | Ctdsp2 | -6 |
| 1423359_at | Pln | -6 |
| 1434188_at | Slc16a12 | -6 |
| 1418210_at | Pfn2 | -6 |
| 1419693_at | Colec12 | -6 |
| 1439496_at | Ston1 | -6 |
| 1450186_s_at | Gnas | -6 |
| 1428284_at | 8430427H17Rik | -6 |
| 1448429_at | Gyg | -6 |
| 1438625_s_at | Cdk16 | -6 |
| 1416007_at | Satb1 | -6 |
| 1418893_at | Pbx2 | -6 |
| 1456567_x_at | Grn | -6 |
| 1439944_at | --- | -6 |
| 1416638_at | Sall2 | -6 |
| 1437990_x_at | Hbb-bh1 | -6 |
| 1421612_a_at | H2afy2 /// H2afy3 | -6 |
| 1417381_at | C1qa | -6 |
| 1458364_s_at | Tor1aip1 | -6 |
| 1452666_a_at | Tmcc2 | -6 |
| 1454729_at | LOC100045503 /// Tmem108 | -6 |
| 1455724_at | Prrg1 | -6 |
| 1441727_s_at | Zfp467 | -6 |
| 1455206_at | Rps6ka3 | -6 |
| 1460259_s_at | Clca1 /// Clca2 | -6 |
| 1436403_at | Fam171a2 | -6 |
| 1438971_x_at | Ube2h | -6 |
| 1430388_a_at | Sulf2 | -6 |
| 1455851_at | Bmp5 | -6 |
| 1428875_at | Golim4 | -6 |
| 1448471_a_at | Ctla2a | -6 |
| 1456710_at | Pcdhb11 | -6 |
| 1416072_at | Cd34 | -6 |
| 1417018_at | Efemp2 | -6 |
| 1436627_at | D17Ertd663e | -6 |
| 1419599_s_at | Ms4a6d | -6 |
| 1418918_at | Igfbp1 | -6 |
| 1436917_s_at | Gpsm1 | -6 |
| 1439066_at | Angpt1 | -6 |
| 1416390_at | Rcbtb2 | -6 |
| 1415850_at | Rasa3 | -6 |
| 1451513_x_at | Serpina1a /// Serpina1b | -6 |
| 1450932_s_at | Dock9 | -6 |
| 1424443_at | Tm6sf1 | -6 |
| 1418095_at | Smpx | -6 |
| 1426604_at | Rnasel | -6 |
| 1428192_at | Kbtbd7 | -6 |
| 1438629_x_at | Grn | -6 |
| 1447602_x_at | Sulf2 | -6 |
| 1420842_at | Ptprf | -6 |
| 1437908_a_at | Ergic1 | -6 |
| 1439990_at | --- | -6 |
| 1418285_at | Efnb1 | -6 |
| 1436994_a_at | Hist1h1c | -6 |
| 1418941_at | Pcdhb22 | -6 |
| 1435285_at | Mpped2 | -6 |
| 1422504_at | Glrb | -6 |
| 1436939_at | Unc45b | -6 |
| 1428401_at | Zcchc3 | -6 |
| 1456022_at | Hipk2 | -6 |
| 1434378_a_at | Mxd4 | -6 |
| 1430637_at | 2210016H18Rik | -6 |
| 1433920_at | Sema4c | -6 |
| 1438452_at | Nebl | -6 |
| 1440169_x_at | Ifnar2 | -6 |
| 1454926_at | Sphkap | -6 |
| 1437339_s_at | Pcsk5 | -6 |
| 1455915_at | Galnt4 | -6 |
| 1418733_at | Twist1 | -6 |
| 1451277_at | Zadh2 | -6 |
| 1416101_a_at | Hist1h1c | -6 |
| 1455591_at | Zfp618 | -6 |
| 1460004_x_at | Stx6 | -6 |
| 1456195_x_at | Itgb5 | -6 |
| 1421923_at | Sh3bp5 | -6 |
| 1436869_at | Shh | -6 |
| 1427183_at | Efemp1 | -6 |
| 1459823_at | Ehd2 | -6 |
| 1449522_at | Unc5c | -6 |
| 1418599_at | Col11a1 | -6 |
| 1459522_s_at | Gyg | -6 |
| 1426306_a_at | LOC100046560 /// Maged2 | -6 |
| 1448460_at | Acvr1 | -6 |
| 1434089_at | Synpo | -6 |
| 1449188_at | Midn | -6 |
| 1433924_at | Peg3 | -6 |
| 1449244_at | Cdh2 | -6 |
| 1455556_at | Notch2 | -6 |
| 1424130_a_at | Ptrf | -6 |
| 1426640_s_at | Trib2 | -6 |
| 1437353_at | Setd1b | -6 |
| 1437478_s_at | Efhd2 | -6 |
| 1442873_at | --- | -6 |
| 1419380_at | Zfp423 | -6 |
| 1424842_a_at | Arhgap24 | -6 |
| 1448201_at | Sfrp2 | -6 |
| 1429205_at | Mllt3 | -6 |
| 1421163_a_at | Nfia | -6 |
| 1434848_at | --- | -6 |
| 1438855_x_at | Tnfaip2 | -6 |
| 1442063_at | Adamtsl1 | -6 |
| 1451342_at | Spon1 | -6 |
| 1429621_at | Cand2 | -6 |
| 1417466_at | Rgs5 | -6 |
| 1448655_at | Lrp1 | -6 |
| 1426998_at | Zfand3 | -6 |
| 1448694_at | Jun | -6 |
| 1437226_x_at | Marcksl1 | -7 |
| 1437165_a_at | Pcolce | -7 |
| 1420821_at | Sgpp1 | -7 |
| 1441811_x_at | Tmem176a | -7 |
| 1428647_at | LOC676870 /// Pbx1 | -7 |
| 1448406_at | Eid1 | -7 |
| 1417847_at | Ulk2 | -7 |
| 1444432_at | D330040H18Rik | -7 |
| 1438423_at | Ssbp2 | -7 |
| 1439265_at | --- | -7 |
| 1453208_at | 2700089E24Rik | -7 |
| 1419411_at | Tac2 | -7 |
| 1431225_at | --- | -7 |
| 1452398_at | Plce1 | -7 |
| 1417447_at | Tcf21 | -7 |
| 1433783_at | Ldb3 | -7 |
| 1418496_at | Foxa1 | -7 |
| 1456307_s_at | Adcy7 | -7 |
| 1427108_at | 9530068E07Rik | -7 |
| 1433854_at | Tmem164 | -7 |
| 1439766_x_at | Vegfc | -7 |
| 1434809_at | Arhgap28 | -7 |
| 1423975_s_at | Numa1 | -7 |
| 1426805_at | Smarca4 | -7 |
| 1448553_at | Myh7 | -7 |
| 1427086_at | Slit3 | -7 |
| 1434802_s_at | Ntf3 | -7 |
| 1420394_s_at | Gp49a /// Lilrb4 | -7 |
| 1426621_a_at | Ppp2r2b | -7 |
| 1455037_at | Plxna2 | -7 |
| 1419186_a_at | St8sia4 | -7 |
| 1423250_a_at | Tgfb2 | -7 |
| 1416168_at | Serpinf1 | -7 |
| 1419486_at | Foxc1 | -7 |
| 1439915_at | --- | -7 |
| 1433474_at | Edil3 | -7 |
| 1448620_at | Fcgr3 | -7 |
| 1453351_at | Tbx20 | -7 |
| 1423484_at | Bicc1 | -7 |
| 1437785_at | Adamts9 | -7 |
| 1458268_s_at | Igfbp3 | -7 |
| 1415971_at | Marcks | -7 |
| 1428939_s_at | Gnaq | -7 |
| 1437284_at | Fzd1 | -7 |
| 1428967_at | Igf1r | -7 |
| 1425995_s_at | Wt1 | -7 |
| 1455817_x_at | Zxdb | -7 |
| 1423717_at | Ak3 | -7 |
| 1451475_at | Plxnd1 | -7 |
| 1435203_at | Man2a2 | -7 |
| 1448124_at | Gusb | -7 |
| 1436475_at | Nr2f2 | -7 |
| 1421433_at | Zfhx4 | -7 |
| 1454978_at | Ttyh3 | -7 |
| 1427982_s_at | Syne2 | -7 |
| 1436824_x_at | Rnf26 | -7 |
| 1436916_at | Tmem108 | -7 |
| 1453002_at | Sox11 | -7 |
| 1417111_at | Man1a | -7 |
| 1428187_at | Cd47 | -7 |
| 1427320_at | Copg2as2 | -7 |
| 1431890_a_at | Mllt3 | -7 |
| 1425814_a_at | Calcrl | -7 |
| 1415780_a_at | Armcx2 | -7 |
| 1428872_at | LOC100047441 /// Msl1 | -7 |
| 1436029_at | Bicc1 | -7 |
| 1417612_at | Ier5 | -7 |
| 1418012_at | Sh3glb1 | -7 |
| 1423757_x_at | Igfbp4 | -7 |
| 1426283_at | Ntm | -7 |
| 1434924_at | Phf2 | -7 |
| 1416342_at | Tnc | -7 |
| 1455970_at | Pde5a | -7 |
| 1424261_at | Zfp672 | -7 |
| 1437101_at | Lats2 | -7 |
| 1424040_at | Mtap7d1 | -7 |
| 1438232_at | Foxp2 | -7 |
| 1453120_at | Tmx4 | -7 |
| 1433453_a_at | Abtb2 | -7 |
| 1452250_a_at | Col6a2 | -7 |
| 1427886_at | Pom121 | -7 |
| 1434100_x_at | Ppargc1a | -7 |
| 1427231_at | Robo1 | -7 |
| 1429189_at | Arsb | -7 |
| 1423110_at | Col1a2 | -7 |
| 1428861_at | Filip1l | -7 |
| 1450857_a_at | Col1a2 | -7 |
| 1449401_at | C1qc | -7 |
| 1416811_s_at | Ctla2a /// Ctla2b | -7 |
| 1427442_a_at | App | -7 |
| 1416985_at | Sirpa | -7 |
| 1452251_at | Nbea | -7 |
| 1455854_a_at | Ssh1 | -7 |
| 1437239_x_at | Phc2 | -7 |
| 1415825_s_at | Csnk1d | -7 |
| 1433716_x_at | Gfra2 | -7 |
| 1443201_at | --- | -7 |
| 1417409_at | Jun | -7 |
| 1422545_at | Tbx2 | -7 |
| 1417355_at | Peg3 | -7 |
| 1427167_at | Armcx4 | -7 |
| 1436223_at | Itgb8 | -7 |
| 1421141_a_at | Foxp1 | -7 |
| 1452220_at | Dock1 | -7 |
| 1421193_a_at | Pbx3 | -7 |
| 1427168_a_at | Col14a1 | -7 |
| 1452740_at | Myh10 | -8 |
| 1450784_at | Reck | -8 |
| 1438941_x_at | Ampd2 | -8 |
| 1434413_at | Igf1 | -8 |
| 1429005_at | Mfhas1 | -8 |
| 1440431_at | --- | -8 |
| 1420872_at | Gucy1b3 | -8 |
| 1425464_at | Gata6 | -8 |
| 1418533_s_at | Fzd2 | -8 |
| 1422889_at | Pcdh18 | -8 |
| 1415823_at | Scd2 | -8 |
| 1434316_at | Chsy1 /// LOC100047167 | -8 |
| 1449020_at | Plscr3 | -8 |
| 1457139_at | Auts2 | -8 |
| 1418534_at | Fzd2 | -8 |
| 1447655_x_at | Sox6 | -8 |
| 1448494_at | Gas1 | -8 |
| 1427049_s_at | Smo | -8 |
| 1425475_at | Col4a5 | -8 |
| 1416904_at | Mbnl1 | -8 |
| 1423071_x_at | 9930105H17Rik /// LOC381508 | -8 |
| 1448395_at | Sfrp1 | -8 |
| 1434099_at | Ppargc1a | -8 |
| 1434285_at | Frmd4a | -8 |
| 1440634_at | --- | -8 |
| 1452381_at | Creb3l2 | -8 |
| 1436791_at | Wnt5a | -8 |
| 1423062_at | Igfbp3 | -8 |
| 1427446_s_at | Ttn | -8 |
| 1434479_at | Col5a1 | -8 |
| 1440935_at | --- | -8 |
| 1429051_s_at | Sox11 | -8 |
| 1455447_at | D430019H16Rik | -8 |
| 1460187_at | Sfrp1 | -8 |
| 1452412_at | Hoxc8 | -8 |
| 1434728_at | Gria3 | -8 |
| 1449583_at | Pcdhb20 | -8 |
| 1423104_at | Irs1 | -8 |
| 1435321_at | Limch1 | -8 |
| 1433662_s_at | Timp2 | -8 |
| 1435888_at | Egfr | -8 |
| 1428910_at | 2310022B05Rik | -8 |
| 1434645_at | C530008M17Rik | -8 |
| 1453070_at | Pcdh17 | -8 |
| 1436600_at | Tox3 | -8 |
| 1450924_at | Hdgfrp3 | -8 |
| 1416160_at | Nr2f2 | -8 |
| 1429951_at | Ssbp2 | -8 |
| 1422552_at | Rprm | -8 |
| 1436570_at | --- | -8 |
| 1416614_at | Eid1 | -8 |
| 1448593_at | Wisp1 | -8 |
| 1415822_at | Scd2 | -8 |
| 1449632_s_at | Fkbp10 | -8 |
| 1424674_at | Slc39a6 | -8 |
| 1418209_a_at | Pfn2 | -8 |
| 1421992_a_at | Igfbp4 | -8 |
| 1436736_x_at | D0H4S114 | -8 |
| 1426864_a_at | Ncam1 | -8 |
| 1422751_at | Tle1 | -8 |
| 1435977_at | Hdgfrp3 | -8 |
| 1427961_s_at | Ugt2b34 | -8 |
| 1434237_at | Upk3b | -8 |
| 1455249_at | Slc36a4 | -8 |
| 1438413_at | Senp7 | -8 |
| 1460436_at | Ndst1 | -8 |
| 1460359_at | Armcx3 | -9 |
| 1437853_x_at | Ndn | -9 |
| 1448383_at | Mmp14 | -9 |
| 1416114_at | Sparcl1 | -9 |
| 1420843_at | Ptprf | -9 |
| 1434856_at | Ankrd44 | -9 |
| 1450931_at | Dock9 | -9 |
| 1422018_at | Hivep2 | -9 |
| 1447720_x_at | Prkaca | -9 |
| 1456610_at | Kdm6b | -9 |
| 1436115_at | Gm266 | -9 |
| 1421045_at | Mrc2 | -9 |
| 1434895_s_at | Ppp1r13b | -9 |
| 1423213_at | Plxnc1 | -9 |
| 1420798_s_at | Pcdha1 /// Pcdha10 /// Pcdha11 /// Pcdha12 /// Pcdha2 /// Pcdha3 /// Pcdha4 /// Pcdha5 /// Pcdha6 /// Pcdha7 /// Pcdha8 /// Pcdha9 /// Pcdhac1 /// Pcdhac2 | -9 |
| 1419639_at | Efnb2 | -9 |
| 1428259_at | Pxdn | -9 |
| 1421172_at | Adam12 | -9 |
| 1448152_at | Igf2 | -9 |
| 1455940_x_at | Wdr6 | -9 |
| 1452382_at | Dnm3os | -9 |
| 1418826_at | Ms4a6b | -9 |
| 1415972_at | Marcks | -9 |
| 1439852_at | --- | -9 |
| 1456214_at | Pcdh7 | -9 |
| 1434141_at | Gucy1a3 | -9 |
| 1447861_x_at | Meis2 | -9 |
| 1460006_at | Zfhx3 | -9 |
| 1435456_at | Ttc28 | -9 |
| 1436983_at | Crebbp | -9 |
| 1423063_at | Dnmt3a | -9 |
| 1452107_s_at | Npnt | -9 |
| 1452792_at | Dzip1 | -9 |
| 1448591_at | Ctss | -9 |
| 1418370_at | Tnnc1 | -9 |
| 1438532_at | Hmcn1 | -9 |
| 1434286_at | Trps1 | -9 |
| 1452280_at | Farp1 | -9 |
| 1454906_at | Rarb | -9 |
| 1429809_at | Tmtc2 | -9 |
| 1425260_at | Alb | -9 |
| 1456292_a_at | Vim | -9 |
| 1438312_s_at | Ltbp3 | -9 |
| 1416414_at | Emilin1 | -9 |
| 1448933_at | Pcdhb17 | -9 |
| 1447849_s_at | Maf | -9 |
| 1424382_at | Rcn3 | -9 |
| 1428367_at | Ndst1 | -9 |
| 1456756_at | Zc3hav1l | -9 |
| 1423669_at | Col1a1 | -9 |
| 1448870_at | Ltbp1 | -9 |
| 1417356_at | Peg3 | -9 |
| 1416473_a_at | Igdcc4 | -9 |
| 1434704_at | Mll5 | -9 |
| 1451190_a_at | Sbk1 | -9 |
| 1433795_at | Tgfbr3 | -10 |
| 1417625_s_at | Cxcr7 | -10 |
| 1450641_at | Vim | -10 |
| 1436905_x_at | Laptm5 | -10 |
| 1429206_at | Rhobtb1 | -10 |
| 1418815_at | Cdh2 | -10 |
| 1422771_at | Smad6 | -10 |
| 1419589_at | Cd93 | -10 |
| 1447500_at | Cux2 | -10 |
| 1450922_a_at | Tgfb2 | -10 |
| 1435222_at | Foxp1 | -10 |
| 1439441_x_at | Lats2 | -10 |
| 1452404_at | Phactr2 | -10 |
| 1450644_at | Zfp36l1 | -10 |
| 1435537_at | Ptprd | -10 |
| 1429379_at | Lyve1 | -10 |
| 1445268_at | --- | -10 |
| 1424886_at | Ptprd | -10 |
| 1441667_s_at | Smyd1 | -10 |
| 1416122_at | Ccnd2 | -10 |
| 1420649_at | Zfhx3 | -10 |
| 1427640_a_at | Runx1t1 | -10 |
| 1423812_s_at | Vopp1 | -10 |
| 1417505_s_at | Gm13305 /// Gm2002 /// Il11ra1 /// Il11ra2 | -10 |
| 1438081_at | Mcc | -10 |
| 1452106_at | Npnt | -10 |
| 1457966_at | --- | -10 |
| 1435820_x_at | Ddr1 | -10 |
| 1457038_at | Frem2 | -10 |
| 1450716_at | Adamts1 | -10 |
| 1448748_at | Plek | -10 |
| 1439019_at | Fras1 | -10 |
| 1447864_s_at | Pogk | -10 |
| 1450047_at | Hs6st2 | -10 |
| 1427257_at | Vcan | -10 |
| 1453055_at | Sema6d | -10 |
| 1416761_at | Hsd11b2 | -10 |
| 1440091_at | Meis2 | -10 |
| 1448316_at | Cmtm3 /// LOC100046883 | -10 |
| 1433977_at | Hs3st3b1 | -10 |
| 1452352_at | Ctla2b | -10 |
| 1436959_x_at | Nelf | -10 |
| 1436515_at | Bach2 | -10 |
| 1429088_at | Lbh | -10 |
| 1450430_at | Mrc1 | -10 |
| 1448785_at | Runx1t1 | -10 |
| 1418317_at | Lhx2 | -10 |
| 1442082_at | C3ar1 | -10 |
| 1422528_a_at | Zfp36l1 | -10 |
| 1416649_at | Ambp | -11 |
| 1450923_at | Tgfb2 | -11 |
| 1419093_at | Tdo2 | -11 |
| 1417649_at | Cdkn1c | -11 |
| 1419406_a_at | Bcl11a | -11 |
| 1433512_at | Fli1 | -11 |
| 1451161_a_at | Emr1 | -11 |
| 1416474_at | Igdcc4 | -11 |
| 1423608_at | Itm2a | -11 |
| 1456700_x_at | Marcks | -11 |
| 1422536_at | Tnni3 | -11 |
| 1417023_a_at | Fabp4 | -11 |
| 1451978_at | Loxl1 | -11 |
| 1421027_a_at | Mef2c | -11 |
| 1420928_at | St6gal1 | -11 |
| 1424111_at | Igf2r | -11 |
| 1433743_at | Dach1 | -11 |
| 1418601_at | Aldh1a7 | -11 |
| 1456229_at | Hoxb3 | -11 |
| 1438325_at | Mecom | -11 |
| 1429372_at | Sox11 | -11 |
| 1418004_a_at | Tmem176b | -11 |
| 1419922_s_at | Atrnl1 | -11 |
| 1452670_at | Myl9 | -11 |
| 1439557_s_at | Ldb2 | -11 |
| 1426300_at | Alcam | -11 |
| 1434745_at | Ccnd2 | -11 |
| 1423072_at | 6720475J19Rik | -11 |
| 1440990_at | Kif26b | -11 |
| 1423294_at | Mest | -12 |
| 1437197_at | Sorbs2 | -12 |
| 1437401_at | Igf1 | -12 |
| 1427076_at | Mpeg1 | -12 |
| 1416779_at | Sdpr | -12 |
| 1420512_at | Dkk2 | -12 |
| 1436790_a_at | Sox11 | -12 |
| 1429052_at | Ptprd | -12 |
| 1437442_at | Pcdh7 | -12 |
| 1450839_at | D0H4S114 | -12 |
| 1443847_x_at | Aff2 | -12 |
| 1436546_at | Lix1l | -12 |
| 1446921_at | --- | -12 |
| 1453125_at | Sox11 | -12 |
| 1456087_at | Nfia | -12 |
| 1415931_at | Igf2 | -12 |
| 1428433_at | Hipk2 | -12 |
| 1419872_at | Csf1r | -12 |
| 1420650_at | Zfhx3 | -12 |
| 1426766_at | 6330403K07Rik | -12 |
| 1434920_a_at | Evl | -12 |
| 1424112_at | Igf2r | -12 |
| 1428097_at | 2510009E07Rik | -12 |
| 1416123_at | Ccnd2 | -12 |
| 1454677_at | Timp2 | -12 |
| 1417492_at | Ctsb | -12 |
| 1427960_at | Ugt2b34 | -13 |
| 1417937_at | Dact1 | -13 |
| 1419663_at | Ogn | -13 |
| 1443926_at | --- | -13 |
| 1435385_at | Tshz2 | -13 |
| 1425603_at | Tmem176a | -13 |
| 1435087_at | Zfp362 | -13 |
| 1418664_at | Mpdz | -13 |
| 1448590_at | Col6a1 | -13 |
| 1429178_at | Odz3 | -13 |
| 1456226_x_at | Ddr1 | -13 |
| 1433770_at | Dpysl2 | -13 |
| 1451047_at | Itm2a | -13 |
| 1435740_at | Gm10397 | -13 |
| 1431375_s_at | Parva | -13 |
| 1418673_at | Snai2 | -13 |
| 1426397_at | Tgfbr2 | -13 |
| 1417133_at | Pmp22 | -13 |
| 1436363_a_at | Nfix | -13 |
| 1424007_at | Gdf10 | -13 |
| 1427768_s_at | Myl3 | -14 |
| 1416136_at | Mmp2 | -14 |
| 1421106_at | Jag1 | -14 |
| 1437406_x_at | Igfbp4 | -14 |
| 1421917_at | Pdgfra | -14 |
| 1451991_at | Epha7 | -14 |
| 1424133_at | Tmem98 | -14 |
| 1433776_at | Lhfp | -14 |
| 1429313_at | Ror1 | -14 |
| 1450813_a_at | Tnni1 | -14 |
| 1448229_s_at | Ccnd2 | -14 |
| 1451127_at | Vopp1 | -14 |
| 1436920_at | Pcdh17 | -14 |
| 1415973_at | Marcks | -14 |
| 1428361_x_at | Hba-a1 /// Hba-a2 | -14 |
| 1437231_at | Slitrk6 | -15 |
| 1450792_at | Tyrobp | -15 |
| 1421694_a_at | Vcan | -15 |
| 1447640_s_at | Pbx3 | -15 |
| 1435184_at | Npr3 | -15 |
| 1424659_at | Slit2 | -15 |
| 1456060_at | Maf | -15 |
| 1433525_at | Ednra | -15 |
| 1430127_a_at | Ccnd2 | -15 |
| 1455956_x_at | Ccnd2 | -15 |
| 1449351_s_at | Pdgfc | -15 |
| 1438884_at | Shisa3 | -15 |
| 1457871_at | Colec10 | -15 |
| 1436698_x_at | Tmem204 | -15 |
| 1438702_at | Flrt2 | -16 |
| 1451332_at | Zfp521 | -16 |
| 1421811_at | LOC640441 /// Thbs1 | -16 |
| 1426708_at | Antxr2 | -16 |
| 1416211_a_at | Ptn | -16 |
| 1449154_at | Col11a1 | -16 |
| 1450780_s_at | Hmga2 | -16 |
| 1423586_at | Axl | -16 |
| 1430526_a_at | Smarca2 | -16 |
| 1427256_at | Vcan | -16 |
| 1439364_a_at | Mmp2 | -16 |
| 1451446_at | Antxr1 | -16 |
| 1439174_at | --- | -17 |
| 1437152_at | Mex3b | -17 |
| 1424186_at | Ccdc80 | -17 |
| 1451263_a_at | Fabp4 | -17 |
| 1448162_at | Vcam1 | -17 |
| 1426865_a_at | Ncam1 | -17 |
| 1415871_at | Tgfbi | -18 |
| 1448943_at | Nrp1 | -18 |
| 1435435_at | Cttnbp2 | -18 |
| 1448944_at | Nrp1 | -18 |
| 1417065_at | Egr1 | -18 |
| 1425458_a_at | Grb10 | -18 |
| 1439627_at | Zic1 | -18 |
| 1454877_at | Sertad4 | -18 |
| 1440108_at | Foxp2 | -18 |
| 1420553_x_at | Serpina1a | -18 |
| 1434298_at | Zeb2 | -18 |
| 1422851_at | Hmga2 | -18 |
| 1455101_at | Phactr2 | -18 |
| 1452363_a_at | Atp2a2 | -19 |
| 1418450_at | Islr | -19 |
| 1426301_at | Alcam | -19 |
| 1455494_at | Col1a1 | -19 |
| 1460324_at | Dnmt3a | -19 |
| 1456046_at | Cd93 | -19 |
| 1415923_at | Ndn | -19 |
| 1449947_s_at | Zfhx3 | -19 |
| 1423836_at | Zfp503 | -20 |
| 1435382_at | Ndn | -20 |
| 1456632_at | Bcl11a | -20 |
| 1426725_s_at | Ets1 | -20 |
| 1416551_at | Atp2a2 | -20 |
| 1435106_at | Limch1 | -20 |
| 1450992_a_at | Meis1 | -21 |
| 1423477_at | Zic1 | -21 |
| 1429348_at | Sema3c | -21 |
| 1448123_s_at | Tgfbi | -21 |
| 1424131_at | Col6a3 | -21 |
| 1422831_at | Fbn2 | -21 |
| 1434939_at | Foxf1a | -21 |
| 1415935_at | Smoc2 | -22 |
| 1417129_a_at | Meis2 | -22 |
| 1457632_s_at | Meis2 | -22 |
| 1418379_s_at | Gpr124 | -22 |
| 1454881_s_at | Upk3b | -22 |
| 1437784_at | Runx1t1 | -22 |
| 1416846_a_at | Pdzrn3 | -22 |
| 1434366_x_at | C1qb | -22 |
| 1418084_at | Nrp1 | -22 |
| 1448797_at | Elk3 | -22 |
| 1437417_s_at | Gpc6 | -22 |
| 1455096_at | Flrt2 | -22 |
| 1416159_at | Nr2f2 | -22 |
| 1435383_x_at | Ndn | -22 |
| 1419123_a_at | Pdgfc | -23 |
| 1416778_at | Sdpr | -23 |
| 1457072_at | Bcl11a | -23 |
| 1419583_at | Cbx4 | -23 |
| 1427445_a_at | Ttn | -23 |
| 1451989_a_at | Mapre2 | -23 |
| 1437463_x_at | Tgfbi | -23 |
| 1454966_at | Itga8 | -23 |
| 1441137_at | Bicc1 | -23 |
| 1422644_at | Sh3bgr | -23 |
| 1448288_at | Nfib | -24 |
| 1456389_at | Zeb2 | -24 |
| 1448594_at | Wisp1 | -24 |
| 1423547_at | Lyz2 | -24 |
| 1452163_at | Ets1 | -24 |
| 1440147_at | Lgi2 | -25 |
| 1455280_at | Frem1 | -25 |
| 1436937_at | Rbms3 | -25 |
| 1428571_at | Col9a1 | -25 |
| 1416855_at | Gas1 | -25 |
| 1449368_at | Dcn | -25 |
| 1434102_at | Nfib | -25 |
| 1455812_x_at | Vasn | -25 |
| 1438651_a_at | Aplnr | -26 |
| 1434070_at | Jag1 | -26 |
| 1455792_x_at | Ndn | -27 |
| 1449315_at | Odz3 | -27 |
| 1416158_at | Nr2f2 | -27 |
| 1444615_x_at | Runx1t1 | -27 |
| 1415949_at | Cpe /// LOC100046434 | -28 |
| 1450781_at | Hmga2 | -28 |
| 1447643_x_at | Snai2 | -29 |
| 1436717_x_at | Hbb-y | -29 |
| 1438540_at | Col25a1 | -30 |
| 1416740_at | Col5a1 | -30 |
| 1422580_at | Myl4 | -30 |
| 1460000_at | Shisa3 | -30 |
| 1428774_at | Gpc6 | -30 |
| 1454834_at | Nfib | -31 |
| 1458492_x_at | Ntm | -31 |
| 1427233_at | Tshz1 | -31 |
| 1450625_at | Col5a2 | -31 |
| 1418157_at | LOC100046044 /// Nr2f1 | -31 |
| 1448394_at | Myl2 | -31 |
| 1438658_a_at | S1pr3 | -32 |
| 1425575_at | Epha3 | -32 |
| 1437466_at | Alcam | -32 |
| 1422748_at | Zeb2 | -32 |
| 1460574_at | Fat4 | -33 |
| 1449314_at | Zfpm2 | -34 |
| 1448558_a_at | Pla2g4a | -34 |
| 1436823_x_at | Hbb-y | -35 |
| 1460332_at | Pln | -36 |
| 1437405_a_at | Igfbp4 | -37 |
| 1422437_at | Col5a2 | -38 |
| 1458345_s_at | Colec11 | -38 |
| 1417063_at | C1qb | -38 |
| 1427680_a_at | Nfib | -39 |
| 1456250_x_at | Tgfbi | -39 |
| 1455160_at | 2610203C20Rik | -41 |
| 1423835_at | Zfp503 | -41 |
| 1454830_at | Fbn2 | -41 |
| 1437467_at | Alcam | -42 |
| 1437556_at | Zfhx4 | -42 |
| 1452757_s_at | Hba-a1 /// Hba-a2 | -42 |
| 1434881_s_at | Kctd12 | -43 |
| 1452762_at | Rbms3 | -44 |
| 1424890_at | Bnc1 | -45 |
| 1449071_at | Myl7 | -46 |
| 1436938_at | Rbms3 | -47 |
| 1448823_at | Cxcl12 | -47 |
| 1423756_s_at | Igfbp4 | -49 |
| 1460208_at | Fbn1 | -51 |
| 1415927_at | Actc1 /// LOC100048431 | -52 |
| 1437726_x_at | C1qb | -52 |
| 1438531_at | A730054J21Rik | -52 |
| 1436041_at | LOC100046086 | -52 |
| 1435120_at | --- | -53 |
| 1455426_at | Epha3 | -57 |
| 1437347_at | Ednrb | -60 |
| 1449939_s_at | Dlk1 | -65 |
| 1417574_at | Cxcl12 | -69 |
| 1425476_at | Col4a5 | -70 |
| 1448554_s_at | Myh6 /// Myh7 | -74 |
| 1452114_s_at | Igfbp5 | -74 |
| 1423606_at | Postn | -75 |
| 1423607_at | Lum | -86 |
| 1433919_at | Asb4 | -88 |
| 1436996_x_at | Lyz1 | -93 |
| 1436970_a_at | Pdgfrb | -95 |
| 1439426_x_at | Lyz1 | -99 |
| 1423422_at | Asb4 | -99 |
| 1448254_at | Ptn | -109 |
| 1450757_at | Cdh11 | -119 |
| 1459749_s_at | Fat4 | -122 |
| 1424967_x_at | Tnnt2 | -140 |
| 1427884_at | Col3a1 | -228 |
| 1448826_at | Myh6 | -294 |
| 1427883_a_at | Col3a1 | -308 |
| 1418726_a_at | Tnnt2 | -338 |
