# Supplementary material for: Fam40b is required for lineage commitment of murine embryonic stem cells
Source: Cell Death Dis. 2014 Jul 10;5(7):e1320–. doi: 10.1038/cddis.2014.273 (PMC4123067; doi:10.1038/cddis.2014.273)
Supplement: Supplementary Table S2 [file cddis2014273x3.doc]

Supplementary table S2. The file shows the primers applied for RT-PCR and qPCR analysis

| Gene | Left primer | Right primer |
| --- | --- | --- |
| Fam40b | ACCATCCAGGATTTGCCAGAGC | ATTGAGCAGCCTCAGGAGGTTG |
| Gapdh | CATCACTGCCACCCAGAAGACTG | ATGCCAGTGAGCTTCCCGTTCAG |
| Rag1ap1 | GTTCCTGCCTTTTCTCACCACG | GCTTCTGAGGACTGTAGTGCAG |
| Dnmt3b | CGCACAACCAATGACTCTGCTG | GGTGACTTCAGAAGCCATCCGT |
| Hat1 | GATGGAGCTACGCTCTTTGCGA | GCCCTGACCTTGAAATGGAGTC |
| Lin28 | GGTCTGGAATCCATCCGTGTCA | TCCTTGGCATGATGGTCTAGCC |
| Nanog | GAACGCCTCATCAATGCCTGCA | GAATCAGGGCTGCCTTGAAGAG |
| Pou5f1 | CAGCAGATCACTCACATCGCCA | GCCTCATACTCTTCTCGTTGGG |
| Nodal | GGTGGACTTCAACCTGATTGGC | GGTTGGTATCGTTTCAGCAGGC |
| Sox2 | AACGGCAGCTACAGCATGATGC | CGAGCTGGTCATGGAGTTGTAC |
